# Metabolic and fitness determinants for in vitro growth and intestinal colonization of the bacterial pathogen *Campylobacter jejuni*

**DOI:** 10.1371/journal.pbio.2001390

**Published:** 2017-05-19

**Authors:** Beile Gao, Hanne Vorwerk, Claudia Huber, Maria Lara-Tejero, Juliane Mohr, Andrew L. Goodman, Wolfgang Eisenreich, Jorge E. Galán, Dirk Hofreuter

**Affiliations:** 1 Department of Microbial Pathogenesis, Yale University School of Medicine, New Haven, Connecticut, United States of America; 2 Institute for Medical Microbiology and Hospital Epidemiology, Hannover Medical School, Hannover, Germany; 3 Lehrstuhl für Biochemie, Technische Universität München, Garching, Germany; 4 Microbial Sciences Institute, Yale University School of Medicine, New Haven, Connecticut, United States of America; Brigham and Women's Hospital, United States of America

## Abstract

*Campylobacter jejuni* is one of the leading infectious causes of food-borne illness around the world. Its ability to persistently colonize the intestinal tract of a broad range of hosts, including food-producing animals, is central to its epidemiology since most infections are due to the consumption of contaminated food products. Using a highly saturated transposon insertion library combined with next-generation sequencing and a mouse model of infection, we have carried out a comprehensive genome-wide analysis of the fitness determinants for growth in vitro and in vivo of a highly pathogenic strain of *C*. *jejuni*. A comparison of the *C*. *jejuni* requirements to colonize the mouse intestine with those necessary to grow in different culture media in vitro, combined with isotopologue profiling and metabolic flow analysis, allowed us to identify its metabolic requirements to establish infection, including the ability to acquire certain nutrients, metabolize specific substrates, or maintain intracellular ion homeostasis. This comprehensive analysis has identified metabolic pathways that could provide the basis for the development of novel strategies to prevent *C*. *jejuni* colonization of food-producing animals or to treat human infections.

## Author summary

There is accumulating evidence that in addition to canonical virulence factors such as toxins, adhesins, or invasins, bacterial pathogens utilize specific metabolic traits to colonize and proliferate within their hosts, a concept that is increasingly referred to as “nutritional virulence”. We have used transposon insertion mutagenesis combined with next-generation sequencing, a mouse model of infection, isotopologue profiling, and metabolic flow analysis to obtain a comprehensive view of the metabolic requirements for the intestinal colonization of *C*. *jejuni*, a leading cause of food-borne gastroenteritis in industrialized countries. This information could provide the basis to control *C*. *jejuni* colonization of food-producing animals or the human host.

## Introduction

*Campylobacter jejuni* subsp. *jejuni* (*C*. *jejuni*) is one of the most common causes of infectious food-borne illness in industrialized countries [1,2]. The high incidence of this pathogen is due to its ability to persistently colonize the intestinal tract of food-producing animals. Contaminated food products—in particular, poultry meat—become a source of *C*. *jejuni* infection when improperly handled or undercooked [3]. While asymptomatic in most vertebrates, in humans, *C*. *jejuni* infection often leads to acute, although self-limiting, gastroenteritis [4]. Rarely, infections with *C*. *jejuni* lead to a sequelae known as Guillain-Barre syndrome, which is characterized as a serious neurodegenerative disorder [5]. A characteristic feature of *C*. *jejuni* that distinguishes it from other common enteropathogenic bacteria is the paucity of homologs of virulence factors that in other pathogens are engaged in specific interaction with the host [6,7]. In fact, *C*. *jejuni* has arguably more in common with commensal intestinal microbiota than with enteric pathogens. This is consistent with the observation that, other than in humans, the persistent presence of *C*. *jejuni* in the gut does not lead to pathology [8]. Why and how *C*. *jejuni* infection in humans leads to disease is very poorly understood, but it is expected that its ability to colonize and replicate within the intestinal tract to reach significant numbers must be central to its pathogenesis. Several studies have identified genes that are important for *C*. *jejuni* intestinal colonization using different animal models of infection. Other than genes required for motility or the modification of surface structures (e.g., protein glycosylation), the vast majority of genes identified to date as required for colonization are involved in the acquisition and metabolism of essential nutrients [7,9–12]. Therefore, deciphering the metabolic requirements of *C*. *jejuni* is central to the understanding of its ability to colonize a host and potentially cause disease. In fact, the understanding of the metabolism of bacterial pathogens during infection is quickly emerging as an extremely important area of research. While the basic metabolism of model bacteria during their growth in vitro has been extensively studied, knowledge of the metabolic requirements of bacterial pathogens during infection has lagged behind [13]. Indeed, it is becoming increasingly clear that the functional purpose of some virulence factors that specifically target host processes is to increase the availability of crucial nutrients or to facilitate their acquisition for bacterial growth. Therefore, the concept of “nutritional virulence factors” has been proposed to describe such pathogenic determinants [14–17].

Although most of the *C*. *jejuni* colonization determinants known to date have been discovered by candidate-mutant experimental approaches, there have been some attempts to comprehensively identify such determinants using genome-wide approaches [18–20]. However, because of technical limitations in the approaches or animal models used, those studies have not been comprehensive. The availability of high-throughput nucleotide sequencing technologies coupled to transposon mutagenesis has provided a powerful tool to interrogate highly saturated mutant libraries of insertion mutants for specific phenotypes [21–23]. This approach allows not only the comprehensive identification of virulence or colonization determinants but also the simultaneous measurement of the relative fitness cost resulting from the inactivation of potentially every nonessential gene under various environmental conditions. When applied to the understanding of metabolic requirements, such comparative analysis can provide a much more encompassing view of the relative importance of specific metabolic pathways under the conditions examined. An essential prerequisite for the application of high-throughput approaches to interrogate genomic libraries is the absence of bottlenecks that could limit the depth of coverage of the mutagenesis screen. Since in most animal models of infection *C*. *jejuni* cannot be recovered in large numbers [24], this has been a major limitation in previous attempts to broadly interrogate mutant libraries for their ability to colonize the intestine [19,25]. Recently, a mouse model of infection has been described that allows the replication of *C*. *jejuni* to large numbers [26], thereby overcoming previous limitations for the application of high-throughput genome-wide analyses. We report here the use of this animal model in combination with transposon mutagenesis and next-generation sequencing to comprehensively interrogate a highly saturated transposon insertion library of *C*. *jejuni* 81–176 for its ability to colonize the intestine. To provide a more robust framework for the interpretation of these results, we have also examined the mutant library after growth under different in vitro conditions. The combination of this extensive genetic approach with isotopologue profiling, metabolic flow examination, and the analysis of specific mutant strains amounted to the most complete analyses of the metabolic determinants necessary for a bacterial pathogen to colonize a mammalian host to date. Importantly, this analysis has identified key metabolic pathways that could be targeted for the development of novel strategies to prevent *C*. *jejuni* infection.

## Results and discussion

### Genome wide analyses of *C*. *jejuni* fitness determinants in rich and defined minimal media

**Fig 1:**
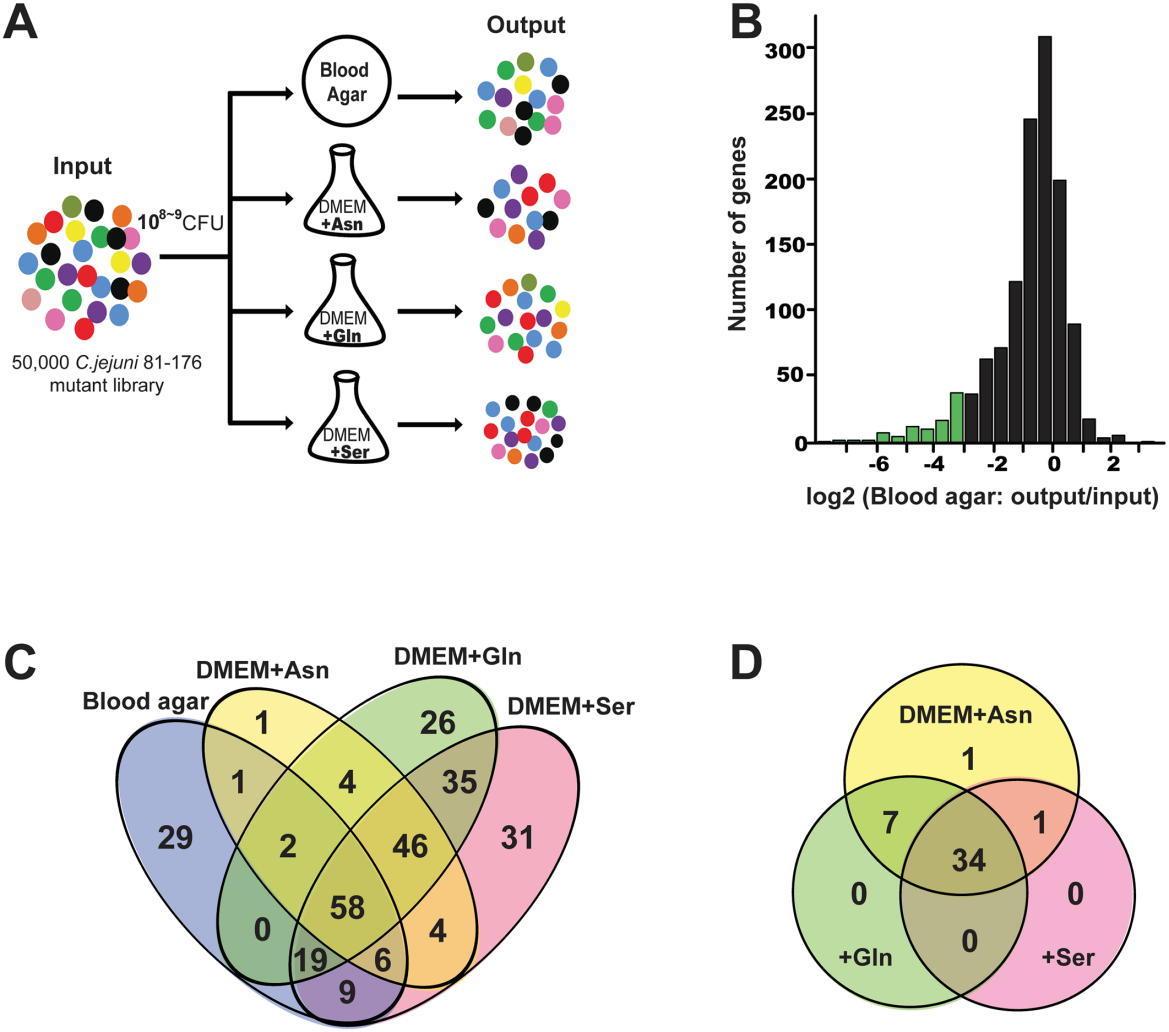
*C*. *jejuni* 81–176 fitness determinants identified by insertion sequencing (INSeq) analyses. (A) Diagram of the INSeq strategy used in these studies. (B) Histogram depicting the number of genes (*y* axis) that exhibited the indicated log_2_ (fold change [output/input]) change (*x* axis) in the numbers of transposon insertions recovered after growth on solid rich medium relative to the number of transposon insertions in the original inoculum. Areas colored with green represent genes whose number of transposon insertions showed a significant decrease after growth on solid rich medium. (C) Venn diagram showing the relationship between genes required for growth under different culture conditions identified in these studies. (D) Venn diagram depicting the relationship between genes whose inactivation led to increased growth in Dulbecco’s Modified Eagle Medium (DMEM) supplemented with different amino acids. Twenty-two out of 34 genes whose mutation led to increased growth encode proteins associated with the flagellar motility system (see S2 Table for details). Asn, asparagine; CFU, colony-forming unit; Gln, glutamine; Ser, serine.

We recently reported the construction of a highly saturated and randomly dispersed transposon mutant library composed of approximately 50,000 individual insertions with an average density of 31 insertions/kb across the *C*. *jejuni* 81–176 genome [27]. Analysis of the library indicated that 90% of the 1,758 predicted open reading frames in the genome and its plasmids *pTet* and *pVir* harbored transposon insertions, whereas the remaining 10% of the genes were presumably essential under the conditions used to generate the library [27]. We used this transposon mutant library to interrogate the fitness of the different *C*. *jejuni* mutants after growth in solid rich (blood agar) or defined liquid medium supplemented with asparagine (Asn), glutamine (Gln), or serine (Ser) as the main carbon sources (Fig 1).

Comparison of the relative number of each transposon mutant remaining after 48 hours of growth under the different conditions with the number in the original inoculum identified 58 genes whose inactivation resulted in a growth defect under all conditions tested (Fig 1C, S1 and S2 Tables). Most of these genes are involved in basic metabolic processes such as nutrient transport and utilization, respiration, response to oxidative stress, cell envelope biogenesis, DNA replication and repair, translation, and protein turnover. This group of mutants also includes insertions in 13 genes whose functions are unknown. Insertions in 46 genes resulted in mutants with growth defects in defined liquid but not in solid rich media (Fig 1C and S2 Table). Among those, we identified mutations in genes involved in proline (*proABC*), biotin (*bioAFD*), and purine and pyrimidine (*carB*, *pyrBF*, and *purEFHLMNQS*) biosynthesis. These observations validated our experimental approach since the defined medium used in the screen lacked these substrates. The analysis of the results described below combined with isotopologue profiling and metabolic flow analysis provides a comprehensive view of the metabolic requirements for *C*. *jejuni* to grow in rich and defined media.

#### 1. *C*. *jejuni* has restricted catabolic capacity but broad biosynthetic ability: Unexpected prominence for Serine utilization

**Fig 2:**
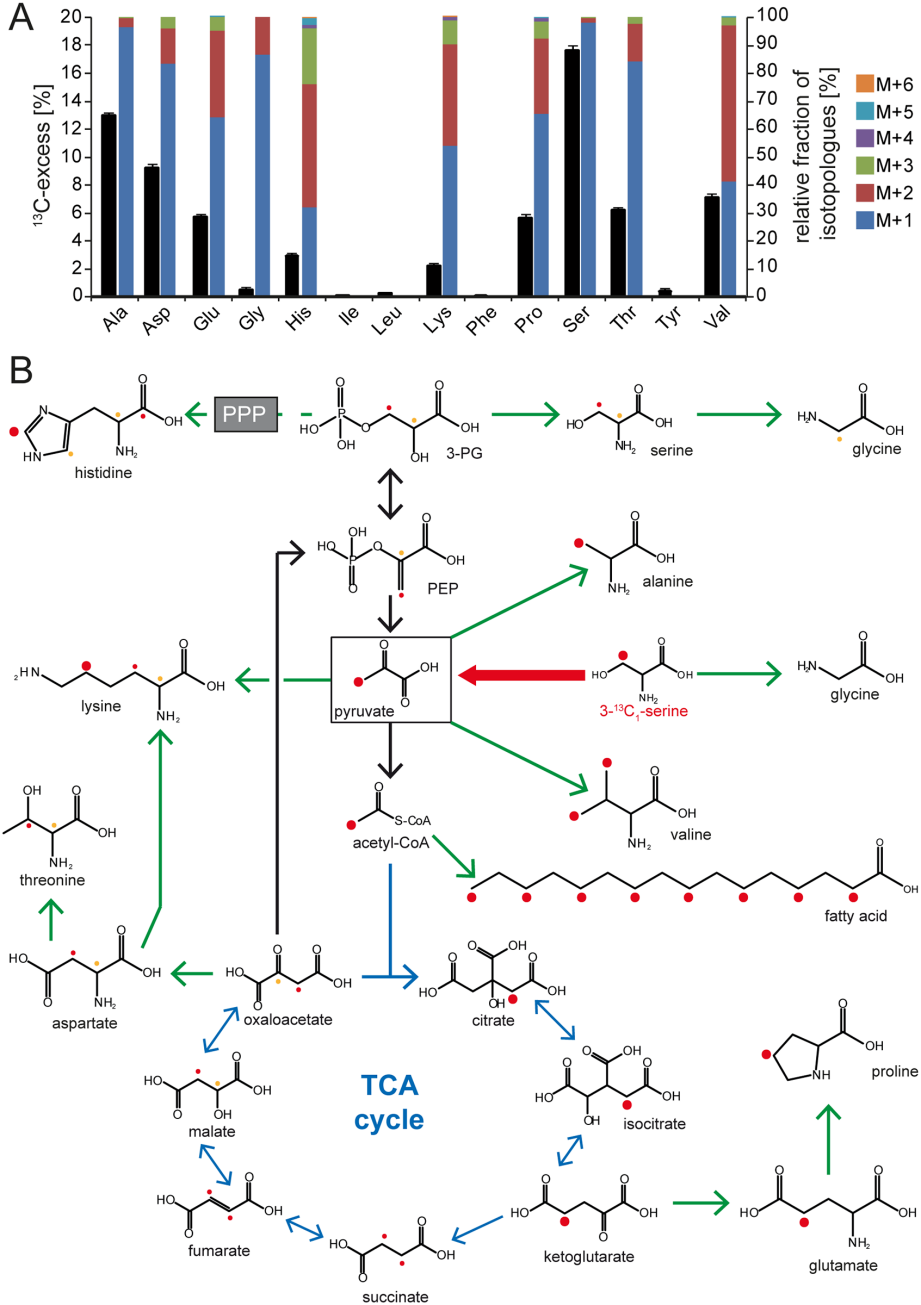
Biosynthetic capacities of *C*. *jejuni* 81–176 upon catabolism of [3-^13^C_1_]Ser. The intermediary metabolism of *C*. *jejuni* 81–176 was investigated through isotopologue profiling with ^13^C-labelled Ser. (A) Overall ^13^C-excess and relative fractions of ^13^C-labeled isotopologues in protein-derived amino acids gained by acidic hydrolysis of *C*. *jejuni* 81–176 cells after cultivation in Dulbecco’s Modified Eagle Medium (DMEM) with [3-^13^C_1_]Ser as determined by gas-chromatography/mass-spectrometry (GC/MS) analysis. The colored boxes indicate the relative contributions (%) of isotopologues with 1, 2, 3, 4, 5, and 6 ^13^C-atoms indicated as M+1, M+2, M+3, M+4, M+5, and M+6, respectively. Values are the means ± standard deviation (SD) of 6 measurements (see S11 Table). (B) Overview of the anabolism in *C*. *jejuni* 81–176 fueled by the catabolism of [3-^13^C_1_]Ser. The dots illustrate the ^13^C-carbon flux from [3-^13^C_1_]Ser within the indicated molecules. Because of stereoisometry, the positioning of the ^13^C-atoms within succinate and fumarate is indistinguishable; thus, the resulting possibilities of the ^13^C-positions are indicated in red and orange as a 50% labeling probability for each. Green arrows display the biosynthetic pathways confirmed through isotopologue profiling. Acetyl-CoA, acetyl coenzyme A; Ala, alanine; Asp, asparagine; Glu, glutamic acid; Gly, glycine; His, histidine; Ile, isoleucine; Leu, leucine; Lys, lysine; Phe, phenylalanine; PEP, phosphoenolpyruvic acid; PPP, pentose phosphate pathway; Pro, proline; Ser, serine; TCA, tricarboxylic acid; Thr, threonine; Tyr, tyrosine; Val, valine.

It is well established that *C*. *jejuni* has no glycolytic capacity and can utilize only a limited number of amino acids (aspartic acid [Asp], Asn, glutamic acid [Glu], Gln, proline [Pro], and Ser) as carbon and energy sources [28–31]. Our screen revealed that *sdaC* mutants, which are unable to transport Ser and show a reduced serine dehydratase activity [32], have a severe growth disadvantage on nutrient-rich blood agar plates. In contrast, inactivation of the genes *putP* (CJJ81176_1495), *paq* (CJJ81176_0492–0494), or *peb* (CJJ81176_0926–0929), which are required for the uptake of Pro, Gln, and Asp and Glu, respectively [11], did not hinder the in vitro growth of *C*. *jejuni* 81–176 in rich medium (S1 Table). These results revealed an unexpected prominence for Ser utilization in *C*. *jejuni* metabolism even in the presence of other nutrients. To investigate the role of Ser in fueling the intermediary metabolism and anabolism of *C*. *jejuni* 81–176, we used ^13^C-isotopologue profiling (see “Material and methods”). After *C*. *jejuni* cultivation in defined Dulbecco’s Modified Eagle Medium (DMEM) containing [3-^13^C_1_]Ser, we detected significant ^13^C-incorporation in 10 of 15 detectable protein-derived amino acids, with no or insignificant ^13^C-labeling of arginine (Arg), isoleucine (Ile), leucine (Leu), phenylalanine (Phe), and tyrosine (Tyr) (Fig 2A). These results suggest that these amino acids are preferentially imported from the culture medium. The deamination of [3-^13^C_1_]Ser catalyzed by the serine dehydratase SdaA generated [3-^13^C_1_]pyruvate and the robust ^13^C-labeling of Glu, Pro, and Asp (Fig 2B), which are derived from α-ketoglutarate (Glu and Pro) and oxaloacetate (OAA). These findings showed that a significant amount of catabolized Ser is converted to pyruvate (Pyr) and subsequently to acetyl coenzyme A (acetyl-CoA), thus feeding into the tricarboxylic acid (TCA) cycle. Although no gene encoding a putative alanine (Ala) aminotransferase that could convert Pyr to Ala is apparent in the genome of *C*. *jejuni*, single-labeled [3-^13^C_1_]Ala showed the second-highest ^13^C-excess after Ser (Fig 2A). The important role of Pyr in the anabolism of *C*. *jejuni* is further illustrated by the multilabeled lysine (Lys), generated through the condensation of [3-^13^C_1_]Pyr with ^13^C-labeled Asp, and the (M+2)-labeling of valine (Val), generated from two [3-^13^C_1_]Pyr molecules. In contrast to Val, the 2 other branched-chain amino acids Leu and Ile did not show significant ^13^C-inocorporation. This result correlates with a previous study demonstrating that Val is taken up by the leucine-isoleucine-valine (LIV) transport system to a lesser extent than Leu and Ile [33]. Therefore, an increased Val biosynthesis appears to be required to fulfill the *C*. *jejuni* needs for this amino acid. We detected robust ^13^C-labeling of histidine (His), which is synthesized from phosphoribosyl pyrophosphate (PRPP). This sugar is generated from the Emden-Meyerhof-Parnas (EMP) pathway intermediates glyceraldehyde-3-phosphate (GA3P) and fructose-6-phosphate through the interconversion reactions of the reductive pentose phosphate pathway (PPP) branch, which demonstrates that *C*. *jejuni* 81–176 harbors an active gluconeogenesis and nonoxidative PPP (see below) (Fig 2B).

We also examined the ^13^C-incorporation into polar metabolites after incubation of *C*. *jejuni* with [3-^13^C_1_]Ser and found a similar ^13^C-excess and distribution of isotopologue fractions in unbound or protein-derived Ala, Asp, glycine (Gly), methionine (Met), threonine (Thr), and Val (S1 Fig). Uracil was among the substrates with the highest ^13^C-excess, reflecting the importance of de novo pyrimidine biosynthesis when *C*. *jejuni* is cultivated in defined liquid medium. Accordingly, insertion mutants with inactivated purine and pyrimidine biosynthesis genes, such as *purA/C*, *pyrD/E*, and *carA*, exhibited strong growth defects after 48 hours of cultivation (S2 Table). The fatty acids C_12_-lauric and C_16_-palmitic acid showed the highest ^13^C-excess in our isotopologue profiling analysis, indicating that fatty acid biosynthesis is very active in *C*. *jejuni*. This observation is also consistent with the pronounced carbon flow from [3-^13^C_1_]Ser to acetyl-CoA via Pyr and the observed serial condensations of [2-^13^C_1_]acetyl-CoA molecules during the fatty acid elongation (Fig 2B and S1 Fig). Moreover, our insertion sequencing (INSeq) analysis indicated that the fatty acid biosynthesis genes *fabDFGHZ* seem to be essential for *C*. *jejuni*. Taken together, our analysis illustrates the highly versatile anabolic capacity of *C*. *jejuni* as exemplified by its competence in the de novo synthesis of most essential compounds, fueling its anabolism by the catabolism of a very narrow spectrum of carbon sources.

#### 2. *C*. *jejuni* exhibits uptake capacity for noncatabolized amino acids that are directly incorporated into synthesized proteins

Our in vitro INSeq analysis identified numerous transposon insertions in genes encoding biosynthetic pathways for the amino acids Arg, Asp, cysteine (Cys), Glu, Gln, His, Ile, Leu, Lys, Met, Phe, Pro, Ser, Thr, tryptophan (Trp), and Val, which resulted in no measurable fitness cost (S1 Table, S2A Fig). These results indicate that through as of yet unidentified transporters, *C*. *jejuni* must be able to take up amino acids like Arg, Cys, His, Lys, Phe, or Thr, which cannot be utilized as an energy source but can be directly used for protein synthesis. To confirm such an extended amino acid uptake capacity, we constructed specific *C*. *jejuni* mutants defective for the synthesis of branched-chain amino acids (*ilvD*), Lys (*lysA*), Arg (*argD*), or Thr (*thrB*) and tested their growth in vitro. We found no substantial defects in the growth of these mutant strains in defined amino acid-rich medium (S2B Fig). Moreover, gas-chromatography/mass-spectrometry (GC/MS) analysis showed that after 24 hours of cultivation of *C*. *jejuni* 81–176 in defined amino acid-rich medium, Asp, Cys, Glu, Pro, Ser, Met, and Thr were significantly depleted from the culture medium (S3 Fig). The ability to take up noncatabolizable amino acids like Cys, Met, and Thr most likely provides a fitness advantage to *C*. *jejuni* by reducing the energy cost associated with the de novo synthesis of these amino acids [34].

#### 3. Cofactors and vitamins in *C*. *jejuni* metabolism

Besides its capacity to synthesize amino acids, genome sequence analysis indicates that *C*. *jejuni* 81–176 has the ability to synthesize several enzyme cofactors [35]. Transposon insertions within genes involved in biosynthesis of nicotinamide adenine dinucleotide (NAD; *nadDE*), heme (*hemABCDENHL*), flavin/riboflavin (*ribABEH*), folate (*folCDEP*), thiamine (*thiDEFGHS*), or pyridoxine-5′-phosphate (PLP; *pdxAJ*, *dxs*) were absent or present at very low numbers in our library (S1 Table). Consequently, these genes are either essential or at least crucial for the growth of *C*. *jejuni* 81–176 under the conditions of our screen. Although panthothenate is required for coenzyme A synthesis, our analysis identified a large number of insertions within the pantothenate biosynthesis genes *panBCD*. Mutations in these genes did not show growth defect in vitro, indicating that *C*. *jejuni* 81–176 must be able to take up pantothenate from the growth medium. This result is in agreement with the recent observation that culture medium supplemented with pantothenate enhances the growth of *C*. *jejuni* [36].

### Genome-wide analysis of *C*. *jejuni* metabolic determinants necessary for intestinal colonization in a mouse model of infection

**Fig 3:**
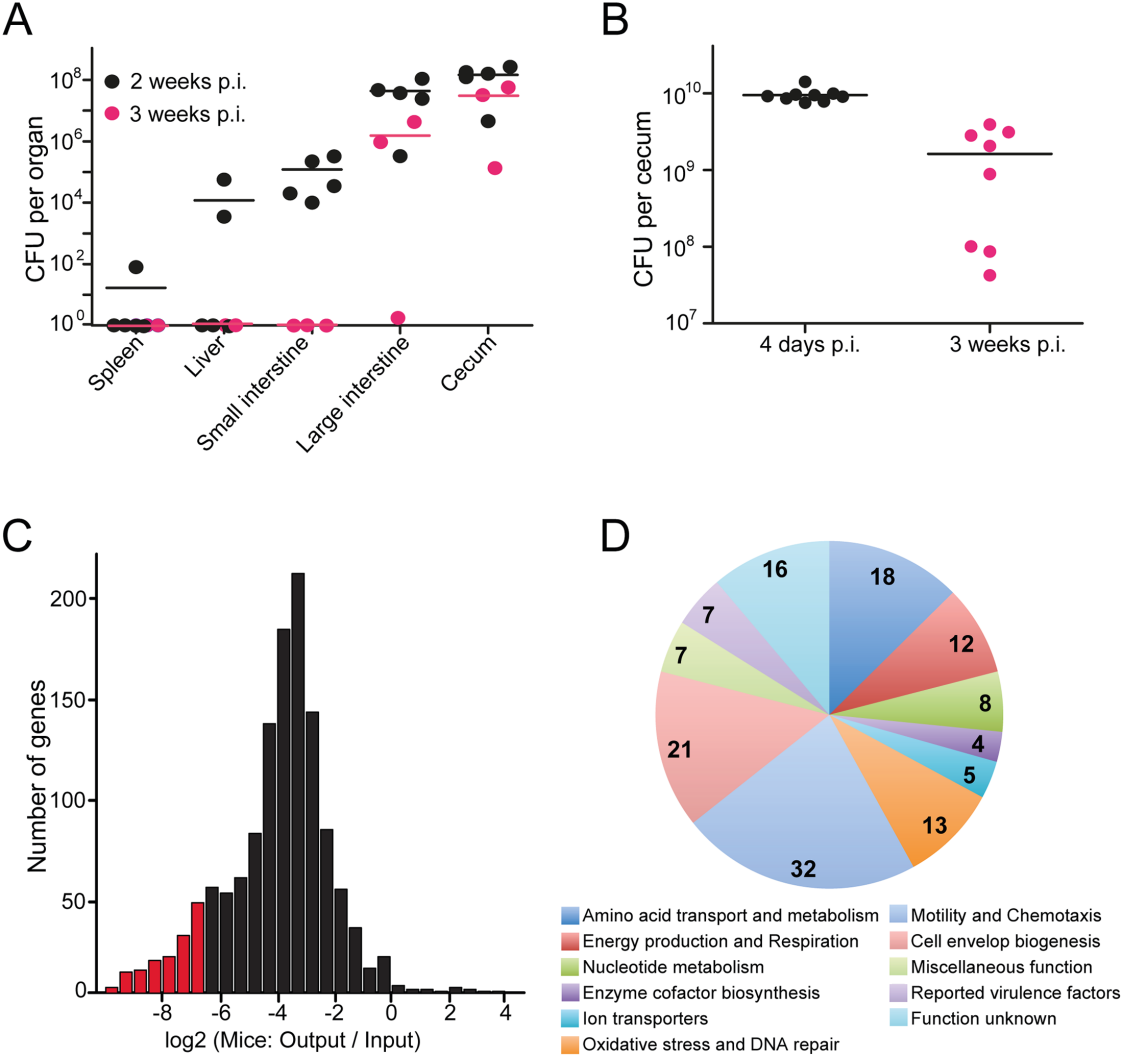
Colonization determinants of *C*. *jejuni* 81–176 identified by insertion sequencing (INSeq) analyses. Mice rendered dysbiotic by antibiotic treatment were orally infected with wild-type *C*. *jejuni* 81–176 (A) or a library of transposon-insertion mutants (B), and the number of colony-forming units (CFUs) in the ceca of infected animals was determined as indicated in “Materials and methods.” (C) Histogram depicting the number of genes (*y* axis) that exhibited the indicated log_2_ (fold change [output/input]) change (*x* axis) in the numbers of transposon insertions recovered from infected mice relative to the number of transposon insertions in the original inoculum. Areas colored with red represent genes whose number of transposon insertions showed a significant decrease after mouse infection. The calculation of log_2_ (fold change [output/input]) is based on the read numbers of insertional mutants of each gene presented in S3 Table. (D) Functional categories of the *C*. *jejuni* 81–176 genes whose inactivation led to mouse intestinal colonization defects as measured by INseq analysis. The genes belonging to each functional category can be found in Table 1. p.i., post infection.

It was recently reported that mice, rendered dysbiotic by antibiotic treatment, could be stably colonized by *C*. *jejuni* [26]. We found that, predictably, antibiotic treatment drastically reduced though did not eliminate the bacterial load in the intestinal tract (S4 Fig). More importantly, we found that, consistent with previous reports [26], *C*. *jejuni* strain 81–176 can robustly colonize the intestinal tract of dysbiotic mice for up to 3 weeks after oral infection (Fig 3A). Large numbers of *C*. *jejuni* colony-forming units (CFUs) were recovered from the large intestine and cecum of infected animals as early as 4 days after infection and up to 21 days post infection, although colonization of systemic tissues was inefficient. Consequently, this infection model is suitable to screen a library with a large number of transposon insertion mutants for their ability to colonize the intestine as a strategy to identify metabolic determinants necessary for *C*. *jejuni* growth in this compartment. After oral inoculation of dysbiotic C57BL/6 mice with the *C*. *jejuni* 81–176 transposon insertion mutant library, the infected animals were killed 4, 7, or 21 days after infection, and *C*. *jejuni* CFUs were recovered from the cecum for INSeq analysis. About 10^10^ *C*. *jejuni* CFUs were recovered from the cecum of infected mice 4 days after infection, and between 10^8^ and 10^9^ CFUs were recovered 21 days after infection (Fig 3B). INSeq analysis of the inoculum revealed transposon insertions in 1,323 out of 1,758 predicted genes of *C*. *jejuni* 81–176. Since only insertions within the first 80% of the coding sequence were considered in the analysis, it is likely that all these insertions resulted in inactivation of the targeted genes. Analysis of the mutants recovered 21 days post infection revealed a marked increase in insertions within the pVir and pTet plasmids, with a marked reduction in the representation of the mutant pool (S5 Fig). The reasons for the “blooming” of these mutants late in infection are unclear, although our library already contained a preponderance of insertions within the resident plasmids, presumably because differences in DNA superhelicity may favor insertions into plasmid genes. Nevertheless, the blooming of plasmid insertion mutants observed at day 21 precluded the use of this time point to analyze *C*. *jejuni* colonization determinants. Examination of the mutant pool isolated 4 days after infection revealed the presence of an average of 935 mutants per animal (approximately 70% of the inoculum pool), and no indication of blooming since the relative number of insertions within plasmid or chromosomal genes recovered from the intestine was similar to their ratio in the inoculum (S5 Fig and S3 Table). Therefore, we chose this time point to identify genes involved in the initiation of *C*. *jejuni* colonization of the intestinal tract. The distribution of log2 values for the number of insertions in most genes showed an overall reduction in comparison with the inoculum (Fig 3C). We observed a similar distribution even when the data were normalized by an alternative approach (S6 Fig and S4 Table) (see “Materials and methods”). Although the reasons for this downshift in the population are not known and likely to be multifactorial, it is not unprecedented since it has been observed in previous similar studies [37]. Consequently, the fitness defect of a given mutant could not be confidently derived by a given cutoff in the log2 ratio of the difference between the number of insertions recovered from the intestine versus the number in the original inoculum. Instead, we identified genes with fitness defects by applying statistical analysis to determine the likelihood that the differences in log2 ratios for any specific gene could have happened by chance (see “Materials and methods”). This analysis identified 143 genes that showed a significantly decreased number of insertions within the mutant pool recovered from the intestine relative to the inoculum (Fig 3C and 3D and Table 1). We found that insertions located immediately downstream of 126 out of the 143 genes identified did not result in attenuating phenotypes indicating that the transposon insertions do not result in polar effects on downstream genes (S5 Table). Mutations in 101 of these genes did not result in a fitness reduction when grown in rich medium (Table 1), suggesting that they may be specifically required for initiation of intestinal colonization. Mutations in the remaining 42 genes resulted in different degrees of fitness loss when *C*. *jejuni* was grown in vitro (Table 1).

**Table 1:** List of *C*. *jejuni* 81–176 genes whose insertion mutants resulted in a significant mouse colonization defect.

Gene ID	Symbol		Mice log2(fc)^1^	Mice q-value	In vitro log2(fc)^1^	Annotation
**Amino acid transport and metabolism**						
CJJ81176_0186		aspB-2	−7.52	0.00	−2.36	aspartate aminotransferase
CJJ81176_0296	*	ilvE	−6.86	0.01	−1.09	branched-chain amino acid aminotransferase
CJJ81176_0308	*	serB	−6.78	0.01	−0.78	phosphoserine phosphatase
CJJ81176_0539	*	ctpA	−6.60	0.03	−0.50	carboxyl-terminal protease
CJJ81176_0603	*	ilvH	−6.29	0.04	−0.73	acetolactate synthase 3 regulatory subunit
CJJ81176_0660		ilvC	−6.52	0.04	−4.76	ketol-acid reductoisomerase
CJJ81176_0783	*	aspB	−7.50	0.00	0.96	aspartate aminotransferase
CJJ81176_0900	*	serA	−6.55	0.04	−0.06	D-3-phosphoglycerate dehydrogenase
CJJ81176_0494	*	paqQ	−6.50	0.04	−0.89	amino acid ABC transporter
CJJ81176_0793	*	metQ	−7.11	0.00	−0.91	methionine ABC transporter
CJJ81176_0795		metN	−6.90	0.01	−6.79	methionine ABC transporter
CJJ81176_0926	*	peb1	−7.27	0.00	−0.38	amino acid ABC transporter permease protein
CJJ81176_0927	*	peb1	−7.29	0.00	−0.60	amino acid ABC transporter permease protein
CJJ81176_0928	*	pebA	−7.40	0.00	−0.83	ABC transporter aspartate/glutamate-binding
CJJ81176_1035		livM	−6.43	0.04	−3.15	high affinity branched-chain aa ABC transporter
CJJ81176_1038		livJ	−6.70	0.02	−5.70	high affinity branched-chain aa ABC transporter
CJJ81176_1117	*	pepF	−7.67	0.00	−0.24	oligoendopeptidase F
CJJ81176_1416	*	–	−6.78	0.01	−0.24	putative family C26 endopeptidase
**Energy production and respiration**						
CJJ81176_0262	*	canB	−7.22	0.00	−1.27	carbonic anhydrase
CJJ81176_0397	*	–	−7.36	0.00	−1.33	2-hydroxyacid dehydrogenase
CJJ81176_0415		pyk	−6.64	0.02	−2.46	pyruvate kinase
CJJ81176_0433		frdA	−7.01	0.00	−2.71	fumarate reductase flavoprotein subunit
CJJ81176_0557	*	mdh	−6.34	0.11	0.47	malate dehydrogenase
CJJ81176_0558	*	sucC	−6.82	0.01	−0.43	succinyl-CoA synthetase subunit beta
CJJ81176_0711	*	pta	−6.99	0.00	−1.11	phosphate acetyltransferase
CJJ81176_0712	*	ackA	−7.99	0.00	−0.51	acetate kinase
CJJ81176_1039		–	−7.08	0.00	−2.98	cytochrome c family protein
CJJ81176_1076	*	–	−6.56	0.04	−1.56	carbon-nitrogen family hydrolase
CJJ81176_1304	*	mez	−7.42	0.00	−1.41	NADP-dependent malic enzyme, truncation
CJJ81176_1675		gltA	−9.12	0.00	−2.30	citrate synthase
**Nucleotide metabolism**						
CJJ81176_0305	*	carB	−9.13	0.00	−1.84	carbamoyl phosphate synthase large subunit
CJJ81176_0404	*	pyrF	−7.41	0.00	−1.94	orotidine 5'-phosphate decarboxylase
CJJ81176_0541	*	purS	−6.48	0.04	−0.76	phosphoribosylformylglycinamidine synthase
CJJ81176_0542	*	purQ	−6.80	0.01	−0.51	phosphoribosylformylglycinamidine synthase
CJJ81176_0978	*	purL	−7.57	0.00	−1.02	phosphoribosylformylglycinamidine synthase
CJJ81176_1053	*	fedC	−6.89	0.01	−0.61	adenylosuccinate lyase
CJJ81176_1116	*	pyrB	−7.58	0.00	−0.85	aspartate carbamoyltransferase
CJJ81176_1486		carA	−6.72	0.01	−6.72	carbamoyl phosphate synthase small subunit
**Enzyme cofactor biosynthesis**						
CJJ81176_0197		moaA	−6.60	0.03	−6.63	molybdenum cofactor biosynthesis protein A
CJJ81176_0328		bioF	−7.01	0.00	−2.02	8-amino-7-oxononanoate synthase
CJJ81176_0329		bioA	−6.82	0.01	−2.59	transaminase
CJJ81176_1510	*	moaE	−6.37	0.04	0.48	molybdopterin converting factor, subunit 2
**Ion transporters**						
CJJ81176_0177		znuB	−6.60	0.03	−4.77	zinc ABC transport permease
CJJ81176_0179		znuA	−6.28	0.04	−4.97	periplasmic zinc binding protein
CJJ81176_0942	*	–	−6.76	0.01	−1.68	Neurotransmitter/sodium symporters
CJJ81176_1300	*	ktrB	−8.79	0.00	−1.57	TrkH family potassium uptake protein
CJJ81176_1301	*	ktrA	−7.75	0.00	−0.89	potassium uptake protein TrkA, putative
**Oxidative stress and DNA repair**						
CJJ81176_0055		recJ	−7.72	0.00	−2.76	single-stranded-DNA-specific exonuclease
CJJ81176_0347	*	xseA	−7.15	0.00	−1.07	exodeoxyribonuclease VII large subunit
CJJ81176_0366		uvrA	−7.40	0.00	−2.89	excinuclease ABC subunit A
CJJ81176_0622	*	–	−7.68	0.00	−0.51	DNA/RNA nonspecific endonuclease
CJJ81176_0670	*	recN	−7.46	0.00	−0.37	DNA repair protein RecN
CJJ81176_0689	*	hslU	−6.50	0.04	−1.85	ATP-dependent protease
CJJ81176_0800		tpx	−6.21	0.03	−5.93	thiol peroxidase
CJJ81176_0703	*	uvrB	−6.72	0.01	−1.98	excinuclease ABC subunit B
CJJ81176_0879		–	−7.20	0.00	−2.05	phage integrase family
CJJ81176_1119	*	uvrD	−8.33	0.00	−1.72	ATP-dependent DNA helicase
CJJ81176_1220	*	radA	−6.91	0.00	−0.52	DNA repair protein RadA
CJJ81176_1279		recR	−7.25	0.00	−2.03	recombination protein
CJJ81176_1669		recA	−7.81	0.00	−2.02	recombinase A
**Motility and chemotaxis**						
CJJ81176_0080		flgD	−7.26	0.00	−2.66	flagellar basal body rod modification
CJJ81176_0097		fliY	−6.69	0.02	−6.72	flagellar motor switch protein
CJJ81176_0098		fliM	−7.20	0.00	−2.04	flagellar motor switch protein
CJJ81176_0099	*	fliA	−6.56	0.04	−1.86	flagellar biosynthesis sigma factor
CJJ81176_0100	*	flgV	−6.82	0.01	−0.70	newly identified flagellar protein
CJJ81176_0101		flhG	−8.32	0.00	−3.31	ParaA family ATPase
CJJ81176_0102	*	flhF	−7.12	0.00	0.38	flagellar biosynthesis regulator FlhF
CJJ81176_0226		fliI	−6.36	0.02	−4.02	flagellum-specific ATP synthase
CJJ81176_0357	*	flhB	−6.91	0.00	0.05	flagellar biosynthesis protein
CJJ81176_0358	*	motB	−6.36	0.02	−0.14	flagellar motor protein
CJJ81176_0359	*	motA	−7.18	0.00	−0.26	flagellar motor protein
CJJ81176_0376	*	fliO	−6.58	0.03	−0.56	flagellar protein
CJJ81176_0413	*	pflB	−7.39	0.00	0.44	TPR domain-containing protein
CJJ81176_0479	*	–	−7.02	0.00	−1.12	hypothetical protein
CJJ81176_0480	*	–	−7.21	0.00	0.29	hypothetical protein
CJJ81176_0481	*	–	−7.56	0.00	−0.39	hypothetical protein
CJJ81176_0696	*	rpoN	−6.18	0.04	−0.79	RNA polymerase factor sigma-54
CJJ81176_0814	*	flgR	−6.27	0.04	−0.17	sensor histidine kinase
CJJ81176_0837	*	fliP	−6.43	0.07	−0.43	flagellar biosynthesis protein
CJJ81176_0890	*	flhA	−7.81	0.00	−0.85	flagellar biosynthesis protein
CJJ81176_0974	*	–	−6.29	0.03	−1.78	flagellar secreted protein
CJJ81176_0996	*	–	−6.94	0.00	−0.27	Interact with FlgV and FliF
CJJ81176_1043	*	flgS	−6.74	0.01	0.05	DNA-binding response regulator
CJJ81176_1194	*	fliR	−7.29	0.00	−0.30	flagellar biosynthesis protein
CJJ81176_1459		flgK	−6.22	0.04	−2.30	flagellar hook-associated protein
CJJ81176_1550	*	pflA	−7.15	0.00	0.19	paralyzed flagella protein
CJJ81176_0309	*	cheW	−8.54	0.00	−1.18	purine-binding chemotaxis protein
CJJ81176_0310	*	cheA	−8.50	0.00	−0.80	chemotaxis protein
CJJ81176_0311	*	cheV	−6.24	0.04	−0.90	chemotaxis protein
CJJ81176_0387	*	cheX	−6.35	0.03	−0.91	chemotaxis phosphatase
CJJ81176_0930	*	cheR	−7.29	0.00	0.47	chemotaxis protein methyltransferase
CJJ81176_1193	*	chePep	−6.71	0.02	−1.52	a new family of chemotaxis regulator
**Cell wall, membrane, and envelope biogenesis**						
CJJ81176_0283	*	eptC	−7.60	0.00	1.24	lipid A phosphoethanolamine transferase
CJJ81176_0638		–	−7.94	0.00	−7.23	glycosyltransferase
CJJ81176_0673		–	−6.46	0.04	−2.07	lytic murein transglycosylase D
CJJ81176_0716		mraW	−6.24	0.03	−2.38	S-adenosyl-methyltransferase
CJJ81176_0859		–	−8.88	0.00	−2.19	soluble lytic murein transglycosylase
CJJ81176_0860	*	–	−7.55	0.00	−1.28	YGGT family protein
CJJ81176_1133	*	–	−6.99	0.00	−1.86	phosphatidylserine decarboxylase
CJJ81176_1138	*	pglF	−7.36	0.00	−0.62	general glycosylation pathway protein
CJJ81176_1142		pglA	−6.29	0.04	−3.78	general glycosylation pathway protein
CJJ81176_1145	*	pglI	−8.07	0.00	−0.72	general glycosylation pathway protein
CJJ81176_1152	*	–	−7.72	0.00	−0.60	glycosyltransferase
CJJ81176_1161	*	–	−7.87	0.00	−0.06	CMP-Neu5Ac synthetase
CJJ81176_1344	*	–	−7.25	0.00	−0.63	peptidoglycan peptidase 1
CJJ81176_1412		kpsS	−7.49	0.00	−2.37	capsule polysaccharide export protein
CJJ81176_1424		hddA	−7.04	0.00	−2.76	capsular biosynthesis sugar kinase
CJJ81176_1427	*	fcl	−6.52	0.04	−1.24	GDP-fucose synthetase
CJJ81176_1430		–	−7.40	0.00	−7.53	nucleotide-sugar epimerase-dehydratase
CJJ81176_1431		–	−9.10	0.00	−3.04	putative sugar transferase
CJJ81176_1434	*	–	−7.31	0.00	−1.49	putative sugar transferase
CJJ81176_1436		–	−7.19	0.00	−3.11	putative glycosyl transferase
CJJ81176_1666	*	cgpA	−7.38	0.00	−0.67	N-acetylgalactosamine-containing glycoproteins
**Miscellaneous function**						
CJJ81176_0136	*	–	−6.63	0.02	−0.39	ParB family chromosome partitioning protein
CJJ81176_0196	*	–	−6.28	0.04	−1.21	radical SAM domain-containing protein
CJJ81176_0653	*	hypC	−6.23	0.03	0.00	hydrogenase assembly chaperone
CJJ81176_0655	*	hypE	−7.03	0.00	−0.51	hydrogenase expression/formation protein
CJJ81176_0891	*	–	−7.14	0.00	−0.24	RrF2 family protein, putative
CJJ81176_1135	*	prmA	−6.74	0.01	−0.07	ribosomal protein L11 methyltransferase
CJJ81176_1232	*	asmA	−6.83	0.01	−1.49	predicted assembly protein
**Previously reported virulence factors**						
CJJ81176_0077	*	typA	−6.87	0.01	−0.85	GTP-binding protein
CJJ81176_0275	*	–	−7.92	0.00	−1.13	metal-dependent phosphohydrolase
CJJ81176_0295	*	–	−6.79	0.01	−0.28	SPFH domain-containing protein
CJJ81176_1048	*	mapA	−7.27	0.00	−1.31	Outer membrane protein
CJJ81176_1049	*	lepA	−7.42	0.00	−0.64	GTP-binding protein LepA
CJJ81176_1087	*	virK	−6.99	0.00	−1.78	hypothetical protein
CJJ81176_1225	*	yihY	−7.34	0.00	−1.79	virulence factor BrkB
**Function unknown**						
CJJ81176_0078	*	–	−6.29	0.03	−1.11	hypothetical protein
CJJ81176_0257		–	−7.06	0.00	−2.02	hypothetical protein
CJJ81176_0276	*	–	−8.78	0.00	−0.79	hypothetical protein
CJJ81176_0367		–	−7.85	0.00	−6.40	hypothetical protein
CJJ81176_0427	*	–	−7.14	0.00	−1.79	hypothetical protein
CJJ81176_0621	*	–	−7.70	0.00	−0.40	integral membrane protein
CJJ81176_0840	*	–	−6.24	0.04	−1.43	membrane protein
CJJ81176_0901	*	–	−6.84	0.01	−0.59	putative periplasmic protein
CJJ81176_1031	*	–	−6.27	0.04	−0.71	membrane protein
CJJ81176_1118		–	−6.63	0.02	−2.23	hypothetical protein
CJJ81176_1184	*	–	−6.45	0.04	−1.02	putative periplasmic protein
CJJ81176_1187		–	−6.99	0.00	−2.41	hypothetical protein
CJJ81176_1265	*	–	−6.77	0.01	−0.30	hypothetical protein
CJJ81176_1347	*	–	−6.70	0.02	−1.21	hypothetical protein
CJJ81176_1363	*	–	−6.41	0.04	0.02	hypothetical protein
CJJ81176_1389		–	−6.88	0.01	−2.91	DNA-binding protein

**Note**: Raw data are available in S3 Table. ABC, ATP-binding cassette; CMP, cytidine monophosphate; GDP, guanosine diphosphate; GTP; guanosine-5'-triphosphate; SAM, S-adenosyl methionine; TPR, tetratricopeptide repeat.

^**1**^ log2(fc) means the log2 value of the fold change (output/input). For raw data, see S1 and S3 Tables. All these genes have a q-value < 0.05.

* indicates that the corresponding gene mutant did not show a significant growth defect in rich medium (log 2 (fc) > −2.).

Our INSeq screen identified attenuating transposon mutations in most of the genes that previous studies have shown to be required for intestinal colonization of *C*. *jejuni* 81–176 [19,33,38–42], which demonstrates the comprehensive nature of our approach. Thus, *C*. *jejuni* 81–176 mutants harboring transposon insertions within genes involved in motility, lipooligosaccharide and capsule biosynthesis, or N- and O-protein glycosylation, as well as metabolic traits, were readily detected among the pool of mutants showing reduced colonization (Table 1 and S6 Table). Notably, however, several mutations in genes encoding cytolethal distending toxin components (CdtA, CdtB, and CdtC) and other previously described putative virulence factors involved in cell adhesion and invasion such as CiaB/C/I, CadF, FlpA, JlpA, PldA, or HtrA resulted in either no fitness cost or a level of attenuation significantly lower than many mutations affecting metabolic processes (see below) (S3 Table). These observations underscore the relative importance of metabolism in *C*. *jejuni* intestinal colonization. Our analysis also identified insertions in many genes that have not been previously linked to intestinal colonization. What follows is an analysis of these findings aided by the framework provided by the information obtained through studies of the requirements for *C*. *jejuni* growth under different in vitro culture conditions.

#### 1. Chemotaxis and motility

Chemotactic motility towards preferred nutrients, such as the amino acids Asp, Glu, and Ser, are essential for *C*. *jejuni* virulence and colonization [43,44]. Predictably, we identified several chemotaxis-associated genes that are required for mouse colonization (Table 1). Specifically, insertions in the genes encoding the chemotaxis-associated regulatory proteins CheA, CheV, and CheW and the accessory proteins CheR, CheX, and ChePep resulted in a significant decrease in the ability of *C*. *jejuni* to colonize the gut. Aspartate catabolism promotes *C*. *jejuni* infection [45,46], and the chemoreceptors Tlp1 and Tlp3 have been shown to interact with Asp [47,48]. Although *tlp3* appears to be a pseudogene in *C*. *jejuni* 81–176 [49], insertions within *tlp1* (CJJ81176_1498) resulted in a moderately significant attenuation (S6 Table). However, inactivation of other methyl-accepting Tlp proteins did not affect the ability of *C*. *jejuni* to colonize the murine intestine to the same extent (S7 Table). These results indicate that while chemotaxis is certainly required for colonization, there is likely to be significant redundancy in the function of the receptors that mediate the sensing of the different relevant chemoattractants.

Our analysis also revealed that insertions in any of 22 genes previously linked to motility resulted in a significant reduction in colonization (Table 1). These included the recently reported motility-associated genes *pflB* (CJJ81176_0413), *flgV* (CJJ81176_0100) [27], and CJJ81176_0481 [50]. The gene CJJ81176_0481 is organized in an operon with the downstream genes CJJ81176_0480 and CJJ81176_0479, which we found are also required for full motility and intestinal colonization (S7A and S7B Fig). Interestingly, insertional inactivation of more than 20 motility genes conferred an advantage during growth in liquid media (S2 Table, S7C Fig). These results imply that flagellar motility does not confer an advantage for *C*. *jejuni* growth in homogenous liquid medium and suggest that, under certain conditions, the metabolic cost of producing flagella may lead to the down-regulation of its expression. It has been demonstrated that the expression of several motility genes (e.g., *motA* and *flgRS*) in *C*. *jejuni* is controlled by phase-variable mechanisms [51–53]. Since insertional mutations in these phase-variable genes conferred a growth advantage in vitro, shutting down flagellar expression at some point during infection may have a beneficial effect for colonization. For example, it is possible that once *C*. *jejuni* has reached its preferred colonization sites, the mucus-filled intestinal crypts [54–56], motility might at least temporarily no longer confer an advantage. Instead, the phase-variable loss of motility might allow *C*. *jejuni* to more effectively utilize energy and carbon sources, thereby enhancing its ability to proliferate and persist in the mucus layer.

#### 2. Amino acid and peptide catabolism

**Fig 4:**
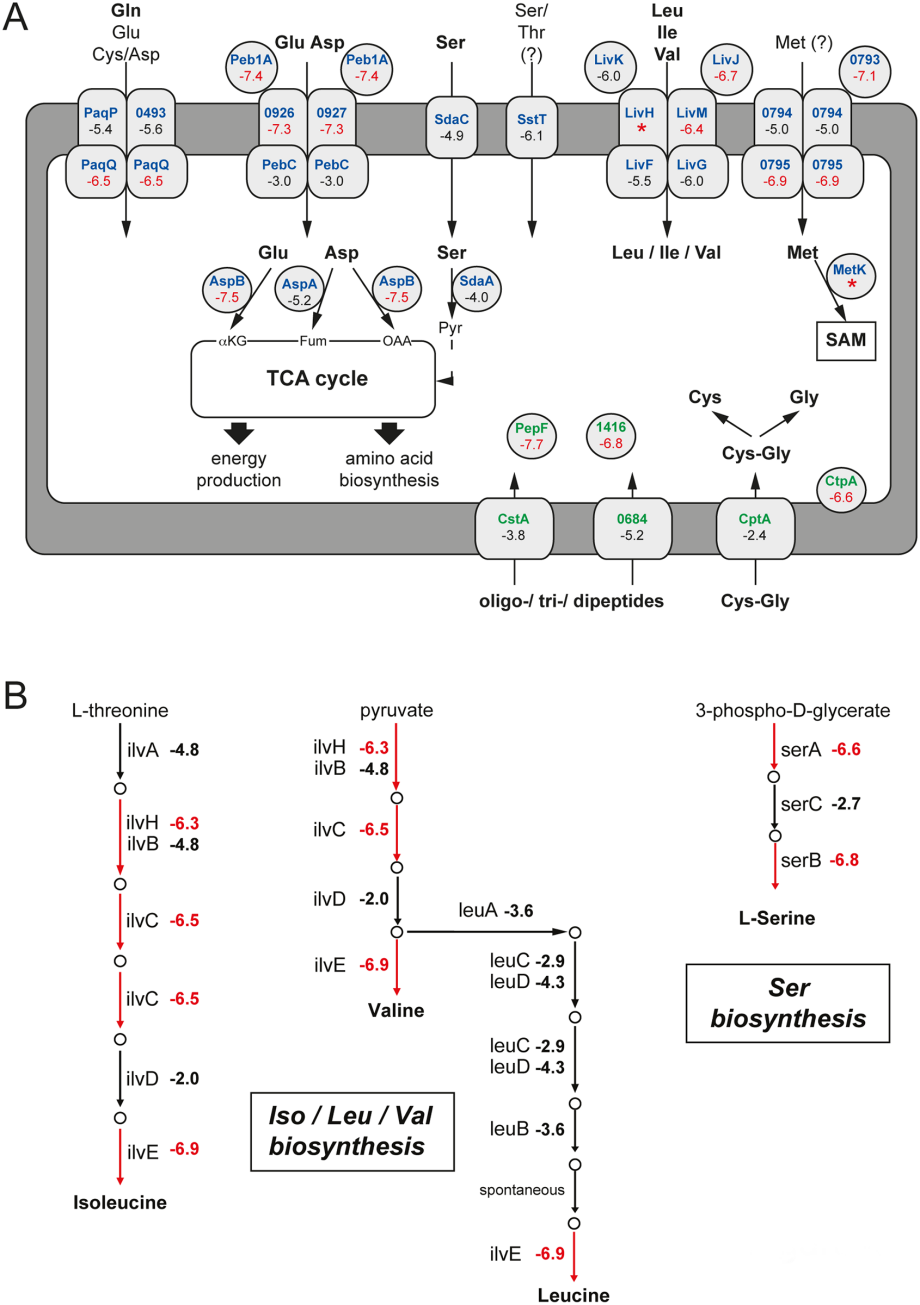
Role of amino acid and peptide metabolism in *C*. *jejuni* intestinal colonization. (A and B) Impact of the mutations in genes involved in the transport, metabolism (A), or biosynthesis (B) of amino acids and peptides that are important for *C*. *jejuni* colonization as determined by insertion sequencing (INSeq) analyses. The log_2_ (fold change [intestine/inoculum]) in the number of transposon insertions within the depicted *C*. *jejuni* genes are shown, and when depicted in red, the value indicates a statistically significant colonization defect. *: denotes genes showing a limited number of insertions within the library and no insertions within the pooled of mutants recovered from the intestine. The red arrows in panel (B) denote that the number of insertions within the gene involved in the indicated reaction was significantly reduced within the pooled of mutants recovered from the mouse intestine (relative to the inoculum). The log_2_ (fold change [intestine/inoculum]) depicted beside each gene is taken from S3 Table. Asp, asparagine; Cys, cysteine; Gln, glutamine; Glu, glutamic acid; Gly, glycine; Ile, isoleucine; Leu, leucine; Met, methionine; OAA, oxaloacetate; SAM, S-adenosyl methionine; Ser, serine; TCA, tricarboxylic acid; Thr, threonine; Val, valine.

Our INSeq analysis revealed that inactivation of *peb1A* (CJJ81176_0928), which participates in the utilization of Asp, Glu, Asn, and Gln [29,57], and its downstream genes (CJJ81176_0926, CJJ81176_0927) led to a significant decrease in colonization (Fig 4A). A similarly severe loss of colonization was observed for insertion mutants in *paqPQ* (Fig 4A), which encode a transporter that participates in the uptake of Gln and, to a lesser extent, Glu, Cys, and Asp [58]. In contrast to our previous study [59], mutants with defective Ser and Pro catabolism showed no colonization defect in this screen. This apparent discrepancy is likely due to the different animal models used in these studies and differences in the days after infection in which bacterial loads were examined (i.e., 4 days versus 5 weeks). Inactivation of the genes encoding the putative Ser/Thr (CJJ81176_1115) and Met (CJJ81176_0793–0795) transporters also resulted in a pronounced colonization defect, suggesting that these predicted transporters are functional in *C*. *jejuni* (Fig 4A). Indeed, as mentioned in our metabolic footprint analysis, we detected a significant reduction of Thr and Met after *C*. *jejuni* growth in casein hydrolysate medium, confirming that these amino acids can be taken up by *C*. *jejuni* (S3 Fig). Transposon insertions within the genes *livFGMKJ*, which encode a high-affinity branched-chain amino acid ATP-binding cassette (ABC) transporter, resulted in a drastic reduction in *C*. *jejuni* intestinal colonization (Fig 4A and S3 Table). Similarly, inactivation of *livK* and *livJ* resulted in a severe colonization defect in a chicken model of infection [33]. As Leu, Ile, and Val are not catabolized by *C*. *jejuni*, these findings suggest that the acquisition of branched-chain amino acids must be crucial for protein synthesis during *C*. *jejuni* infection.

The limited number of metabolizable amino acids would appear to be a limitation in the ability of nonglycolytic *C*. *jejuni* to acquire nutrients. However, our INSeq analysis revealed that the catabolism of peptides, presumably released from the diet or from host-derived proteins by the host digestive enzymes and/or the intestinal microbiota [60], may be important for *C*. *jejuni* colonization. Although transposon insertions in several genes encoding peptidases (CJJ81176_0166, CJJ81176_0488, CJJ81176_0746, CJJ81176_0826, CJJ81176_1249, and CJJ81176_1291) resulted in no or moderate intestinal colonization defects, presumably due to redundancy in their activities, inactivation of the cytoplasmic zinc metalloendopeptidase PepF (CJJ81176_1117), the putative family C26 endopeptidase CJJ81176_1416, or the carboxyl-terminal protease CtpA (CJJ81176_0539) resulted in a severe colonization defect (Fig 4A, S3 Table). These results are consistent with previous in vitro studies indicating that *C*. *jejuni* can utilize dipeptides and tripeptides as a source of amino acids such as Glu and Cys that can serve as an energy/carbon source or can be used in protein biosynthesis [61].

#### 3. Amino acid biosynthesis

Despite the fact that *C*. *jejuni* 81–176 is able to take up most amino acids under in vitro growth conditions, our INSeq analysis revealed that its ability to synthesize several amino acids de novo is central for intestinal colonization (S8 Fig and S3 Table). In particular, the de novo synthesis of the branched-chain amino acids Leu, Ile, and Val is essential to colonize mice since insertional mutations in *ilvC* (CJJ81176_0660) and *ilvE* (CJJ81176_0296) and *invH* (CJJ81176_0603) were recovered in significantly lower number from the mouse intestine (Fig 4B). The requirement for both the acquisition and the synthesis of branched-chain amino acids implies that there is a high demand for these amino acids to sustain normal protein synthesis since branched-chain amino acids cannot be used as carbon source. Consistent with this hypothesis, branched-chain amino acids constitute 24.5% of the predicted *C*. *jejuni* 81–176 proteome (S9 Fig).

Transposon insertions in the Ser biosynthesis genes *serAB* resulted in a significant colonization defect (Fig 4B). This observation is surprising because *C*. *jejuni* can efficiently take up and utilize Ser as growth substrate. Moreover, the *serAB* mutants showed no growth defects when cultivated on nutrient-rich blood agar plates (S1 Table). Although Ser is abundantly present in animal feces [62], a restricted Ser supply at the *C*. *jejuni* colonization site may be responsible for the colonization defect observed in the *serAB* mutants. Furthermore, we found that in Ser-rich (2 mM) defined medium with an additional growth substrate, the added Ser seems to be insufficient for de novo protein synthesis in a *C*. *jejuni serA* mutant (S10A Fig). Isotopologue profiling experiments revealed that the imported free Ser is efficiently used as a carbon and energy source (Fig 2), thus potentially limiting the availability of free Ser for protein synthesis. Accordingly, the growth defect of the *C*. *jejuni serA* mutant could be overcome by introducing an additional mutation in the serine dehydratase *sdaA* gene, which prevents the degradation of the imported Ser (S10A Fig), or by increasing the amounts of free Ser (S10B and S10C Fig). Together, our results suggest that during colonization the imported Ser is efficiently catabolized by *C*. *jejuni* to fuel the TCA cycle, thereby limiting its intracellular accumulation and subsequent incorporation into newly synthesized proteins. In contrast, peptide-bound Ser in the form of the dipeptide Leu-Ser promoted the in vitro growth of the *C*. *jejuni* serA mutant more efficiently than free Ser (S10D Fig). This observation further stresses the importance of peptide utilization for the proliferation of *C*. *jejuni*. Further in vivo studies will be required to clarify the potentially different intracellular fates of free and peptide-bound Ser in this pathogen.

*C*. *jejuni* 81–176 does not seem to depend on the de novo synthesis of Met during mouse colonization since no insertional mutants of genes involved in Met biosynthesis (*metAXCE*) showed a significant colonization defect (S11 Fig). However, the INSeq analysis revealed that a putative Met transporter encoded by *metNIQ* is required for intestinal colonization, suggesting an important role for Met in *C*. *jejuni* physiology in the gut (Fig 4A, Table 1). In addition to protein synthesis, Met is crucial for bacterial physiology as a precursor of S-adenosyl methionine (SAM), a major methyl donor required for the methylation of DNA, RNA, proteins, and lipids through the activity of SAM-dependent methyltransferases [63]. We did not identify any gene encoding an obvious homolog of a SAM transporter in the *C*. *jejuni* 81–176 genome. However, the *C*. *jejuni* 81–176 genome harbors a *metK* homologue, which encodes a SAM synthetase that converts Met directly to SAM. We detected very few insertions within *metK* in our transposon library (7 reads per 10^6^ total reads, all located at the same site), and none of the *metK* insertional mutants were recovered from the mouse intestine or after in vitro growth, which indicates a critical role for SAM in *C*. *jejuni* physiology (S1 and S3 Tables). Therefore, we hypothesize that Met transport is crucial for *C*. *jejuni* growth in vitro and in vivo as a precursor for SAM biosynthesis.

#### 4. The TCA cycle and acetate switch

**Fig 5:**
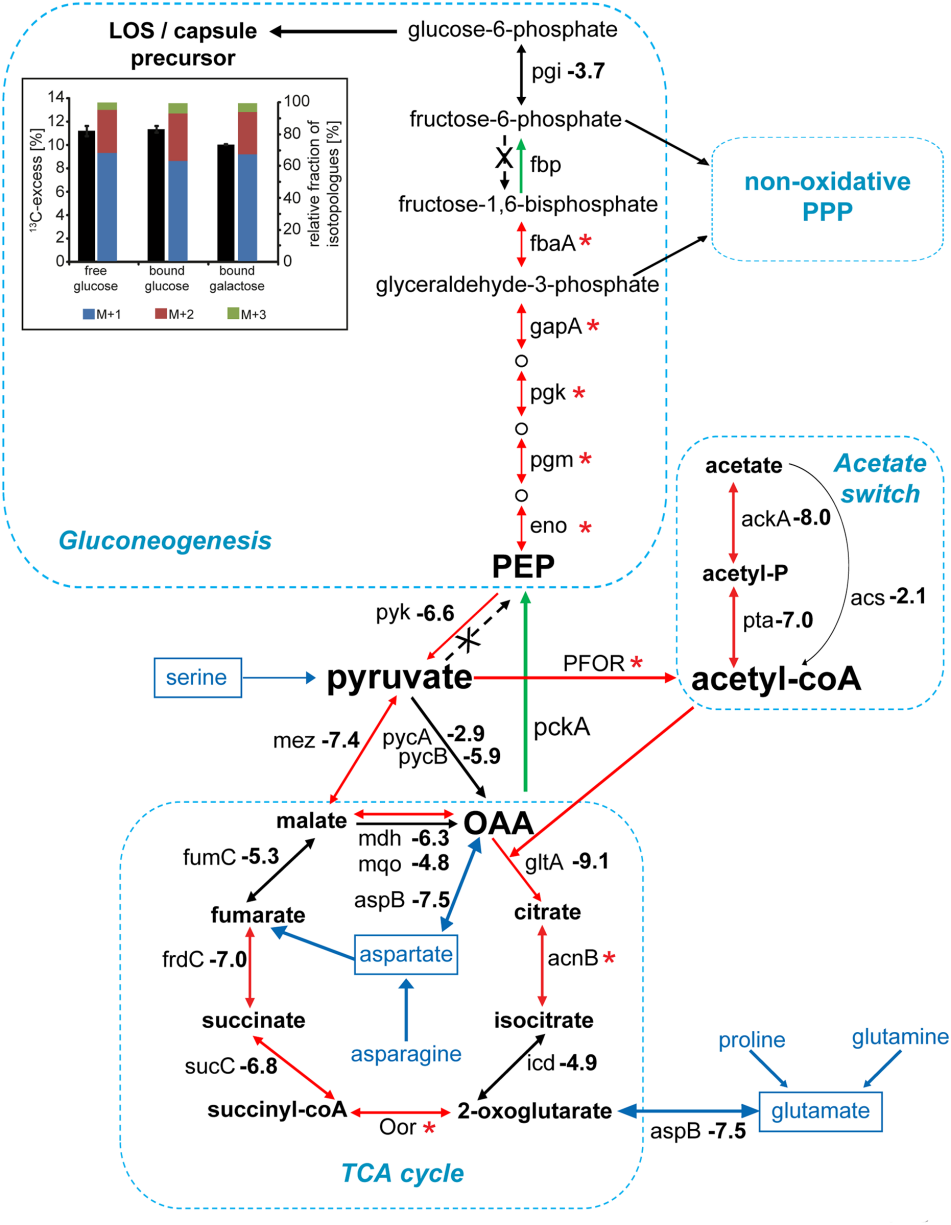
Impact of central carbon metabolism during *C*. *jejuni* intestinal colonization. The log_2_ (fold change [intestine/inoculum]) in the number of transposon insertions within *C*. *jejuni* genes encoding enzymes in the tricarboxylic acid (TCA) cycle, gluconeogenesis, and the acetate switch pathways are shown and are derived from the raw data in S3 Table. Values below −6.2 indicate mutations that led to a statistically significant colonization defect. *: denotes genes showing a limited number of insertions within the library and no insertions within the pooled of mutants recovered from the intestine. The red arrows denote that the number of insertions within the gene involved in the indicated reaction was significantly reduced within the pool of mutants recovered from the mouse intestine (relative to the inoculum). Green arrows indicate that the enzyme that catalyzes the corresponding reaction does not have an insertional mutant in our mutant library. Enzymes not encoded in the *C*. *jejuni* genome are indicated with an “X.” The inset depicts the overall ^13^C-excess and relative fractions of ^13^C-labeled isotopologues in free and bound glucose or galactose (as indicated) derived from *C*. *jejuni* 81–176 cell surface carbohydrates after cultivation in Dulbecco’s Modified Eagle Medium (DMEM) with [3-^13^C_1_]Ser. The colored boxes indicate the relative contributions (%] of isotopologues with 1, 2, and 3 ^13^C-atoms indicated as M+1, M+2, and M+3, respectively. Numbers are the means ± standard deviation (SD) of 6 measurements (see S11 Table). Acetyl-CoA, acetyl coenzyme A; LOS, lipooligosaccharide; OAA, oxaloacetate; PEP, phosphoenolpyruvic acid; PPP, pentose phosphate pathway.

The INSeq analyses revealed that the central metabolism enzymes GltA, SucCD, and FrdABC, which catalyze various TCA cycle reactions, are critical for *C*. *jejuni* growth in defined medium and crucial for mouse colonization (Fig 5 and Table 1). Strikingly, the *gltA*, *sucCD*, and *frdAC* mutants showed no comparable growth defects when cultivated on nutrient-rich blood agar plates (Table 1), suggesting that in the intestine, *C*. *jejuni* must reside in a nutritionally restricted environment. Similarly, in agreement with previous reports [46,45], inactivation of *aspA* or *aspB*, whose products fuel the TCA cycle by catalyzing the catabolism of Asp and Glu, did not hinder the growth of *C*. *jejuni* on blood agar plates but led to a severe reduction in intestinal colonization. These observations indicate that Asp and Glu are among the most important carbon sources for *C*. *jejuni* in the gastrointestinal tract (Fig 4). Isotopologue profiling experiments with [5-^13^C_1_]Glu confirmed the central role of glutamate in the anabolism of *C*. *jejuni*, fueling gluconeogenesis and the nonoxidative PPP as demonstrated by the detection of ^13^C-labeled Ser, Gly, and His (S12 and S13 Figs). The anabolism and energy production through Glu catabolism require the AspB-catalyzed deamination of Glu to 2-oxoglutarate, which is then converted to malate and oxaloacetate (Fig 5). Subsequently, the decarboxylation of malate catalyzed by Mez (CJJ81176_1304) or the conversion of oxaloacetate through the concerted activities of PckA (CJJ81176_0939) and Pyk (CJJ81176_0415) generates Pyr, the precursor for many anabolic reactions (Fig 5). The INSeq analysis revealed that Mdh, Mez, and Pyk play a crucial role during *C*. *jejuni* colonization of the mouse intestine, although they are less important for the in vitro growth in rich media (Fig 5 and Table 1). Thus, our findings place Pyr at the center of the carbon flow of *C*. *jejuni* during gut colonization.

As an energy source, Pyr can be degraded by *C*. *jejuni* to CO_2_ through the TCA cycle or fermented to acetate (Fig 5). In both cases, the first catabolic intermediate is acetyl-CoA, which is generated through the pyruvate ferredoxin/flavodoxin oxidoreductase (PFOR) (CJJ81176_1469) [64]. We recovered very few transposon insertions within PFOR in our original *C*. *jejuni* mutant library and none from infected mice (S1 and S3 Tables), indicating that the production of acetyl-CoA is vital for both in vitro growth and intestinal colonization. Acetyl-CoA can be converted to acetyl phosphate and subsequently to acetate by the concerted activities of the phosphate acetyltransferase Pta (CJJ81176_0711) and the acetate kinase AckA (CJJ81176_0712) [65]. This substrate-level phosphorylation reaction allows *C*. *jejuni* to generate ATP to energize its anabolism in an environment that lacks sufficient amounts of oxygen or alternative electron acceptors for oxidative phosphorylation (Fig 5). Alternatively, Pta, AckA, and also the acetyl-CoA synthetase Acs (CJJ81176_1522) can catalyze the reverse reaction generating acetyl-CoA from acetate. This so-called “acetate switch” occurs in vitro during the stationary growth phase of *C*. *jejuni* [65]. While mutants with an inactivated *acs* gene showed no colonization defect, transposon insertions in both *pta* and *ackA* were recovered in significantly reduced numbers from infected animals, indicating that this pathway is essential for mouse colonization (Table 1). This observation was confirmed in infection experiments in which drastically lower numbers of a *C*. *jejuni* Δ*ackA* mutant were recovered from mice when competed against the wild-type strain (S14 Fig). As the *C*. *jejuni pta* and *ackA* transposon insertion mutants exhibited no in vitro growth defect on blood agar plates, our infection experiments suggest that ATP generation through substrate-level phosphorylation during the conversion of acetyl-CoA to acetate and an operational “acetate switch” are required for mouse colonization. *C*. *jejuni* can utilize acetate in vitro [65], and our INSeq analysis suggests that *C*. *jejuni* might take advantage of the acetate produced by the intestinal microbiota to fuel its TCA cycle and balance its anabolic metabolism through the concerted activities of Pta and AckA. Additionally, the intermediate acetyl phosphate is involved in gene regulation as a potential phosphorylation donor [66], which suggests that the regulatory effect of acetyl phosphate might also contribute to intestinal colonization.

#### 5. Gluconeogenesis and the nonoxidative pentose phosphate pathway

*C*. *jejuni* does not catabolize glucose, as it lacks the genes encoding the enzymes for the oxidative pentose phosphate and Entner-Doudoroff pathways as well as the gene for the glycolytic enzyme phosphofructokinase (Pfk) of the Emden-Meyerhof-Parnas (EMP) pathway [67]. Nevertheless, our INSeq analyses showed that *C*. *jejuni* depends on the gluconeogenic activity of the EMP pathway for in vitro growth in rich and defined media as well as for the colonization of the mouse intestine. Accordingly, insertions in genes within the EMP pathway were either missing (*fbp*) or present in very low numbers (*fbaA*, *gapA*, *pgk*, *pgm*, and *eno*) in our mutant library, and none of these mutants were recovered from the mouse intestine (Fig 5 and S1 and S3 Tables). These results suggest that gluconeogenesis is the only mechanism by which *C*. *jejuni* can obtain glucose and other EMP-pathway intermediates that are precursors for the nonoxidative PPP. Consequently, *C*. *jejuni* utilizes gluconeogenesis for the synthesis of carbohydrates, which are incorporated into essential virulence factors such as its lipooligosaccharide (LOS) and capsule (CPS) as well as the N-linked heptasaccharides of glycoproteins that play essential roles for *C*. *jejuni* proliferation in mice. Isotopologue profiling experiments with [3-^13^C_1_]Ser or [5-^13^C_1_]Glu demonstrated the existence of a reverse EMP pathway in *C*. *jejuni* since we detected ^13^C-labeled glucose and galactose derived from the LOS- and CPS-containing surface glycan (Fig 5, S13 Fig). However, *C*. *jejuni* cannot generate glucose from [3-^13^C_1_]Ser through its direct conversion to Pyr and phosphoenolpyruvic acid (PEP) because of the lack of a PEP synthetase. Consequently, Pyr, derived from Ser, must enter the EMP pathway either through the PFOR-catalyzed decarboxylation as acetyl-CoA in the TCA cycle or through its direct carboxylation by the pyruvate carboxylase Pyc (PycB: CJJ81176_0940; PycA: CJJ81176_0956) to generate the TCA cycle intermediate OAA. *C*. *jejuni* encodes a PEP carboxykinase (PckA; CJJ81176_0939) that converts OAA into PEP, which fuels the reverse EMP pathway for the synthesis of cell surface carbohydrates, or serves as substrate for other anaplerotic reactions. Indeed, our in vitro and in vivo INSeq analyses indicate that *pckA* is an essential gene (Fig 5), which is consistent with previous observations [68]. Moreover, our analysis revealed that the nonoxidative PPP enzymes ribose 5-phosphate isomerase B (RpiB) and ribulose-phosphate 3-epimerase (Rpe) are essential for the growth of *C*. *jejuni* in vitro and in vivo (S1 and S3 Tables, S15 Fig). The nonoxidative PPP facilitates the generation of aromatic amino acids and His, as well as purines and pyrimidines from EMP pathway intermediates. Insertion mutants within genes encoding enzymes of the purine and pyrimidine biosynthesis pathways were recovered in significantly reduced numbers from infected mice (Table 1, S3 Table). Inactivation of the *purS*, *purQ*, *purL*, *carB*, *pyrB*, or *pyrF* genes resulted in mutants with normal growth phenotypes in rich medium but significant colonization defects in mice (S1 and S3 Tables, S16 Fig), suggesting the presence of yet unidentified purine and pyrimidine transporters in *C*. *jejuni* and indicating that both nucleotides may be in short supply within the mouse intestinal tract. Together, our study demonstrates a central role for the gluconeogenesis and PPP pathways of *C*. *jejuni* in intestinal colonization.

#### 6. CO_2_ utilization

**Fig 6:**
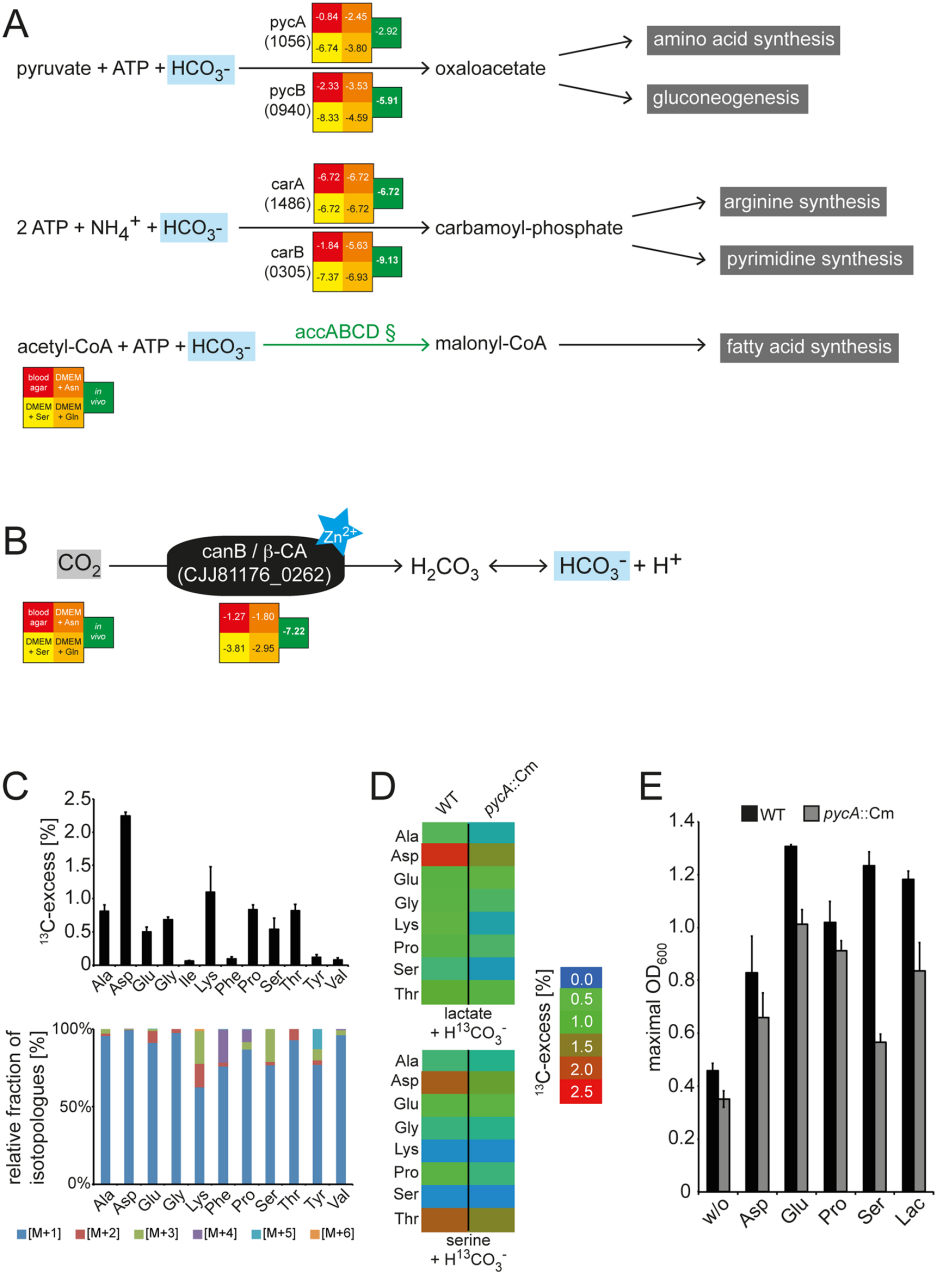
The contribution of CO_2_ metabolism to *C*. *jejuni* intestinal colonization. Illustrated are metabolic reactions in *C*. *jejuni* that utilize bicarbonate (H_2_CO_3_^-^) (A) and the carbonic anhydrase CanB-catalyzed reaction that generates bicarbonate from CO_2_ (B). Numbers indicate the log2 (fold change [intestine/inoculum]) in the number of insertions in the indicated genes and are derived from the raw data in S3 Table. Values below −6.2 indicate mutations that led to a statistically significant colonization defect. Green arrows indicate mutations that led to a statistically significant colonization defect. (C) Incorporation of CO_2_ into amino acids after *C*. *jejuni* 81–176 cultivation in Dulbecco’s Modified Eagle Medium (DMEM) supplemented with H^13^CO_3_^-^. Shown are the overall ^13^C-excess (%) (upper panel) and relative fractions of ^13^C-labeled isotopologues (lower panel) in protein-derived amino acids of *C*. *jejuni* 81–176 cultivated in DMEM supplemented with 44 mM ^13^C-labeled hydrogen carbonate. The colored boxes indicate the relative contributions (%) of isotopologues with 1, 2, 3, 4, 5, and 6 ^13^C-atoms (M+1, M+2, M+3, M+4, M+5, and M+6). Numbers are the means ± standard deviation (SD) of 6 measurements (see S11 Table). (D) Heat map for the overall ^13^C-excess of labeled amino acids in *C*. *jejuni* 81–176 wild-type and the respective *pycA*::*Cm* mutant strain after growth with [^13^C]bicarbonate and 20 mM lactate (upper panel) or 20 mM Ser (lower panel) as carbon and energy sources. The values of the color map depict the mean of 2 biological experiments measured in triplicate (see S11 Table). (E) Growth analysis of the *C*. *jejuni* 81–176 *pycA* mutant (grey column) compared to the wild type (black column) when cultivated in DMEM supplemented with 20 mM of different carbon and energy sources. Values represent the mean values ± SD of 3 independent experiments (see S12 Table). Ala, alanine; Asp, asparagine; Glu, glutamic acid; Gly, glycine; Ile, isoleucine; Lac, lactate; Leu, leucine; Lys, lysine; Phe, phenylalanine; Pro, proline; Ser, serine; Thr, threonine; Tyr, tyrosine; Val, valine; w/o, without; WT, wild type.

Our findings indicate that the link between the TCA cycle and the EMP pathway via the PEP-Pyr-OAA-malate node is critical for *C*. *jejuni* to adjust the carbon fluxes to its energetic and anabolic needs both for its growth in vitro and during intestinal colonization (Fig 5). Maintenance of the intracellular OAA pools is indeed essential for growth and can be achieved by fueling the TCA cycle through the catabolism of Asp, Glu, Pro, and Ser or by carboxylating Pyr through the activity of Pyc [68]. The CO_2_ required for this reaction might be generated intracellularly, for example, through the decarboxylation of TCA cycle intermediates or during the biosynthesis of fatty acids (S17 Fig). In the intestine, however, *C*. *jejuni* may be able to acquire the CO_2_ generated as a catabolic end product from the fermentation activity of the intestinal microbiota or directly from the host [69]. Various CO_2_-incorporating metabolic reactions require hydrogen carbonate (HCO_3_^-^) instead of gaseous CO_2_ (Fig 6A), and the hydration of CO_2_ to HCO_3_^-^ depends in *C*. *jejuni* on the carbonic anhydrase CanB (CJJ81176_0262) (Fig 6B), which promotes its growth in vitro [70]. Besides facilitating the carboxylation reactions, the generated HCO_3_^-^ might be required for maintaining the intracellular pH homeostasis [71]. In addition, our INSeq analysis suggests that metabolic reactions with hydrogen carbonate are crucial for *C*. *jejuni* growth in the mouse intestine as revealed by the attenuated phenotypes observed for insertions within the genes *carAB* (CJJ81176_1486 / CJJ81176_0305), which encode a carbamoyl phosphate synthase (Fig 6A, S3 Table). Cultivation of *C*. *jejuni* in medium with ^13^C-labeled hydrogen carbonate (HCO_3_^-^) demonstrated CO_2_ incorporation into protein-derived amino acids (Fig 6C). In particular, the ^13^C-labeling of Asp and the amino acids Lys and Thr, which derive from Asp, illustrates the Pyc-mediated carboxylation of Pyr to OAA, which is subsequently converted to Asp by amination. Inactivation of *pycA* resulted in a slight but significant reduction in ^13^C-labeling of the detected amino acids, mainly Asp (Fig 6D), demonstrating the redundancy in HCO_3_^-^ incorporation by the carboxylating enzymes in *C*. *jejuni* (Fig 6A). The inability of the *C*. *jejuni pycA* mutant to metabolize HCO_3_^-^ led to a reduction in its in vitro growth, especially when cultivated with Ser and lactate as sole energy source (Fig 6E), similar to what has been previously described for the *canB* mutant [70]. However, the *pyc* mutants showed no significant colonization defect in mice (Fig 6A), indicating that the amount of OAA, generated through the catabolism of growth substrates that fuel the TCA cycle, is sufficient for its gluconeogenic requirements. In contrast, the *canB* (CJJ81176_0262) mutant was highly attenuated in our infection experiments (Fig 6B and Table 1), demonstrating that the ability of the capnophilic *C*. *jejuni* to utilize CO_2_ for its carboxylation reactions is essential for its ability to colonize the intestine.

#### 7. Transient metal cofactors

**Fig 7:**
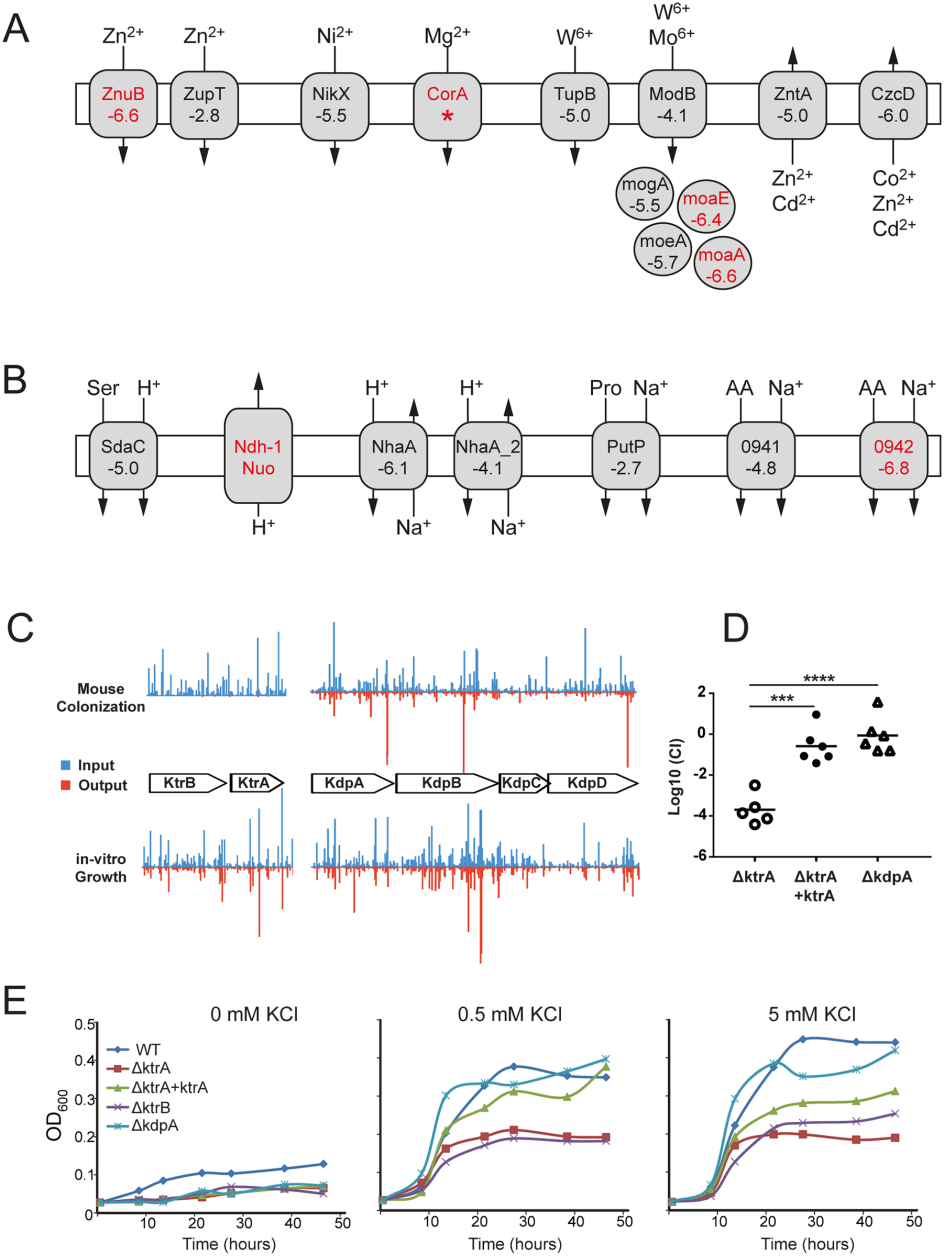
Ion homeostasis in *C*. *jejuni* intestinal colonization. Overview of insertion sequencing (INSeq) data for putative transporters in *C*. *jejuni* 81–176 mediating the transport of transition metals (A) and facilitating the homeostasis of protons and sodium or the uptake of solutes by cotransport (B). (C) Comparison of the relative contribution of 2 predicted potassium transport systems (Ktr and Kdp) to *C*. *jejuni* mouse colonization. Blue and red bars indicate the normalized read number of each insertion site within the open reading frames (ORFs) in the input and output pool, respectively. (D) Role of the Ktr and Kdp potassium transport system in mouse colonization. Mice were inoculated with an equal number of wild-type *C*. *jejuni* and the indicated mutant or complemented mutant strains via oral gavage (*n* = 5 or 7). Competitive indices (CIs) were calculated as the ratio of the colony-forming units (CFUs) of the mutant over the CFUs of the wild-type strain recovered from the ceca of infected mice (see S14 Table). Significance was determined by the unpaired *t* test. ***: *p* = 0.001; ****: *p* < 0.0001. (E) Growth of *C*. *jejuni ktr* and *kdp* mutants in defined rich medium supplemented with various K^+^ concentrations, as indicated. All strains were inoculated to culture medium at an OD_600_ of 0.02, and the cell density of the cultures was measured at the indicated times over a 46-hour period. Pro, proline; Ser, serine.

Maintaining the homeostasis of ions is crucial for the viability of bacteria. The micronutrients copper (Cu), iron (Fe), manganese (Mn), magnesium (Mg), molybdenum (Mo), nickel (Ni), tungsten (W), and zinc (Zn) are required as cofactors for the activities of various enzymes. Although traces of these transient metals are essential for bacterial growth, elevated ion concentrations have toxic effects [72,73]. Animal hosts use transient metals as part of their nutritional immunity to fight invading pathogens by either restricting the access to free ions like Fe, Mg, and Mn or generating an environment with elevated concentrations of other metals such as Cu, which is toxic for bacteria. Consequently, pathogens require sufficient ion import and efflux mechanisms to overcome these host defense mechanisms and to balance their ion homeostasis [74]. Consistent with the importance of Mg^2+^ in *C*. *jejuni* physiology, we found very few insertions within the magnesium transporter gene *corA* (CJJ81176_0749) in our transposon mutant library. Furthermore, as previously shown in chickens [39], we recovered very few insertions within the *znuABC* operon, which encodes a zinc transporter, from the pool of mutants obtained from the mouse intestine (Table 1). In contrast, inactivation of the putative zinc transporter ZupT (CJJ81176_0290) did not hinder the intestinal colonization of *C*. *jejuni* (Fig 7A), suggesting a less prominent role for this transporter. *C*. *jejuni* utilizes molybdate as cofactors for respiratory enzymes like the nitrate reductase and sulfite oxidase [75]. Insertions within the genes encoding the molybdenum cofactor biosynthesis gene *mogA* (CJJ81176_0748) and the molybdopterin biosynthesis genes MoaA (CJJ81176_0197), MoaE (CJJ81176_1510), and MoeA (CJJ81176_0873) were greatly reduced in the mouse intestine, indicating that molybdenum plays an important role during *C*. *jejuni* intestinal colonization (Fig 7A, S3 Table).

Unexpectedly, transposon insertions within genes involved in iron uptake systems did not have a major impact on the colonization capacity of *C*. *jejuni* 81–176 (S3 Table). *C*. *jejuni* 81–176 encodes at least 5 transporter systems related to iron uptake, such as CeuBCDE (CJJ81176_1351–1354), FeoAB (CJJ81176_1395–1397), ChuABCD (CJJ81176_1601–1604), CfbpABC (CJJ81176_0209–0211), and Ftr1/P19 (CJJ81176_1649–1650), and these results suggest a potential redundancy in the mechanisms utilized by *C*. *jejuni* to acquire iron.

Elevated Cu concentrations are used as a host defense mechanism against pathogenic microorganisms by facilitating the generation of reactive oxygen and nitrogen species. The periplasmic copper oxidase CeuO (CJJ81176_1508) and the copper exporter CopA (CJJ81176_1176) mediate the copper tolerance of *C*. *jejuni* in vitro [76]. Surprisingly, however, transposon insertions within *ceuO* or *copA* did not result in significant colonization defects (Fig 7A, S3 Table). Instead, we found that insertions within genes encoding a second predicted copper oxidase, CJJ81176_1230, significantly affected the ability of *C*. *jejuni* to colonize the mouse intestine. A similar colonization defect was observed in insertions within *czcD*, which encodes a putative cobalt-zinc-cadmium efflux system protein (CJJ81176_1178) (S3 Table). These observations suggest that *C*. *jejuni* may be able to neutralize the Cu-mediated host defense mechanism through the activity of these 2 proteins.

#### 8. Sodium, proton, and potassium transporters

Protons (H^+^) and sodium (Na^+^) ions participate in the regulation of the cellular pH in bacteria [77], whereas potassium ions (K^+^) have a central role in sustaining turgor pressure [78]. *C*. *jejuni* encodes 2 putative homologs of transport proteins of the Na^+^/H^+^ antiporter family (S3 Table), which play a key role in pH and Na^+^ homeostasis [77]. Inactivation of one of them, the putative NhaA homolog CJJ81176_1645, led to a severe intestinal colonization defect with no in vitro phenotype (Fig 7B and S3 Table). The significance of this observation, however, is uncertain since the actual substrate specificity of this putative transporter is unknown but suggests an important role in intracellular pH regulation in intestinal colonization.

Protons and sodium ions also participate in the transport of growth substrates through the activity of various symporters. In addition to the sodium and proton symporters PutP and SdaC (Fig 7B), which are utilized in the transport of Pro and Ser, *C*. *jejuni* encodes other putative sodium symporters such as CJJ81176_0052, CJJ81176_0629, CJJ81176_0941, and CJJ81176_0942. Interestingly, insertion mutants within CJJ81176_0942 were recovered in drastically reduced numbers from the mouse intestine, although they did not incur a fitness cost when grown in vitro (Table 1). The interpretation of this observation, however, awaits the identification of the actual substrate of this putative symporter.

To ensure the maintenance of the potassium-dependent turgor pressure, bacteria are often equipped with several K^+^ import systems, such as the low-affinity but constitutive Trk and Kup transporters, the Na^+^-dependent Ktr importer, or the high-affinity and inducible Kdp transport system [78,79]. *C*. *jejuni* 81–176 encodes homologues of the Kdp and Ktr systems (S8 Table). Insertions in the *ktrAB* and *kdpABCD* genes resulted in no significant fitness cost after growth in vitro (Fig 7C and S1 Table). Strikingly, however, the number of insertions within the *ktrAB* locus was drastically reduced in the pool of mutants recovered from mice (Fig 7C and S3 Table). In contrast, the *kdpABCD* mutants were not affected in their ability to colonize the mouse intestine. To further clarify the role of the K^+^ transport systems in *C*. *jejuni* 81–176, we constructed strains carrying mutations in the *ktr* or *kdp* transport systems and examined their ability to colonize the mouse intestine in coinfection experiments with the wild-type strain (Fig 7D). Similar to what we observed in the INSeq analysis, we found that the *C*. *jejuni* Δ*ktrA* mutant was drastically impaired in its ability to colonize the mouse intestine and that the colonization defect was fully reversed upon reintroduction of a wild-type copy of *ktrA*. In contrast, we found that the Δ*kdpA* mutant was able to colonize the mouse intestine at levels equivalent to those of the wild type. We also tested the growth of *ktr* and *kdp* mutants in defined medium in the presence or absence of KCl (Fig 7E). In the absence of KCl supplementation, only the wild-type strain showed measurable growth, although at reduced levels. In the presence of KCl, the wild type and Δ*kdpA* mutant strains were able to grow at equivalent levels, while the growth of the Δ*ktrA* and Δ*ktrB* mutant strain was significantly impaired. Growth of the Δ*ktrA* mutant strains was restored upon reintroduction of a wild-type copy of the *ktrA* gene. These results indicate that the KtrAB K^+^ transport system plays a central role in *C*. *jejuni* physiology and mouse colonization.

### Concluding remarks

It has become increasingly clear that the ability to secure nutrients is a key determinant of bacterial pathogenesis [14,16,80]. Our in vivo INSeq analysis has provided evidence that during the infection process *C*. *jejuni* relies more on specific metabolic adaptations than on specific virulence factors targeting host processes. In contrast to other intestinal pathogens, like *Salmonella* or pathogenic *Escherichia coli* [81–83], *C*. *jejuni* utilizes a limited number of carbon and energy sources. However, since low *C*. *jejuni* infectious doses are sufficient to cause disease [84,85], such metabolic restrictions do not seem to impede the ability of *C*. *jejuni* to overcome the microbiota-mediated colonization resistance. In fact, it is possible that *C*. *jejuni* may rely on the microbiota to provide essential nutrients to fuel its metabolism. We find that *C*. *jejuni* can utilize potential catabolic end products of the intestinal microbiota such as acetate or CO_2_-derived hydrogen carbonate and especially free amino acids and di-/ or oligopeptides, which are released from dietary or endogenous proteins. Although free amino acids and dipeptides are prominent in the mucus layer of the small intestine, our study suggests that the concentration of some amino acids may not be sufficient for *C*. *jejuni* since auxotrophic mutants unable to synthesize branched-chain and aromatic amino acids or Ser exhibit a colonization defect. We show that *C*. *jejuni* overcomes such substrate restriction through an active TCA cycle, gluconeogenesis, and nonoxidative PPP, which together facilitate its anabolic capacity and are crucial for its in vitro and in vivo growth. *C*. *jejuni* preferentially colonizes the mucus of the intestinal crypts [54–56], where it is spatially segregated from the luminal microbiota [86,87]. This spatial separation may allow *C*. *jejuni* to thrive despite its limited metabolic capacity. Indeed, while other enteropathogenic bacteria like *Salmonella* exhibit redundant catabolic pathways that allow the exploitation of multiple nutrients [88], our analyses clearly showed that the metabolic network of *C*. *jejuni* lacks comparable redundancy and harbors various bottlenecks, as it depends on the gluconeogenic activity of the EMP pathway and the nonoxidative PPP. These metabolic bottlenecks may allow the development of novel strategies to prevent colonization and novel anti-infective drugs.

## Materials and methods

### Bacterial strains, mutant library, and culture conditions

The complete list of strains and plasmids used in this study is shown in S9 Table. The highly saturated *Himar 1* Mariner transposon mutant library has been previously described [27]. The *C*. *jejuni* 81–176 strains were grown on brucella broth agar or on blood agar plates (Trypticase soy agar supplemented with 5% sheep blood) at 37°C in an incubator equilibrated to a 10% CO_2_ atmosphere. The *C*. *jejuni* transformants were selected on plates supplemented with 50 μg/ml kanamycin, 7.5 μg/ml chloramphenicol, and 10 μg/ml erythromycin as indicated. For liquid cultures, *C*. *jejuni* strains were grown in brain heart infusion (BHI) medium with no antibiotics added in most cases except for the growth of the mutant library or defined *C*. *jejuni* mutant strains (see below). All *C*. *jejuni* strains were stored at −80°C in BHI broth containing 30% glycerol.

### Screening of the transposon mutant library for their ability to grow in vitro

For growth in rich-medium experiments, approximately 10^8^ CFUs of the *C*. *jejuni* transposon mutant library were plated on blood agar in 15-cm petri dishes, and the bacterial cells were collected after 48 hours. Six replicates of this growth condition were processed as described below. For growth in liquid defined minimal medium, 10^8^ CFUs of the *C*. *jejuni* transposon mutant library were added to 4 ml of DMEM (GIBCO; catalogue number 11965) supplemented with 20 mM Asp, Gln, or Ser. The culture tubes were placed on a rotating wheel in 10% CO_2_ atmosphere for 48 hours, and the bacterial cells were collected by centrifugation. Three replicates for each of these growth conditions were processed as described below. DNA extraction and sequencing were carried out as previously described [27]. The sequencing data were analyzed using the INSeq_pipeline_v2 package [89]. For this analysis, the sequencing data of each sample were normalized to total counts per million (i.e., the number of transposon insertions at each site was multiplied by 10^6^ and then divided by the total number of insertions in the sample) [90,91]. This normalization step enabled comparison of the relative abundance change of each insertion mutant in the mutant library across different samples. Alternatively, the data were normalized using the median values of the number of reads. In this case, the median read number for all transposon insertions was calculated in each sample and normalized to a median read number of 50. To identify significant fitness genes under different growth conditions, a Z-test was applied to all gene mutants whose log-transformed output:input ratios were significantly different from the overall distribution across all biological replicates [89]. Consequently, only genes with a q-value of <0.05 from the Z-test were considered significantly altered from the input. All deep-sequencing data filtering, normalization, mapping, and statistical analysis were conducted in Perl and R.

### Screening of the transposon mutant library for their ability to colonize the mouse intestine

All animal experiments were conducted according to protocols approved by the Yale University Institutional Animal Care and Use Committee. Six- to eight-week-old C57BL/6 mice were treated with antibiotics in drinking water for 4 weeks to eradicate their commensal gut flora and allow robust *C*. *jejuni* colonization as previously described [26]. Antibiotics were added to drinking water at the following concentration: ampicillin, 1 mg/ml; neomycin sulfate, 1 mg/ml; ciprofloxacin 200 μg/ml; metronidazole, 1 mg/ml; and vancomycin, 500 μg/ml. Antibiotics were removed from drinking water 2 days prior to the oral administration through stomach gavage of 100 μl of sodium bicarbonate (to neutralize the stomach pH) followed by the oral administration of 10^9^ CFUs of *C*. *jejuni* in 100 μl of PBS. Mutants were reisolated 4 and 21 days after the mice were inoculated with the transposon insertion pools. Analysis of the mutants recovered 21 days post infection revealed a marked increase in insertions within the pVir and pTet plasmids with a drastic reduction in the representation of the mutant pool. Although the reasons for the “blooming” of these mutants late in infection are unclear, their emergence precluded the use of this time point to analyze *C*. *jejuni* colonization determinants. Consequently, the mutant library screen was performed by killing the mice and homogenizing their ceca with a tissue homogenized 4 days after infection. Bacteria were recovered from the cecum homogenates by plating on 15-cm blood agar petri dish plates (3 plates per cecum sample) containing *Campylobacter* selective supplements (Karmali, Oxoid SR0167). Colonies were scraped off the plates, and the genomic DNA was isolated as previously described [27]. The INseq DNA sample preparation, sequencing, and data analysis were carried out as described above.

### *C*. *jejuni* mutant strain construction

*C*. *jejuni* 81–176 knockout mutant strains were constructed as previously described [41]. Briefly, flanking regions of the selected open reading frames (ORFs) were amplified with specific primers, linked to either side of a kanamycin (*aphA3*), an erythromycin (*erm*), or a chloramphenicol (*cat*) resistance cassette, and cloned into the pBluescript II SK using the Gibson assembly protocol [92]. The resulting plasmids were used to move the mutated alleles into the chromosome of *C*. *jejuni* 81–176 by natural transformation and allelic recombination. Complementation of the mutant strains of *C*. *jejuni* was achieved by introducing a wild-type copy of the gene at the *hsdM* locus as previously described [93]. The plasmids used to generate the mutants are listed in S9 Table.

### In vivo colonization competition assays

Six- to eight-week-old C57BL/6 mice were infected by oral gavage with 10^9^ CFUs of the different *C*. *jejuni* mutants mixed with an equal number of the wild-type parent strain. To enumerate bacterial loads in the cecum, mice were killed 4 days after infection, ceca were homogenized in 3 ml PBS containing 0.05% sodium deoxycholate, and the dilutions were plated on blood agar plates with *Campylobacter* selective supplements with and without the selection antibiotics to determine the CFUs of the wild-type and mutant strains.

### Isotopologue profiling

Cultivation of *C*. *jejuni* for isotopologue analysis was performed as described in S1 Text. Sample preparation for isotopologue analysis by GC/MS was performed as previously described [94]. During sample preparation, the acidic hydrolysis of cellular proteins did not allow the determination of Asn, Cys, Gln, Met, and Trp: Cys and Met are destroyed during the hydrolysis procedure, whereas Asn and Gln are converted to Asp and Glu, respectively. Consequently, the values for Asp and Glu correlate with the averages of Asn/Asp and Gln/Glu [95,96].

### Amino acid uptake by *C*. *jejuni*

*C*. *jejuni* 81–176 was grown in 50 ml Hank’s Balanced Salt Solution supplemented with iron(II)ascorbate (Sigma), MEM Vitamin Solution (Invitrogen), and 1% Casamino acids (Roth) as a carbon source. The liquid cultures were incubated under reduced-oxygen atmosphere using Anaerocult C-Packs (Merck) at 37°C under shaking (150 rpm) conditions. The uptake of amino acids through *C*. *jejuni* 81–176 was investigated by analyzing the changes in the amount of the amino acids in the culture supernatants. Samples of 2 ml supernatant were taken at the indicated time points, the bacterial cells were removed by centrifugation (17,000 g, 5 min, 4°C), and an aliquot of the filtrated supernatant was used for GC/MS analysis. The 100 μl aliquot of the supernatant was diluted in 500 μl water, containing 4 μg of ribitol as an internal standard. The samples were mixed, dried under vacuum at room temperature, and stored at −20°C. Derivatization of the samples was done with 40 μl pyridine, containing methoxyamine hydrochloride (20 mg/ml) and 60 μl N-Methyl-N-trimethylsilyltrifluoro-acetamide (MSTFA). The GC/MS analysis was done on a Thermo GC Ultra coupled to a DSQII mass spectrometer equipped with an AS3000 autosampler (ThermoScientific, Dreieich, Germany) as described before [97], with the following exceptions: helium flow was set to 1.1 ml/min, and the temperature was increased to a final 325°C. The solvent delay time was 5.80 min. Data analysis was performed with Metabolite Detector (version 2.07; [98]), and quantification was done using 1 unique fragment ion for each metabolite. For statistical analysis, data were first normalized by dividing the peak area of every detected compound in each sample by the peak area of the respective internal standard ribitol. Afterwards, the mean and the standard deviation were calculated from 3 biological samples with 3 technical replicates each.

### Defined drop-out amino acid medium (DAAM) for *C*. *jejuni* liquid cultures

To investigate the growth properties of amino-acid auxotrophic *C*. *jejuni* 81–176 mutants, the different strains were cultivated in a defined DAAM based on Hank’s Balanced Salt Solution with each amino acid present in 2 mM concentration supplemented with vitamin mix, 10 μM ferrous ascorbate, and 20 mM lactate as described before [61].

### In vitro growth and motility plate assays

To compare the growth of the *C*. *jejuni ktr* and *kdp* mutants with the wild-type strain in the presence or absence of K^+^, strains were inoculated (starting OD_600_ of 0.02) into 4 ml of liquid defined rich medium (S10 Table) with or without the addition of 0.5 or 5 mM KCl. Cultures were incubated on a rotating wheel under 10% CO_2_ atmosphere, and the OD_600_ were measure at the indicated times after inoculation. All experiments were performed at least 3 times.

For the motility assay, the optical density of the bacterial cultures was adjusted to an OD_600_ of 0.3, and 10 μl was spotted onto soft agar (0.5%, wt/vol). The plates were incubated for 24 hours at 37°C, and the swarming diameters of the different strains were compared to the wild type and the nonmotile *C*. *jejuni* Δ*motA* mutant strain.

## Supporting information

S1 Fig Labeling of polar metabolites in *C*. *jejuni* 81–176 upon cultivation with [3-^13^C_1_]Ser. Shown are the ^13^C-incorporation (^13^C-excess) and the relative isotopologue distributions into polar metabolites isolated from the cytoplasm of *C*. *jejuni* after incubation with [3-^13^C_1_]Ser. Illustrated are the means ± SD of 6 measurements with the colored boxes indicating the relative isotopologue contributions [%] with 1, 2, 3, 4, 5, 6, 7, 8 and 9 ^13^C-atoms corresponding to M+1, M+2, M+3, M+4, M+5, M+6, M+7, M+8 and M+9, respectively (see S11 Table).
(TIF)

S2 Fig Amino acid uptake ability of *C*. *jejuni* 81–176. **(A)** Scheme presenting the predicted amino acid uptake capacity of *C*. *jejuni* 81–176 according to the INSeq analysis. Viable mutants with transposon insertions in the indicated amino acid biosynthesis pathways have been identified in the screen suggesting the import of respective amino acids. No transposon insertions in the glycine biosynthesis genes *glyA* have been identified and the aminotransferase required for the generation of Ala from Pyr has not yet been reported. **(B)** Growth analysis of *C*. *jejuni* 81–176 wild-type strain and amino acid auxotrophic mutants in defined DAAM media in the presence or absence of the indicated amino acids. The maximal OD_600_ within 48 h of incubation are depicted and represent the means ± SD of three independent experiments (see S12 Table).
(TIF)

S3 Fig Amino acid utilization by *C*. *jejuni* 81–176 determined by exometabolome analysis. The uptake of amino acids by *C*. *jejuni* 81–176 was examined by measuring their concentrations in the culture supernatants within 24 h of cultivation relative to their original concentration prior to bacterial inoculation, which was considered to be 100%. Shown are the mean values ± SD of three independent experiments measured in triplicates (see S13 Table). Significant amino acid uptake were detectable for Asp, Glu, Pro, Ser, Cys, Met and Thr with * *P* < 0.05, ** *P* < 0.01 and *** *P* < 0.001 calculated by Student’s unpaired *t-test*.
(TIF)

S4 Fig Colony forming units of commensal bacteria in mice feces before and after antibiotic treatment. Shown is the intestinal bacterial load after antibiotic treatment of animals immediately prior to infection with *C*. *jejuni*. Collected feces samples from each mouse were dissolved and diluted serially with PBS buffer, then plated on blood agar in duplicates. One half of plates were incubated in 10% CO_2_ incubator as “aerobic condition”, the other half of plates were placed in anaerobic jar as “anaerobic condition”. CFU determination was carried out after 48 h incubation (see S14 Table).
(TIF)

S5 Fig *In vivo* fitness of *C*. *jejuni* insertion mutants determined by INSeq analysis. Relative abundance of *C*. *jejuni* insertion mutants in the inoculum and the mouse cecum samples after infection for 4 **(A)**, 7 **(B)** and 21 **(C)** days. In **(A)** and **(B)**, each point represents the average abundance of read numbers of a single gene obtained from 13 or 5 mice, respectively, and are normalized to per million reads. In **(C)**, each point represents the abundance of read numbers of a single gene from a single mouse infection, which is normalized to per million reads. (**D**) Summary of number of insertion mutants recovered from mouse ceca at the indicated days after infection.
(TIF)

S6 Fig Log2 (output/input) ratio distribution of all insertions during mouse colonization. Histogram depicting the number of genes (y axis) that exhibited the indicated log2 [fold change (output/input)] change (x axis) in the numbers of transposon insertions recovered from infected mice relative to the number of transposon insertions in the original inoculum. Areas colored with red represent genes whose number of transposon insertions showed a significant decrease after mouse infection. For this analysis the data were normalized using the median values of the number of reads (see Materials and Methods; S4 Table).
(TIF)

S7 Fig Contribution of a *C*. *jejuni* motility-associated gene cluster to mouse colonization. (**A**)Blue and red bars indicate the normalized read number of each insertion site within the different ORFs in the input and output pool, respectively. Motility assays (**B**) and growth curves (see S12 Table) (**C**) for wild-type *C*. *jejuni* 81–176 and the ΔCJJ81176_0479, ΔCJJ81176_0480, or ΔCJJ81176_0481 mutants.
(TIF)

S8 Fig Role of amino acid biosynthesis of *C*. *jejuni* 81–176 in mouse colonization. Illustrated is the impact of amino acid biosynthesis pathways in *C*. *jejuni* mouse intestinal colonization as determined by INSeq analysis. Numbers indicate the log2 value of fold change (intestine/inoculum) in the number of insertions in the indicated genes and are derived from the raw data in S3 Table. Values below -6.2 indicate mutations led to a statistically significant colonization defect. *: denotes genes showing a limited number of insertions within the library and no insertions within the pooled of mutants recovered from the intestine. §: input pool of INSeq analyses lack mutants with transposon insertions within this gene.
(TIF)

S9 Fig Amino acid composition of all predicted proteins encoded by *C*. *jejuni* 81–176. The frequency of all amino acids in all predicted proteins are shown and branched-chain amino acids are highlighted in orange. Calculations were carried out using BacMap (http://wishart.biology.ualberta.ca/BacMap/cgi/getGraphs.cgi?accession=NC_008787&ref=index_2.html).
(TIF)

S10 Fig Growth properties of serine-biosynthesis defective *C*. *jejuni* mutants. Wild type *C*. *jejuni* 81–176 (WT) and the indicated isogenic mutant strains (*serA*: D-3-phosphoglycerate dehydrogenase; *sdaA*: L-serine ammonia-lyase/dehydratase) were grown in defined medium (DAAM) in the presence or absence of serine as indicated. Lactate or glutamate are provided as general energy/carbon source. Values represent the mean ± SD of the maximal optical density (OD_600_) reached after 48 h of growth in 3 independent experiments (see S12 Table).
(TIF)

S11 Fig Role of methionine and S-adenosyl methionine (SAM) biosynthesis in *C*. *jejuni* 81–176 *in vivo* growth. Illustrated is the impact of methionine and SAM biosynthesis in *C*. *jejuni* mouse intestinal colonization as determined by INSeq analysis. Numbers indicate the log2 value of fold change (intestine/inoculum) in the number of insertions in the indicated genes and are derived from the raw data in S3 Table. Values below -6.2 indicate mutations led to a statistically significant colonization defect. *: denotes genes showing a limited number of insertions within the library and no insertions within the pooled of mutants recovered from the intestine.
(TIF)

S12 Fig ^13^C-incorporation and isotopologue profiles of protein-derived amino acids in *C*. *jejuni* 81–176 after incubation with [5-^13^C_1_]-Glu in DMEM medium. Black columns on the left y axis represent percentage of ^13^C-excess (mol %) into the respective protein-derived amino acids. The colored columns on the right y axis depict the percentages of labeled isotopologues comprising up to six labeled ^13^C atoms (M+1 to M+6). Values are the means ± SD of 6 measurements (see S11 Table).
(TIF)

S13 Fig Schematic overview of the ^13^C-flux into protein-derived amino acids after catabolism of [5-^13^C_1_]-Glu in *C*. *jejuni* 81–176. The positions of the ^13^C-label of the amino acid isotopologues are indicated by the colored dots. Due to the stereoisomerism of the TCA-cycle intermediates succinate and fumarate, it is not possible to distinguish between the C_1_- or the C_4_-carbon atom ^13^C-label. Consequently, the possibilities of the ^13^C in OAA and its derived amino acids are indicated in red and orange, both colors displaying a 50% possibility that the carbon atom acquired the label. Green arrows depict the synthesis of the amino acids from their respective precursor molecules. The individual reactions of the multi-step amino acid biosynthesis pathways are not displayed.
(TIF)

S14 Fig Role of the “acetate switch” in mouse colonization. Six mice were inoculated with an equal number of wild-type *C*. *jejuni* 81–176 and the Δ*ackA* isogenic mutant strain via oral gavage. Competitive indices (CI) were calculated as the ratio of the CFU of the Δ*ackA* mutant over wild type recovered from the ceca of infected mice (see S14 Table).
(TIF)

S15 Fig Impact of the non-oxidative pentose phosphate pathway (PPP) on the growth of *C*. *jejuni* 81–176. Shown is the impact of the inactivation of genes encoding components of the PPP pathway in *C*. *jejuni* mouse intestinal colonization as determined by INSeq analyses. Numbers indicate the log2 value of fold change (intestine/inoculum) in the number of insertions in the indicated genes and are derived from the raw data in S3 Table. Values below -6.2 indicate mutations led to a statistically significant colonization defect. Genes with no insertions in the library are denoted in greeen. *: denotes genes showing a limited number of insertions within the library and no insertions within the pooled of mutants recovered from the intestine.
(TIF)

S16 Fig Impact of the purine and pyrimidine biosynthesis pathways on the *in vivo* growth of *C*. *jejuni* 81–176. Shown is the impact of the inactivation of genes encoding components of the purine and pyrimidine biosynthesis pathways in *C*. *jejuni* mouse intestinal colonization as determined by INSeq analyses. Numbers indicate the log2 value of fold change (intestine/inoculum) in the number of insertions in the indicated genes and are derived from the raw data in S3 Table. Values below -6.2 indicate mutations led to a statistically significant colonization defect. Red arrows denote that the number of insertions within the gene that catalyze the indicated reaction was significantly reduced within the pooled of mutants recovered from the mouse intestine relative to the inoculum. Green arrows indicate that the genes encoding the enzymes that catalyze the corresponding reactions do not have insertional mutants in the library. *: denotes genes showing a limited number of insertions within the library and no insertions within the pooled of mutants recovered from the intestine.
(TIF)

S17 Fig Core metabolic reactions of *C*. *jejuni* 81–176 that generate carbon dioxide. Selected metabolic reactions in *C*. *jejuni* that release carbon dioxide (CO_2_). Numbers indicate the log2 value of fold change (intestine/inoculum) in the number of insertions in the indicated genes and are derived from the raw data in S3 Table. Values below -6.2 indicate mutations led to a statistically significant colonization defect. Red arrows denote that the number of insertions within the gene that catalyze the indicated reaction was significantly reduced within the pooled of mutants recovered from the mouse intestine relative to the inoculum. In green arrows indicate that the genes encoding the enzyme that catalyzes the corresponding reaction are noted genes with no insertions in our mutant library. *: denotes genes showing a limited number of insertions within the library and no insertions within the pooled of mutants recovered from the intestine.
(TIF)

S1 Table INSeq raw data for each C. jejuni gene after growth under different in-vitro growth condition. (XLSX)

S2 Table List of genes that show similar mutant phenotype according to the Venn diagram depicted in Fig 1B and 1C. (XLSX)

S3 Table INSeq raw data for each gene in mouse colonization experiments. (XLSX)

S4 Table INSeq raw data for each gene in mouse colonization experiments, which were normalized by median nomarlization method. (XLSX)

S5 Table Analysis of potential polar effects of transposon insertions. (XLSX)

S6 Table Growth-promoting substrates utilized by *Campylobacter jejuni* during colonization of gastrointestinal tract. (DOCX)

S7 Table Summary of INSeq data for Tlp and chemotaxis proteins in *C*. *jejuni* 81–176. (XLSX)

S8 Table Summary of potassium channel and transporters in Epsilon-proteobacteria. (XLSX)

S9 Table Strains and plasmids used in this study. (XLSX)

S10 Table Composition of a modified defined DMEM medium for *Campylobacter jejuni* 81–176 used in the indicated studies. (XLSX)

S11 Table Raw data for Figs 2, 5inset, 6C, 6D, S1, S12 and S13. (XLSX)

S12 Table Raw data for Figs 6E, 7E, S2B, S7C and S10. (XLSX)

S13 Table Raw data for S3 Fig. (XLSX)

S14 Table Raw data for Figs 7D, S4 and S14. (XLSX)

S1 Text Supplementary methods. (DOCX)

## Abbreviations

ABC: ATP-binding cassette
acetyl-CoA: acetyl coenzyme A
Ala: alanine
Arg: arginine
Asn: asparagine
Asp: aspartic acid
Cdt: cytolethal distending toxin
CFU: colony-forming unit
CI: competitive index
CMP: cytidine monophosphate
CPS: capsule
Cys: cysteine
DMEM: Dulbecco’s Modified Eagle Medium
EMP: Emden-Meyerhof-Parnas
GA3P: glyceraldehyde-3-phosphate
GC/MS: gas chromatography/mass spectrometry
GDP: guanosine diphosphate
Gln: glutamine
Glu: glutamic acid
Gly: glycine
GTP: guanosine-5'-triphosphate
His: histidine
Ile: isoleucine
INSeq: insertion sequencing
Lac: lactate
Leu: leucine
LIV: leucine-isoleucine-valine
LOS: lipooligosaccharide
Lys: lysine
Met: methionine
NAD: nicotinamide adenine dinucleotide
OAA: oxaloacetate
ORF: open reading frame
PEP: phosphoenolpyruvic acid
Pfk: phosphofructokinase
PFOR: pyruvate ferredoxin/flavodoxin oxidoreductase
Phe: phenylalanine
PLP: pyridoxine-5′-phosphate
PPP: pentose phosphate pathway
Pro: proline
PRPP: phosphoribosyl pyrophosphate
Pyr: pyruvate
Rpe: ribulose-phosphate 3-epimerase
RpiB: ribose 5-phosphate isomerase B
SAM: S-adenosyl methionine
SD: standard deviation
Ser: serine
TCA: tricarboxylic acid
Thr: threonine
TPR: tetratricopeptide repeat
Trp: tryptophan
Tyr: tyrosine
Val: valine
