# Supplementary figures and images for: Metabolic and fitness determinants for in vitro growth and intestinal colonization of the bacterial pathogen Campylobacter jejuni
Source: PLoS Biol. 2017 May 19;15(5):e2001390. doi: 10.1371/journal.pbio.2001390 (PMC5438104; doi:10.1371/journal.pbio.2001390)

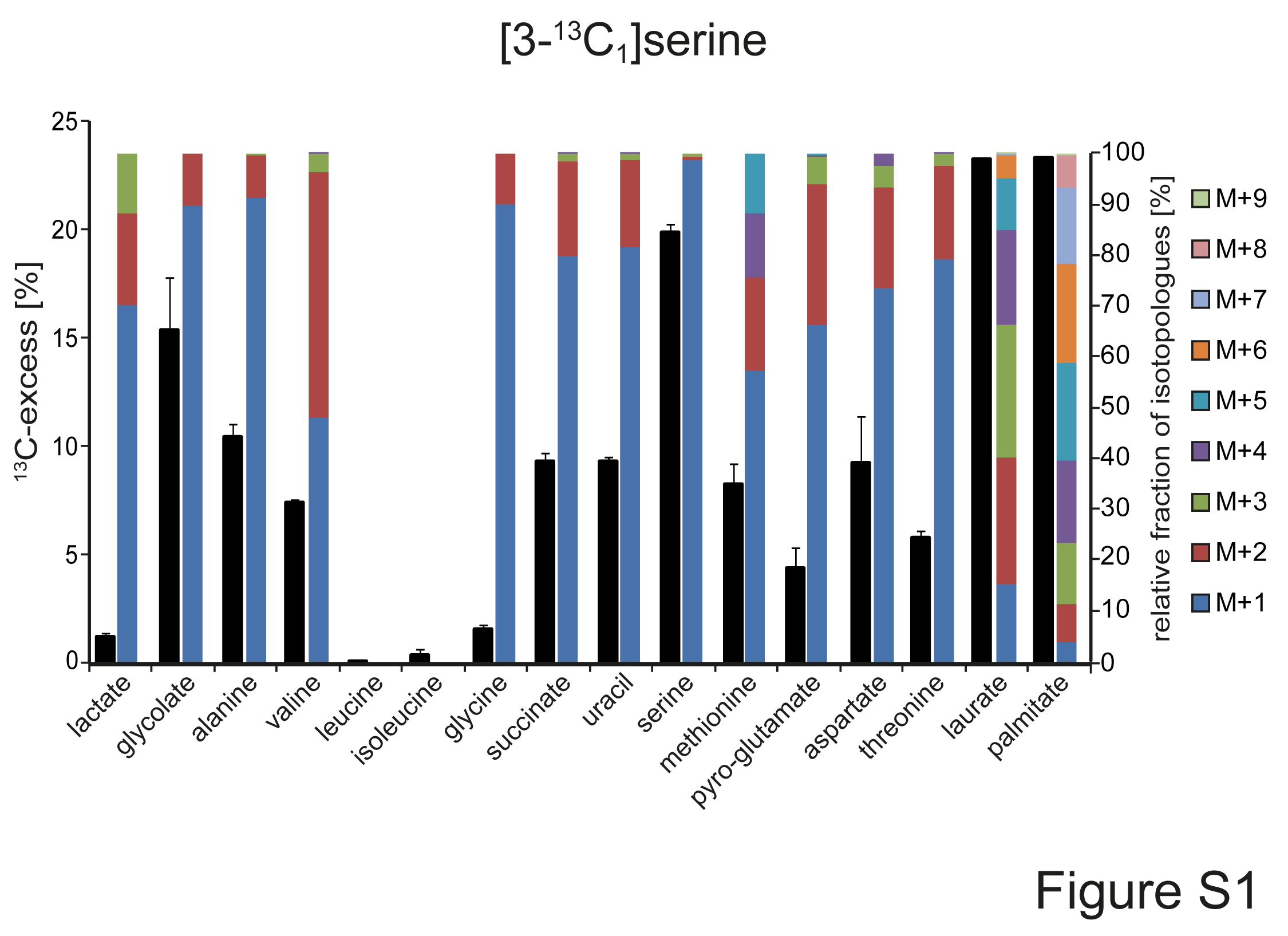

Supplement: S1 Fig — Shown are the 13C-incorporation (13C-excess) and the relative isotopologue distributions into polar metabolites isolated from the cytoplasm of C. jejuni after incubation with [3-13C1]Ser. Illustrated are the means ± SD of 6 measurements with the colored boxes indicating the relative isotopologue contributions [%] with 1, 2, 3, 4, 5, 6, 7, 8 and 9 13C-atoms corresponding to M+1, M+2, M+3, M+4, M+5, M+6, M+7, M+8 and M+9, respectively (see S11 Table). (TIF) [file pbio.2001390.s001.tif]

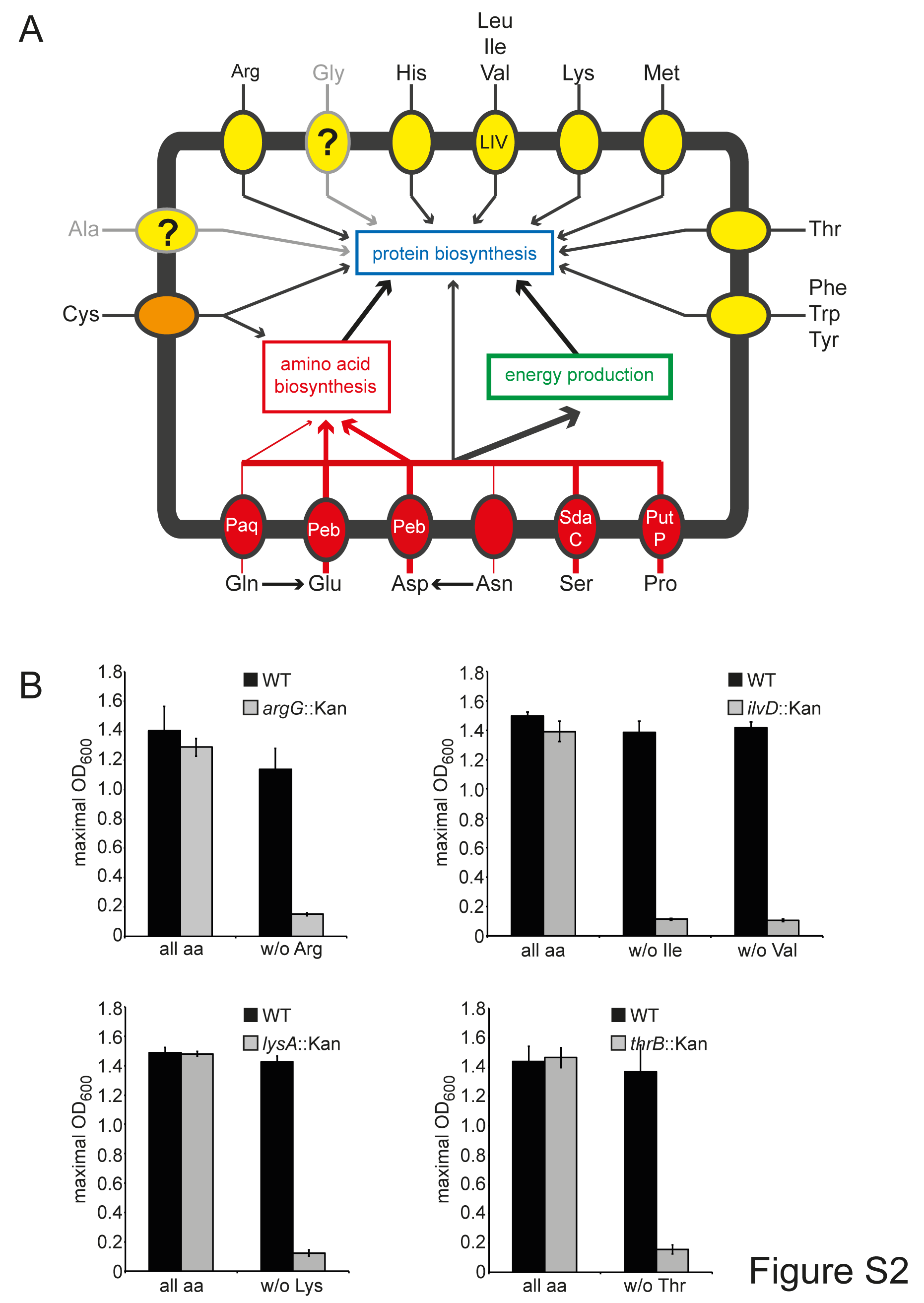

Supplement: S2 Fig — (A) Scheme presenting the predicted amino acid uptake capacity of C. jejuni 81–176 according to the INSeq analysis. Viable mutants with transposon insertions in the indicated amino acid biosynthesis pathways have been identified in the screen suggesting the import of respective amino acids. No transposon insertions in the glycine biosynthesis genes glyA have been identified and the aminotransferase required for the generation of Ala from Pyr has not yet been reported. (B) Growth analysis of C. jejuni 81–176 wild-type strain and amino acid auxotrophic mutants in defined DAAM media in the presence or absence of the indicated amino acids. The maximal OD600 within 48 h of incubation are depicted and represent the means ± SD of three independent experiments (see S12 Table). (TIF) [file pbio.2001390.s002.tif]

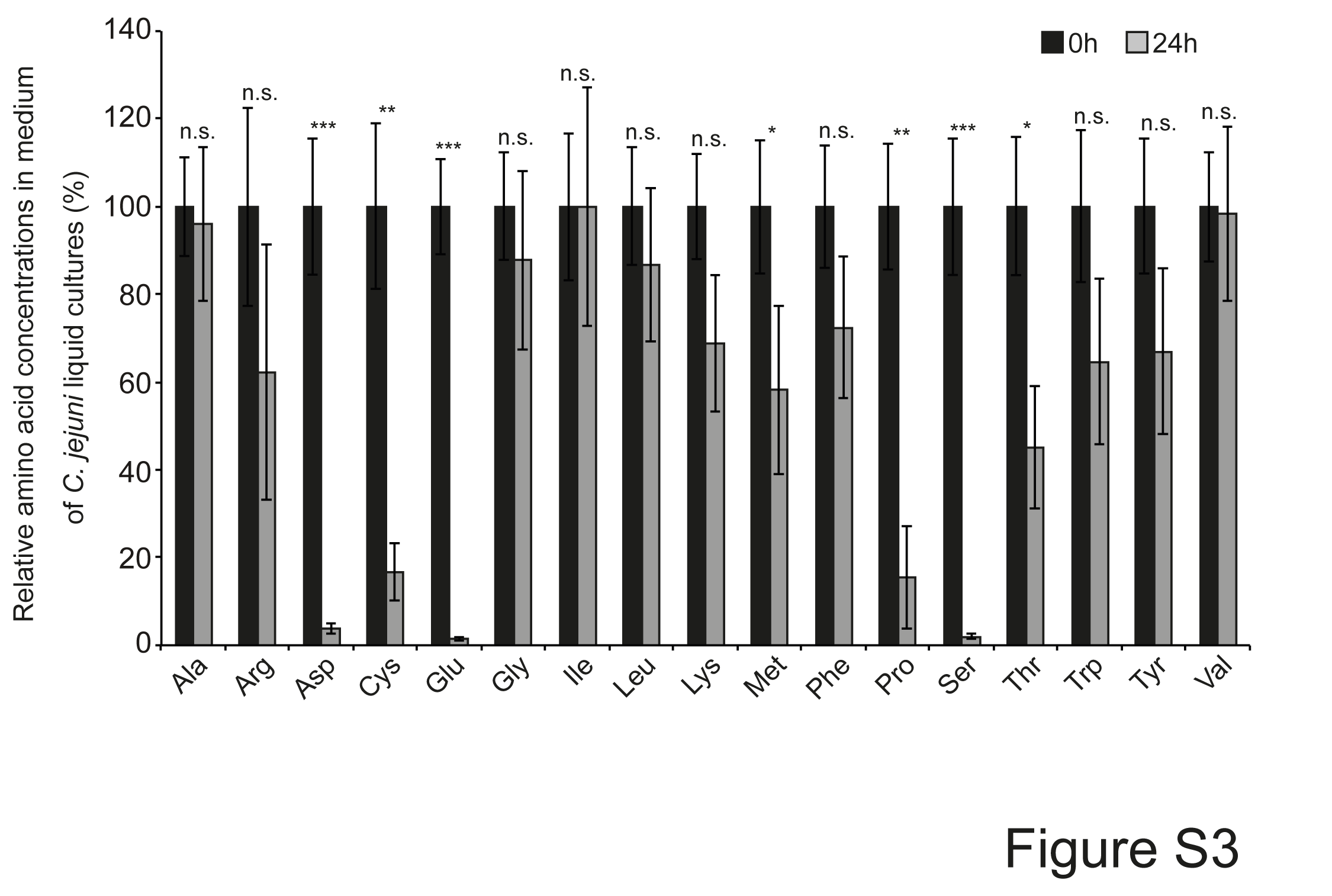

Supplement: S3 Fig — The uptake of amino acids by C. jejuni 81–176 was examined by measuring their concentrations in the culture supernatants within 24 h of cultivation relative to their original concentration prior to bacterial inoculation, which was considered to be 100%. Shown are the mean values ± SD of three independent experiments measured in triplicates (see S13 Table). Significant amino acid uptake were detectable for Asp, Glu, Pro, Ser, Cys, Met and Thr with * P < 0.05, ** P < 0.01 and *** P < 0.001 calculated by Student’s unpaired t-test. (TIF) [file pbio.2001390.s003.tif]

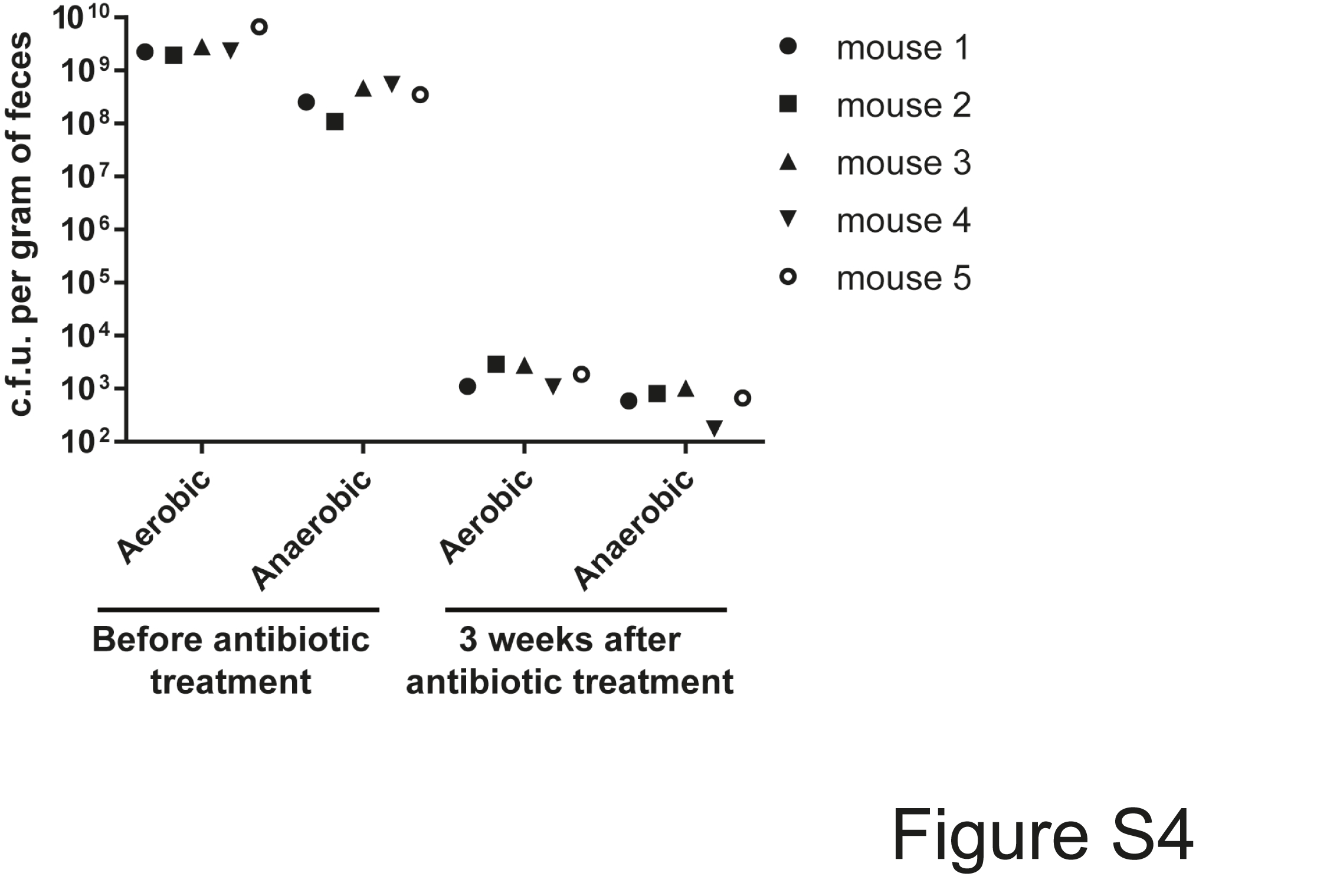

Supplement: S4 Fig — Shown is the intestinal bacterial load after antibiotic treatment of animals immediately prior to infection with C. jejuni. Collected feces samples from each mouse were dissolved and diluted serially with PBS buffer, then plated on blood agar in duplicates. One half of plates were incubated in 10% CO2 incubator as “aerobic condition”, the other half of plates were placed in anaerobic jar as “anaerobic condition”. CFU determination was carried out after 48 h incubation (see S14 Table). (TIF) [file pbio.2001390.s004.tif]

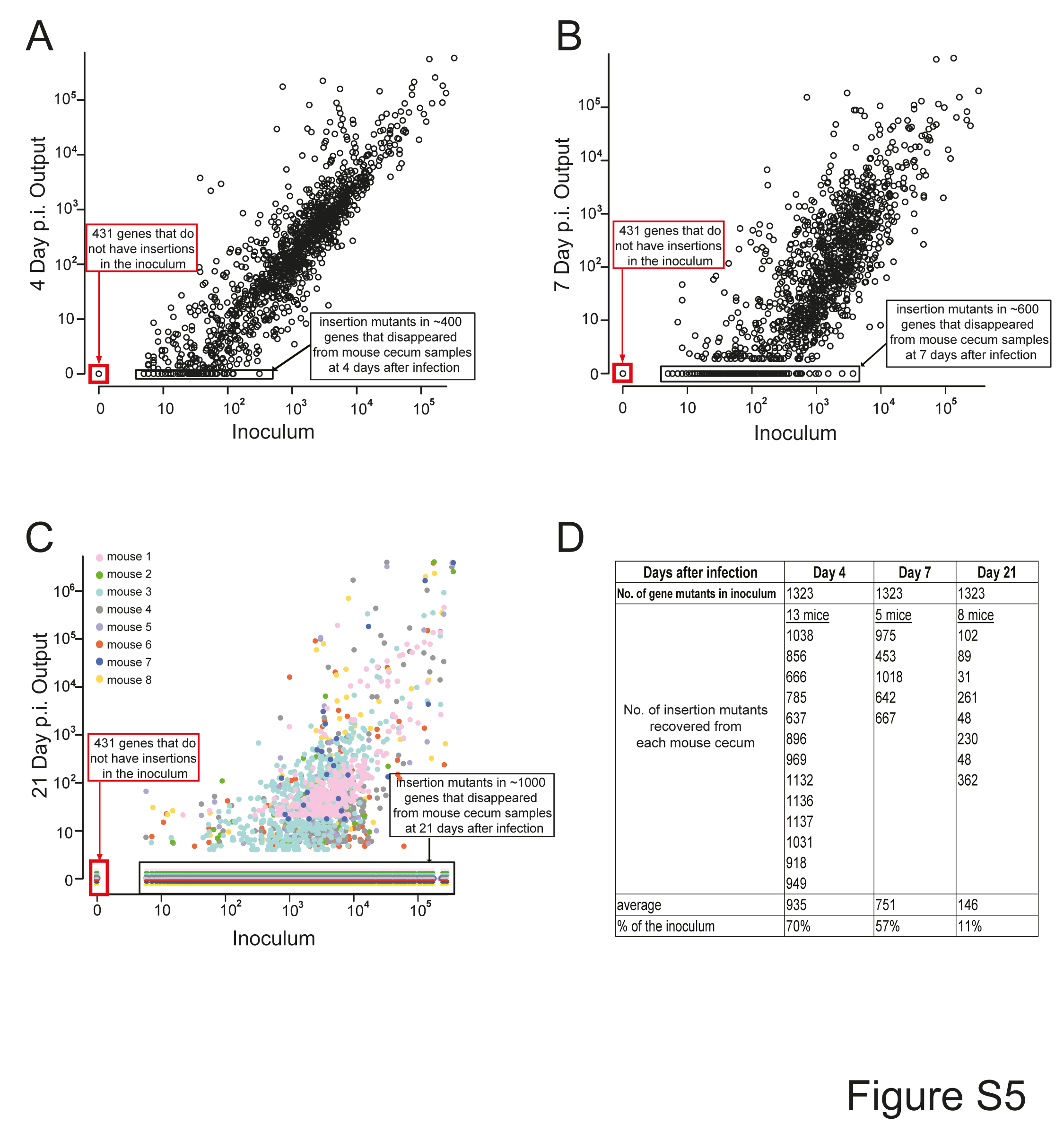

Supplement: S5 Fig — Relative abundance of C. jejuni insertion mutants in the inoculum and the mouse cecum samples after infection for 4 (A), 7 (B) and 21 (C) days. In (A) and (B), each point represents the average abundance of read numbers of a single gene obtained from 13 or 5 mice, respectively, and are normalized to per million reads. In (C), each point represents the abundance of read numbers of a single gene from a single mouse infection, which is normalized to per million reads. (D) Summary of number of insertion mutants recovered from mouse ceca at the indicated days after infection. (TIF) [file pbio.2001390.s005.tif]

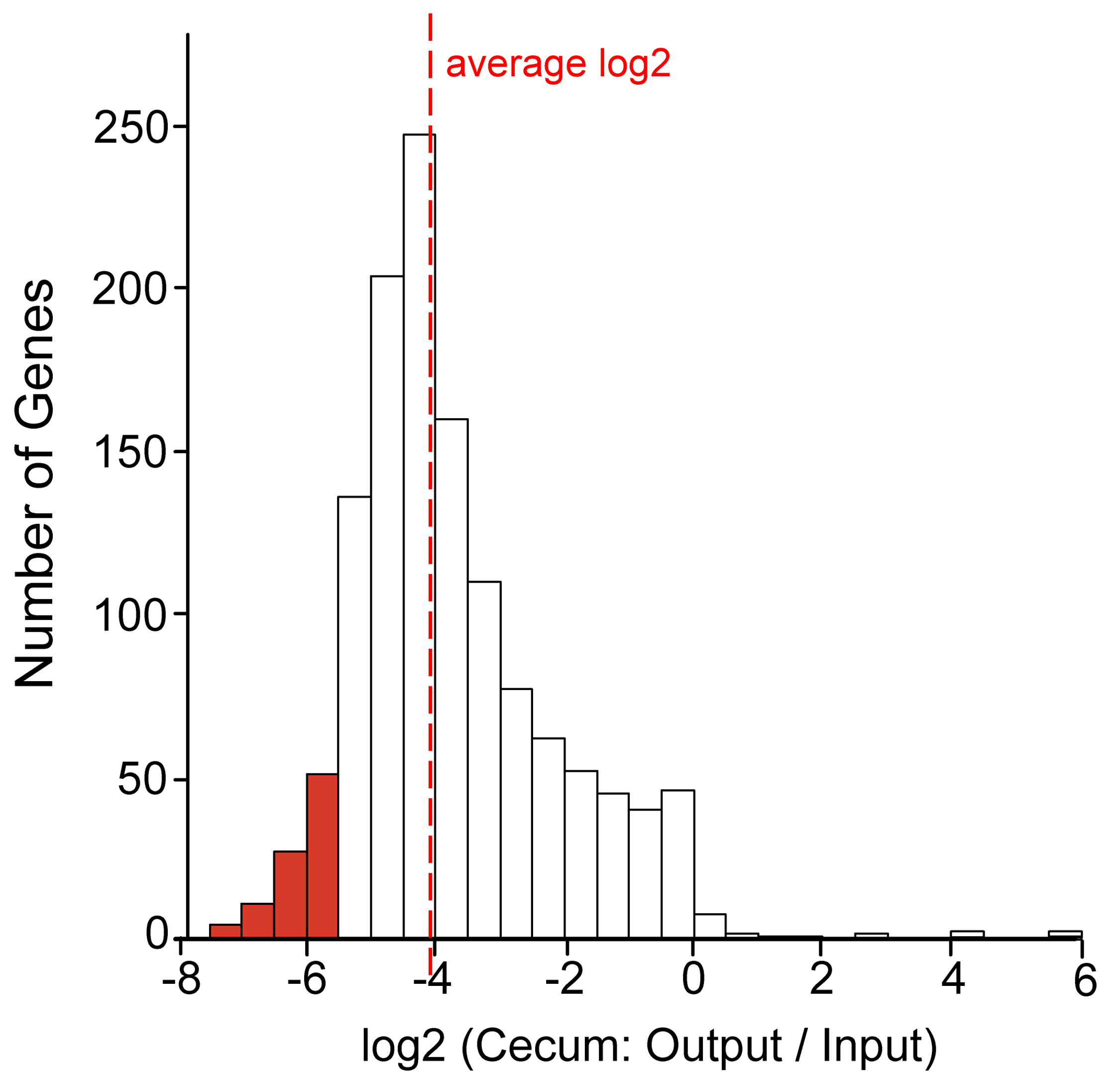

Supplement: S6 Fig — Histogram depicting the number of genes (y axis) that exhibited the indicated log2 [fold change (output/input)] change (x axis) in the numbers of transposon insertions recovered from infected mice relative to the number of transposon insertions in the original inoculum. Areas colored with red represent genes whose number of transposon insertions showed a significant decrease after mouse infection. For this analysis the data were normalized using the median values of the number of reads (see Materials and Methods; S4 Table). (TIF) [file pbio.2001390.s006.tif]

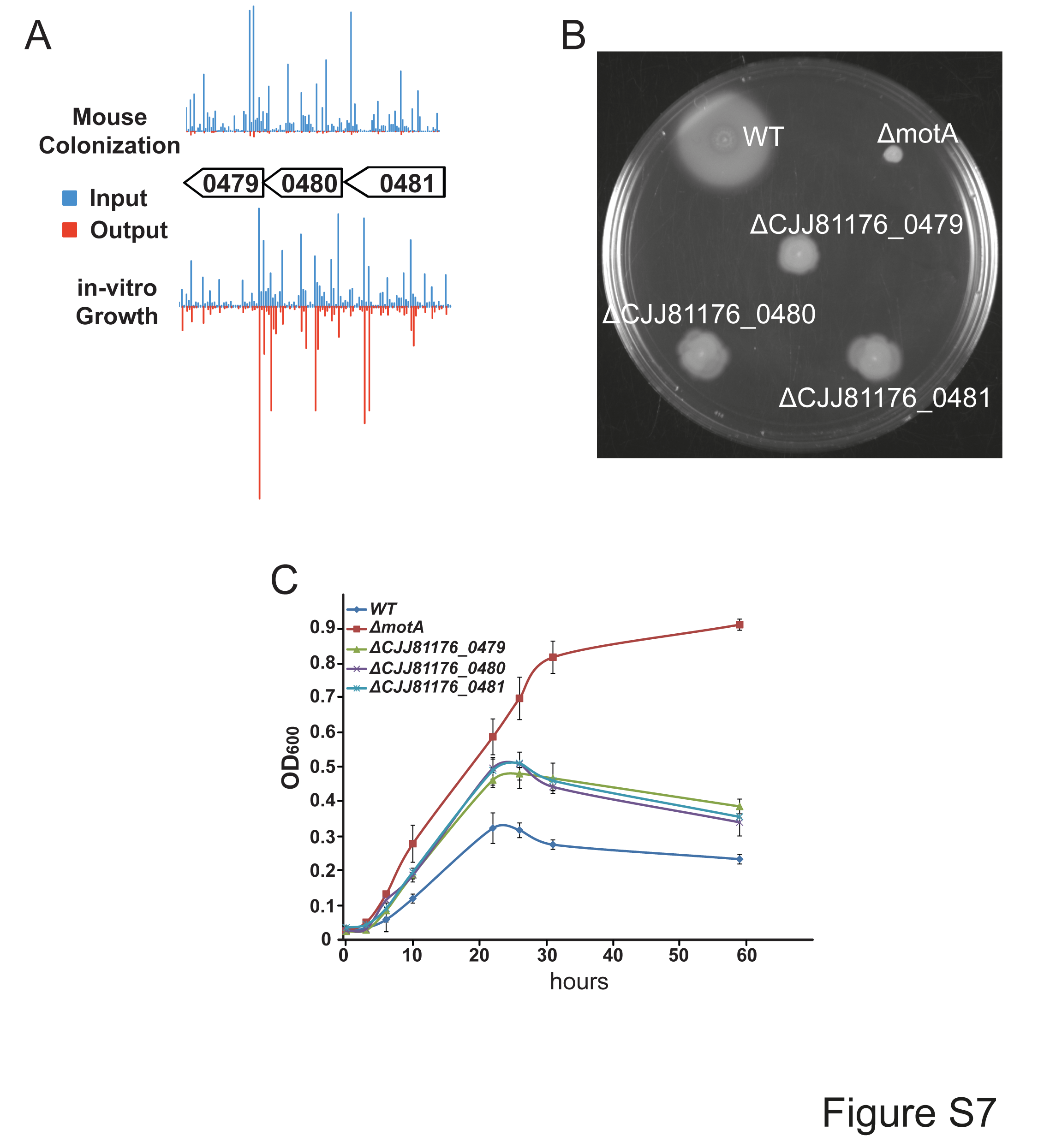

Supplement: S7 Fig — (A)Blue and red bars indicate the normalized read number of each insertion site within the different ORFs in the input and output pool, respectively. Motility assays (B) and growth curves (see S12 Table) (C) for wild-type C. jejuni 81–176 and the ΔCJJ81176_0479, ΔCJJ81176_0480, or ΔCJJ81176_0481 mutants. (TIF) [file pbio.2001390.s007.tif]

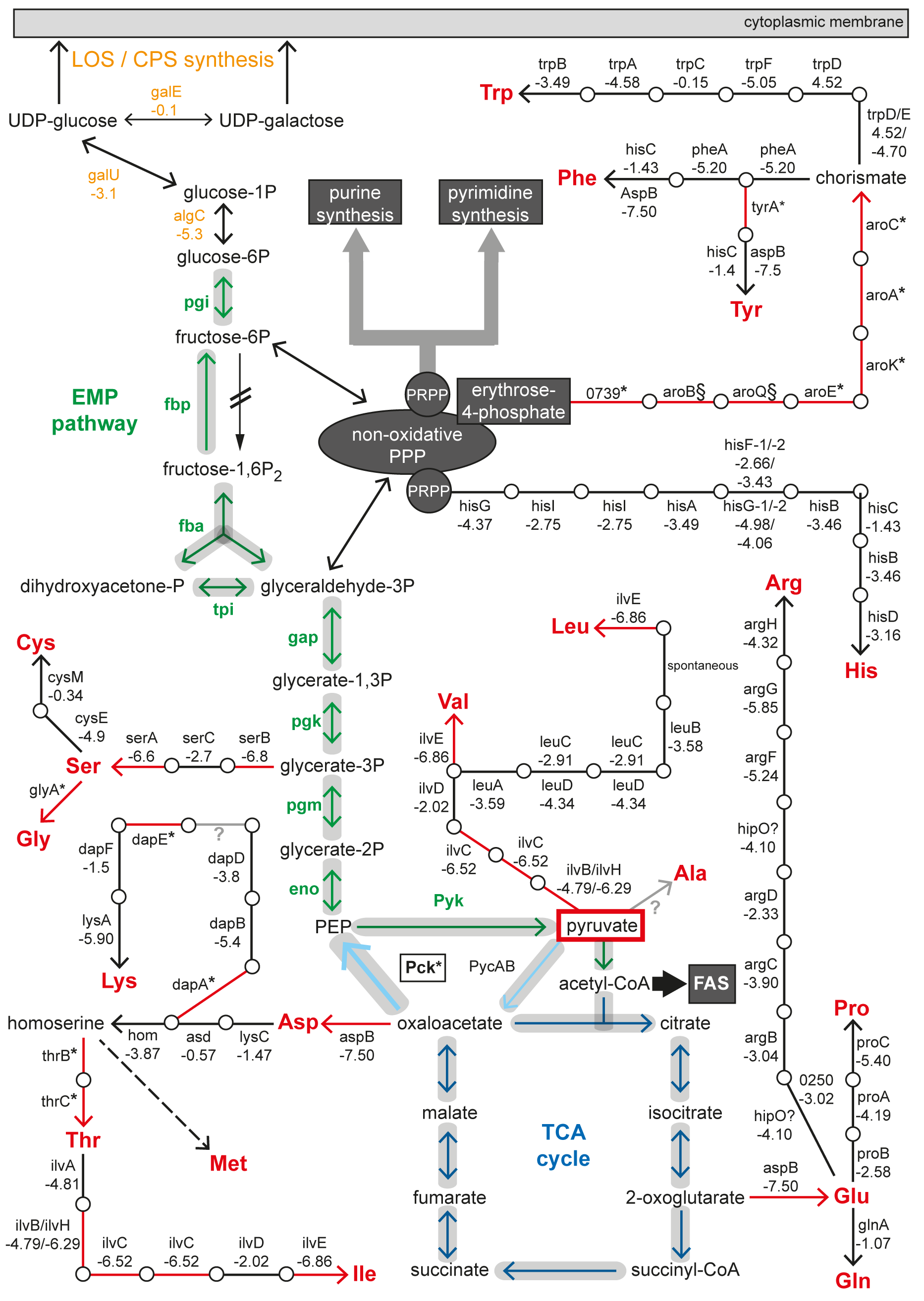

Supplement: S8 Fig — Illustrated is the impact of amino acid biosynthesis pathways in C. jejuni mouse intestinal colonization as determined by INSeq analysis. Numbers indicate the log2 value of fold change (intestine/inoculum) in the number of insertions in the indicated genes and are derived from the raw data in S3 Table. Values below -6.2 indicate mutations led to a statistically significant colonization defect. *: denotes genes showing a limited number of insertions within the library and no insertions within the pooled of mutants recovered from the intestine. §: input pool of INSeq analyses lack mutants with transposon insertions within this gene. (TIF) [file pbio.2001390.s008.tif]

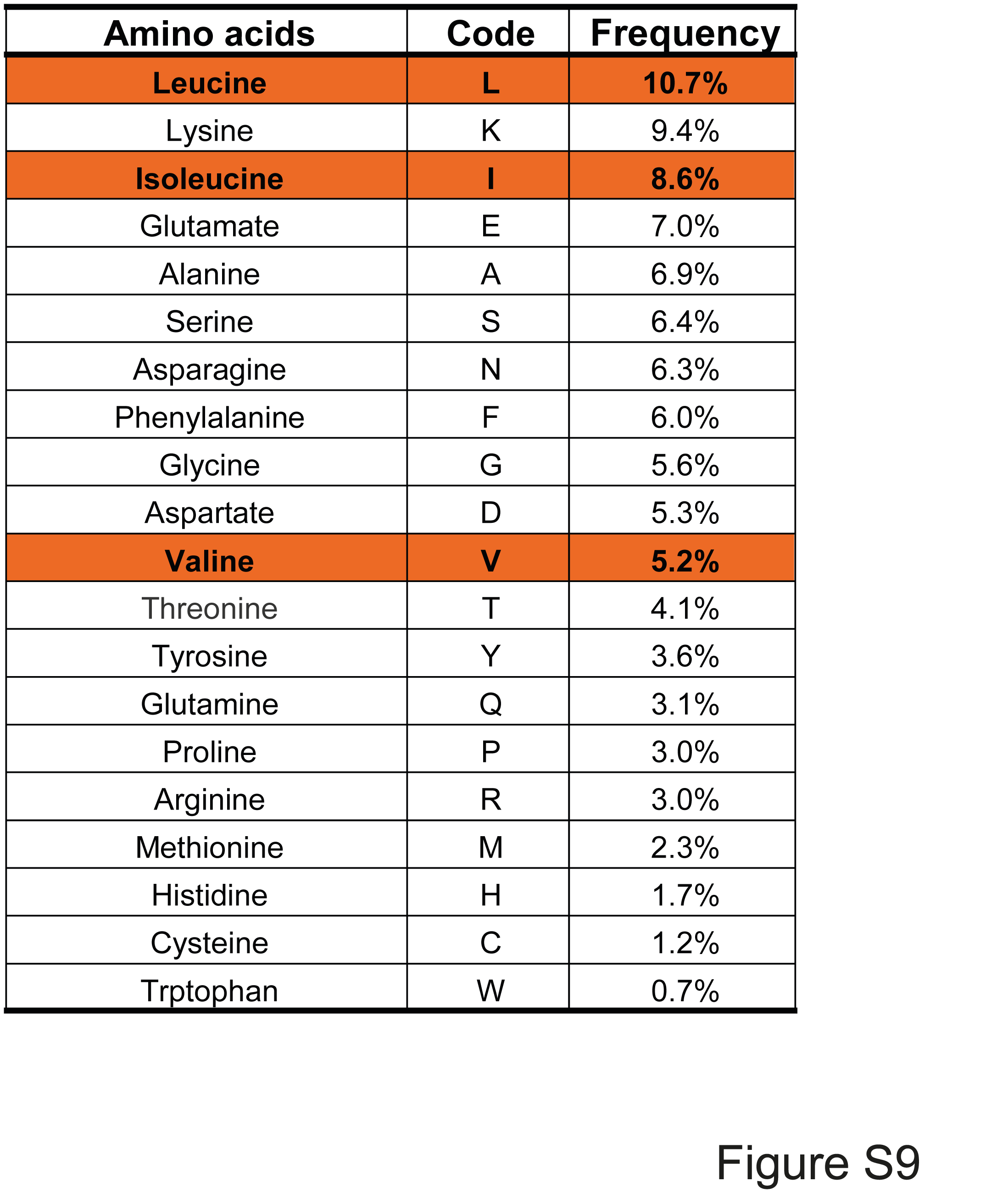

Supplement: S9 Fig — The frequency of all amino acids in all predicted proteins are shown and branched-chain amino acids are highlighted in orange. Calculations were carried out using BacMap (http://wishart.biology.ualberta.ca/BacMap/cgi/getGraphs.cgi?accession=NC_008787&ref=index_2.html). (TIF) [file pbio.2001390.s009.tif]

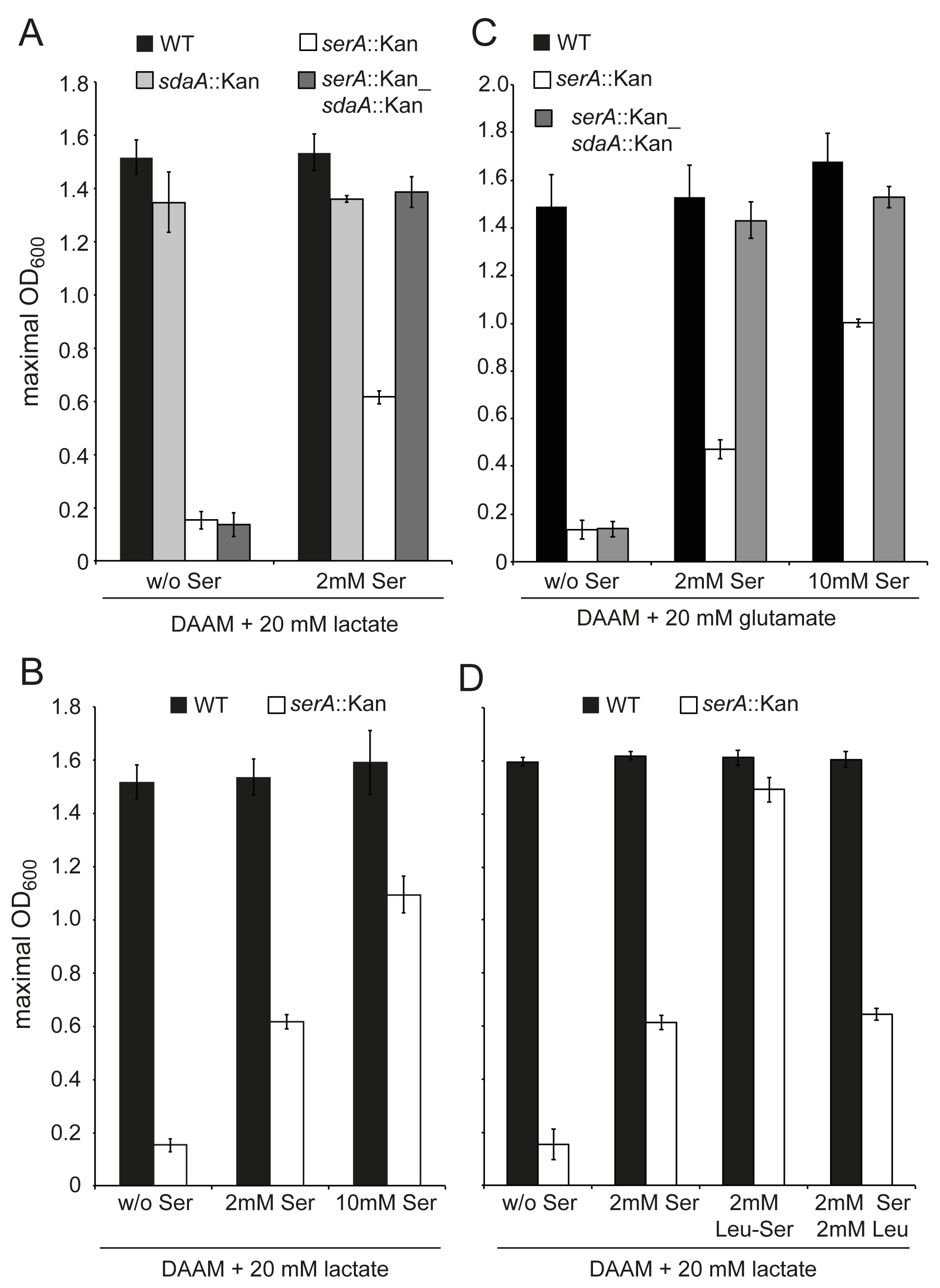

Supplement: S10 Fig — Wild type C. jejuni 81–176 (WT) and the indicated isogenic mutant strains (serA: D-3-phosphoglycerate dehydrogenase; sdaA: L-serine ammonia-lyase/dehydratase) were grown in defined medium (DAAM) in the presence or absence of serine as indicated. Lactate or glutamate are provided as general energy/carbon source. Values represent the mean ± SD of the maximal optical density (OD600) reached after 48 h of growth in 3 independent experiments (see S12 Table). (TIF) [file pbio.2001390.s010.tif]

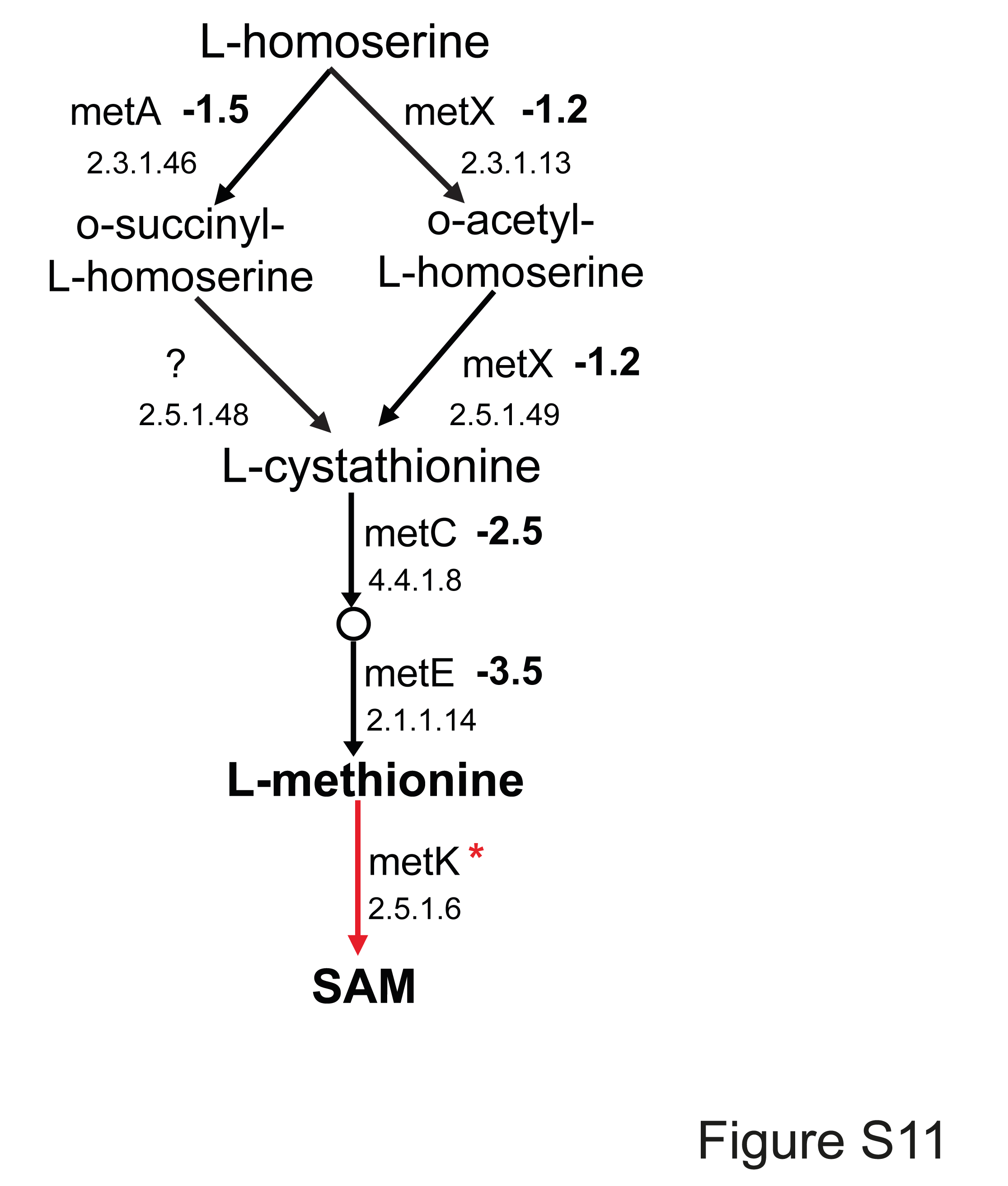

Supplement: S11 Fig — Illustrated is the impact of methionine and SAM biosynthesis in C. jejuni mouse intestinal colonization as determined by INSeq analysis. Numbers indicate the log2 value of fold change (intestine/inoculum) in the number of insertions in the indicated genes and are derived from the raw data in S3 Table. Values below -6.2 indicate mutations led to a statistically significant colonization defect. *: denotes genes showing a limited number of insertions within the library and no insertions within the pooled of mutants recovered from the intestine. (TIF) [file pbio.2001390.s011.tif]

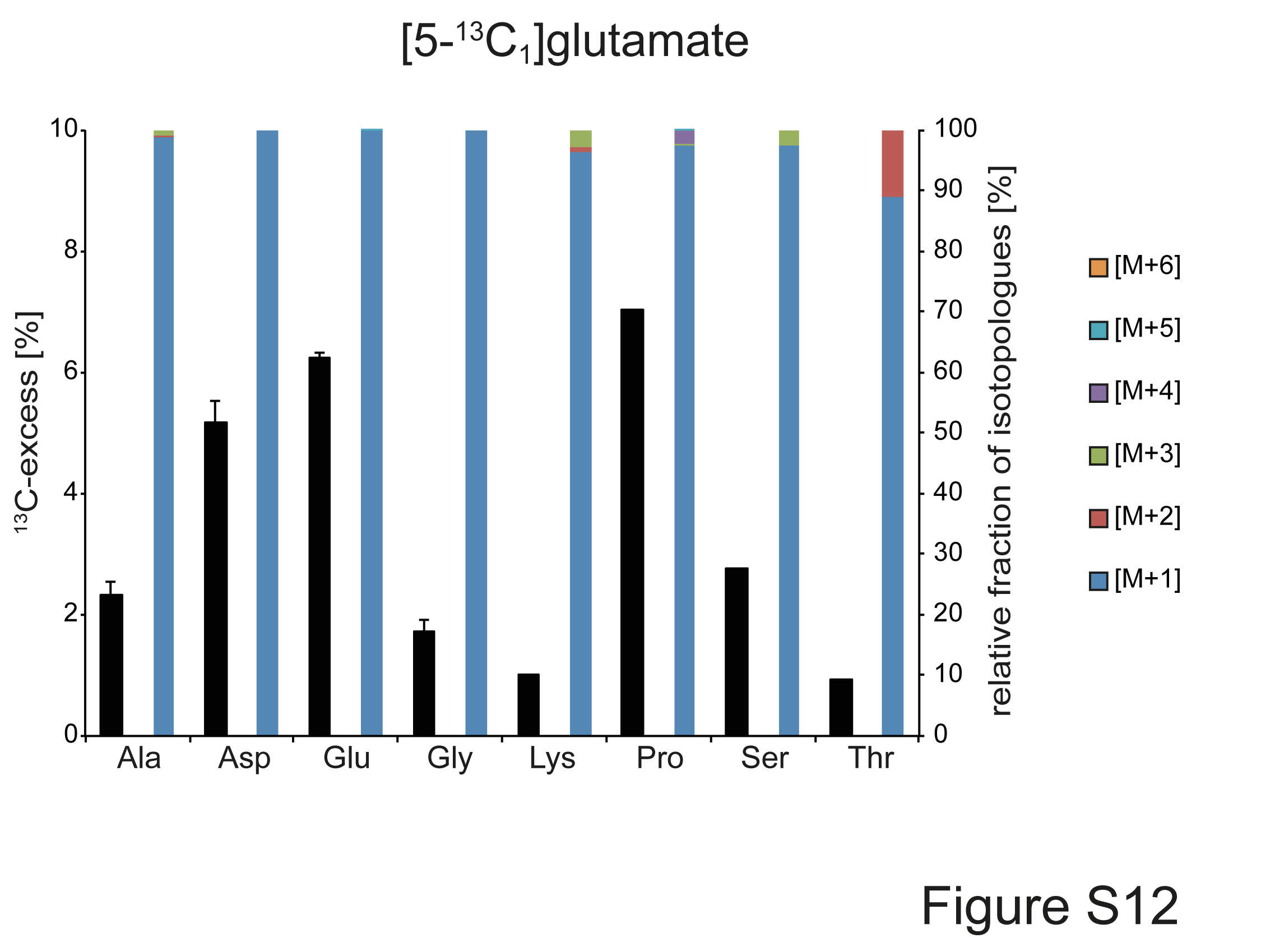

Supplement: S12 Fig — Black columns on the left y axis represent percentage of 13C-excess (mol %) into the respective protein-derived amino acids. The colored columns on the right y axis depict the percentages of labeled isotopologues comprising up to six labeled 13C atoms (M+1 to M+6). Values are the means ± SD of 6 measurements (see S11 Table). (TIF) [file pbio.2001390.s012.tif]

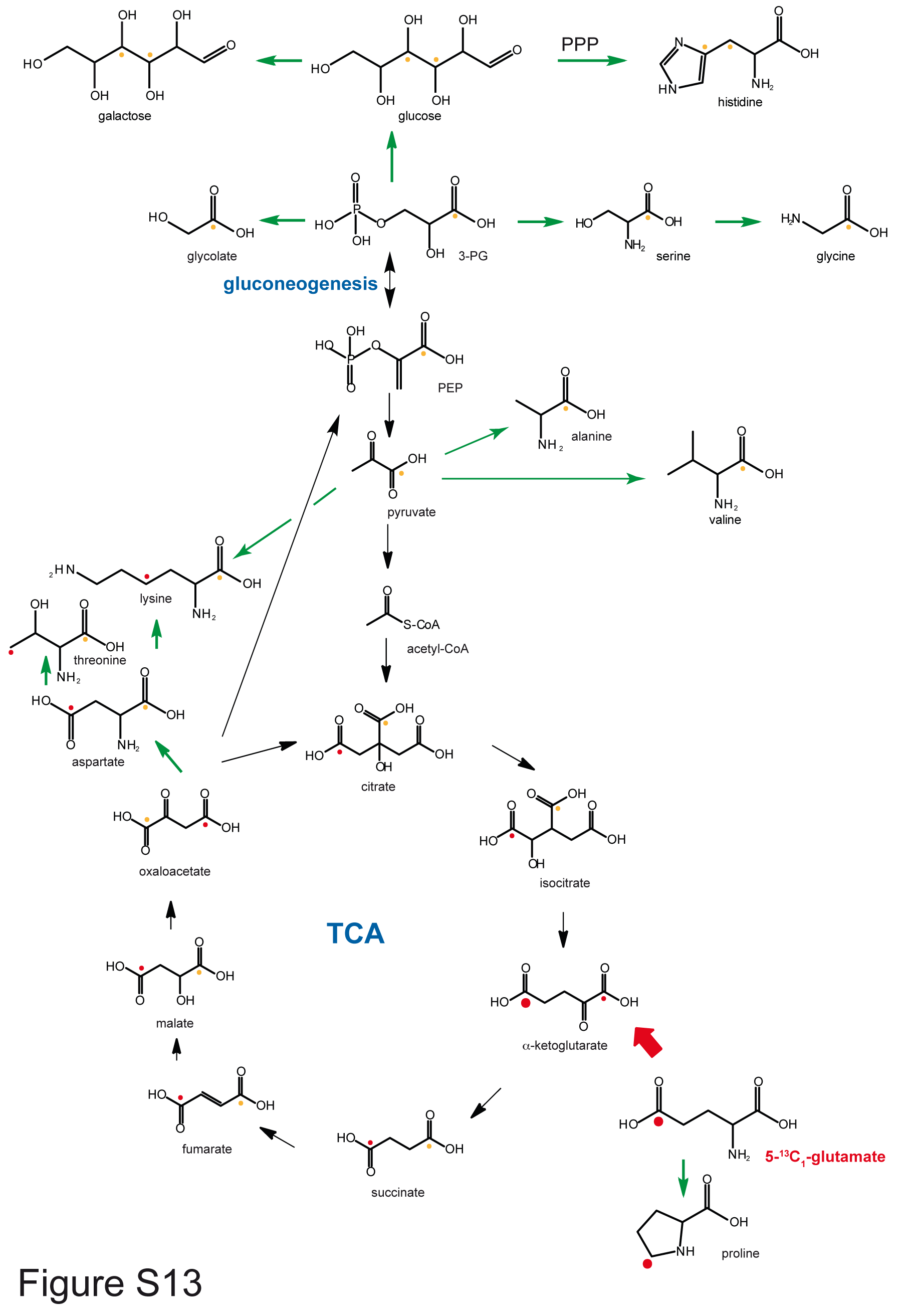

Supplement: S13 Fig — The positions of the 13C-label of the amino acid isotopologues are indicated by the colored dots. Due to the stereoisomerism of the TCA-cycle intermediates succinate and fumarate, it is not possible to distinguish between the C1- or the C4-carbon atom 13C-label. Consequently, the possibilities of the 13C in OAA and its derived amino acids are indicated in red and orange, both colors displaying a 50% possibility that the carbon atom acquired the label. Green arrows depict the synthesis of the amino acids from their respective precursor molecules. The individual reactions of the multi-step amino acid biosynthesis pathways are not displayed. (TIF) [file pbio.2001390.s013.tif]

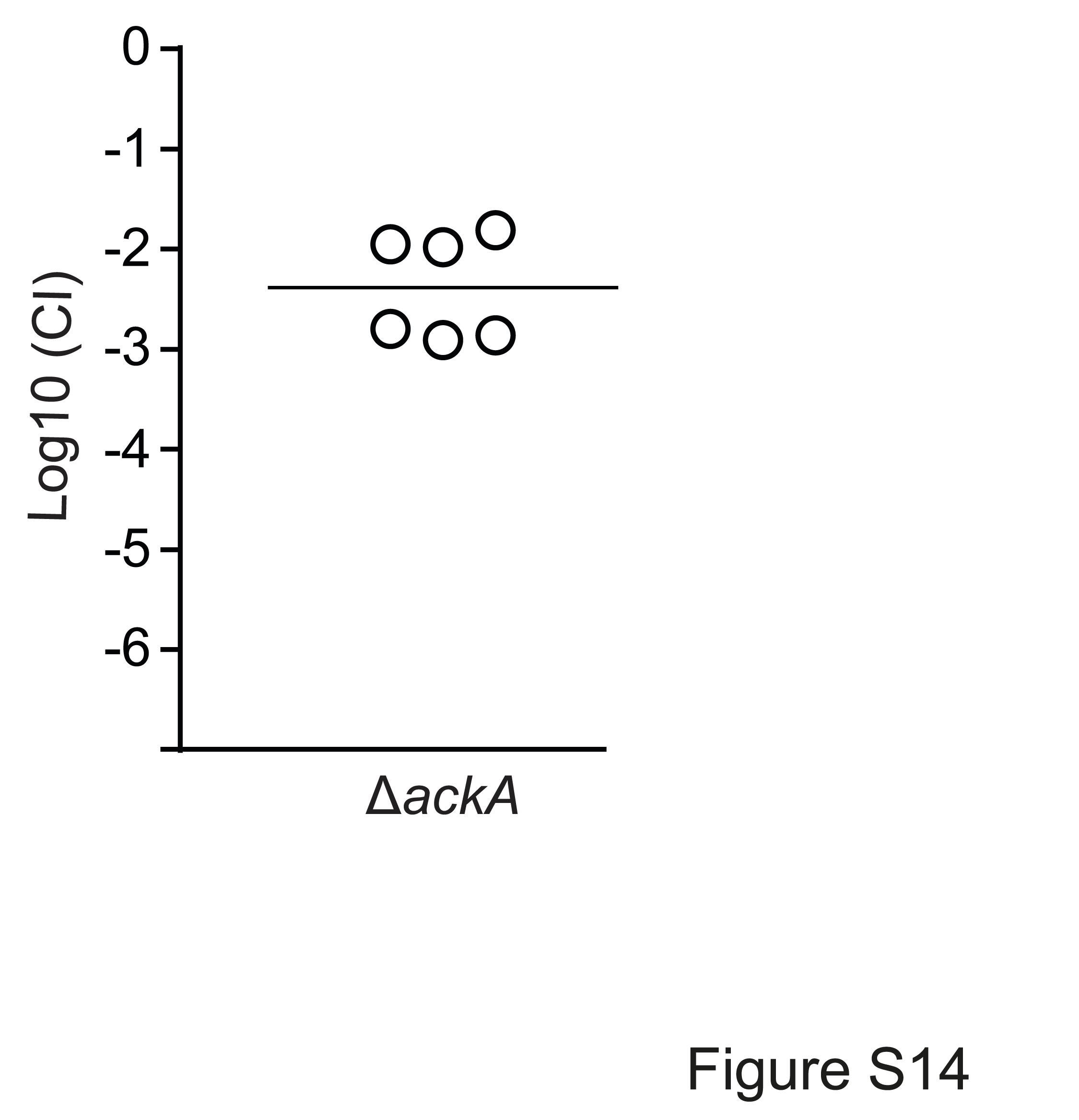

Supplement: S14 Fig — Six mice were inoculated with an equal number of wild-type C. jejuni 81–176 and the ΔackA isogenic mutant strain via oral gavage. Competitive indices (CI) were calculated as the ratio of the CFU of the ΔackA mutant over wild type recovered from the ceca of infected mice (see S14 Table). (TIF) [file pbio.2001390.s014.tif]

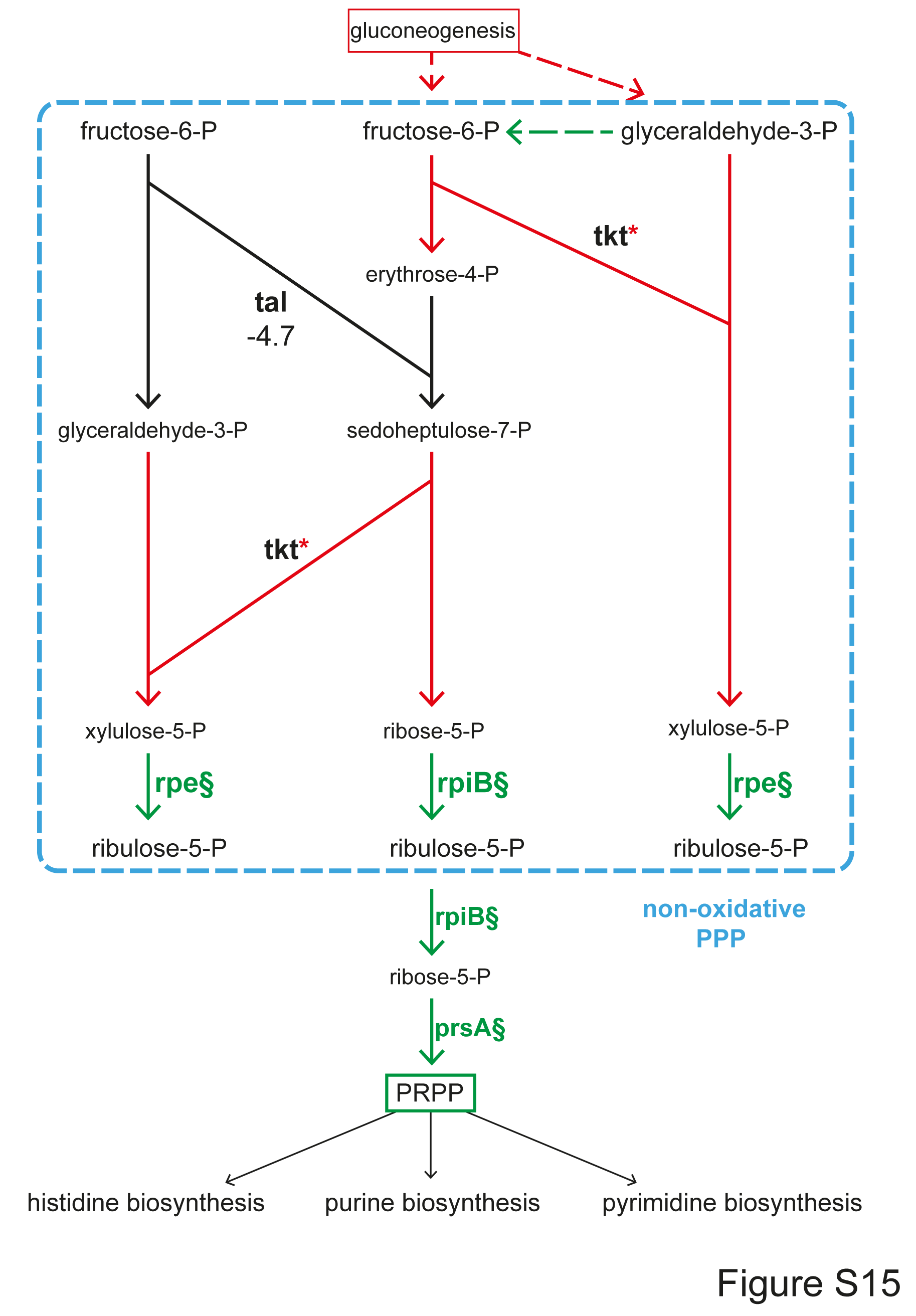

Supplement: S15 Fig — Shown is the impact of the inactivation of genes encoding components of the PPP pathway in C. jejuni mouse intestinal colonization as determined by INSeq analyses. Numbers indicate the log2 value of fold change (intestine/inoculum) in the number of insertions in the indicated genes and are derived from the raw data in S3 Table. Values below -6.2 indicate mutations led to a statistically significant colonization defect. Genes with no insertions in the library are denoted in greeen. *: denotes genes showing a limited number of insertions within the library and no insertions within the pooled of mutants recovered from the intestine. (TIF) [file pbio.2001390.s015.tif]

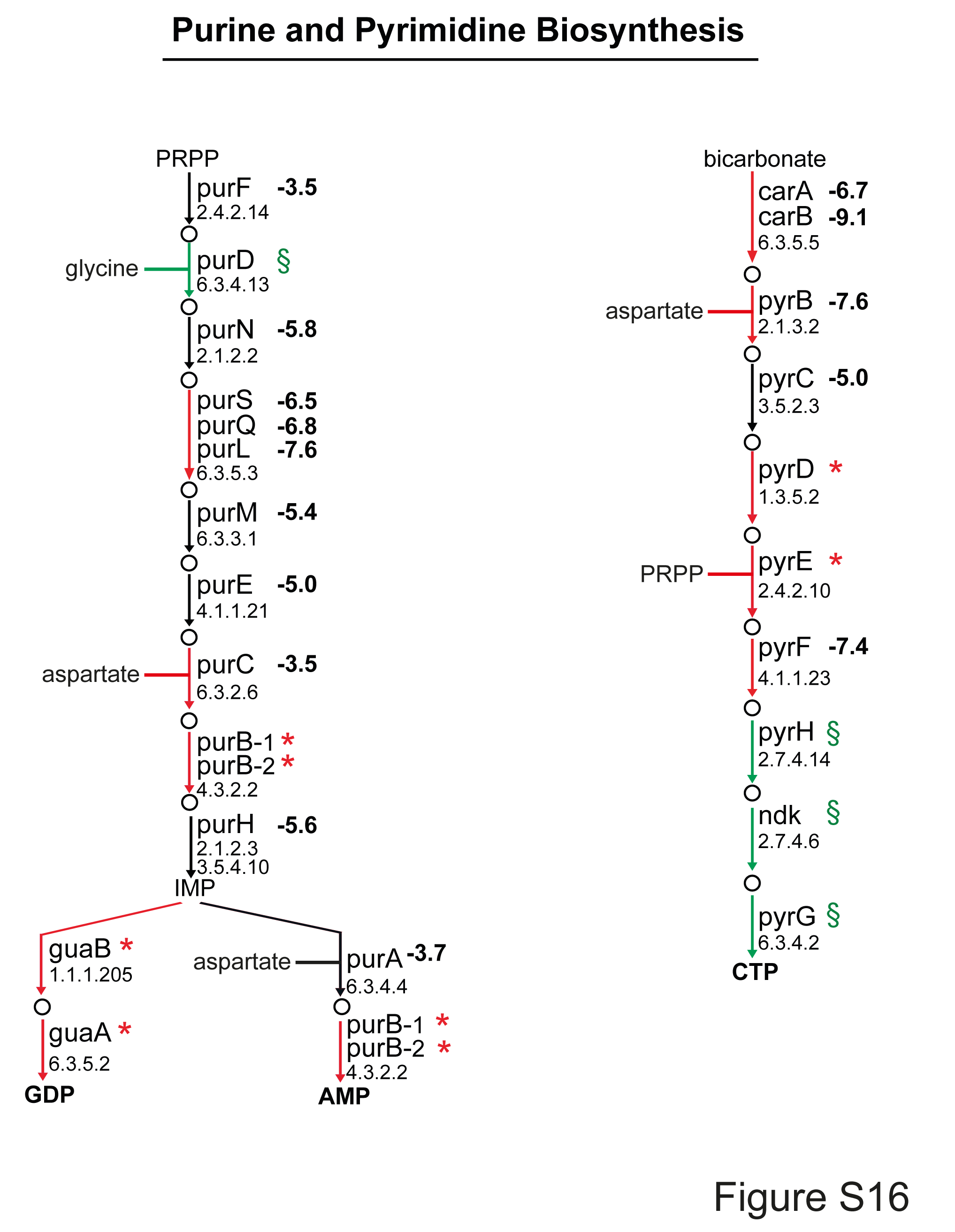

Supplement: S16 Fig — Shown is the impact of the inactivation of genes encoding components of the purine and pyrimidine biosynthesis pathways in C. jejuni mouse intestinal colonization as determined by INSeq analyses. Numbers indicate the log2 value of fold change (intestine/inoculum) in the number of insertions in the indicated genes and are derived from the raw data in S3 Table. Values below -6.2 indicate mutations led to a statistically significant colonization defect. Red arrows denote that the number of insertions within the gene that catalyze the indicated reaction was significantly reduced within the pooled of mutants recovered from the mouse intestine relative to the inoculum. Green arrows indicate that the genes encoding the enzymes that catalyze the corresponding reactions do not have insertional mutants in the library. *: denotes genes showing a limited number of insertions within the library and no insertions within the pooled of mutants recovered from the intestine. (TIF) [file pbio.2001390.s016.tif]

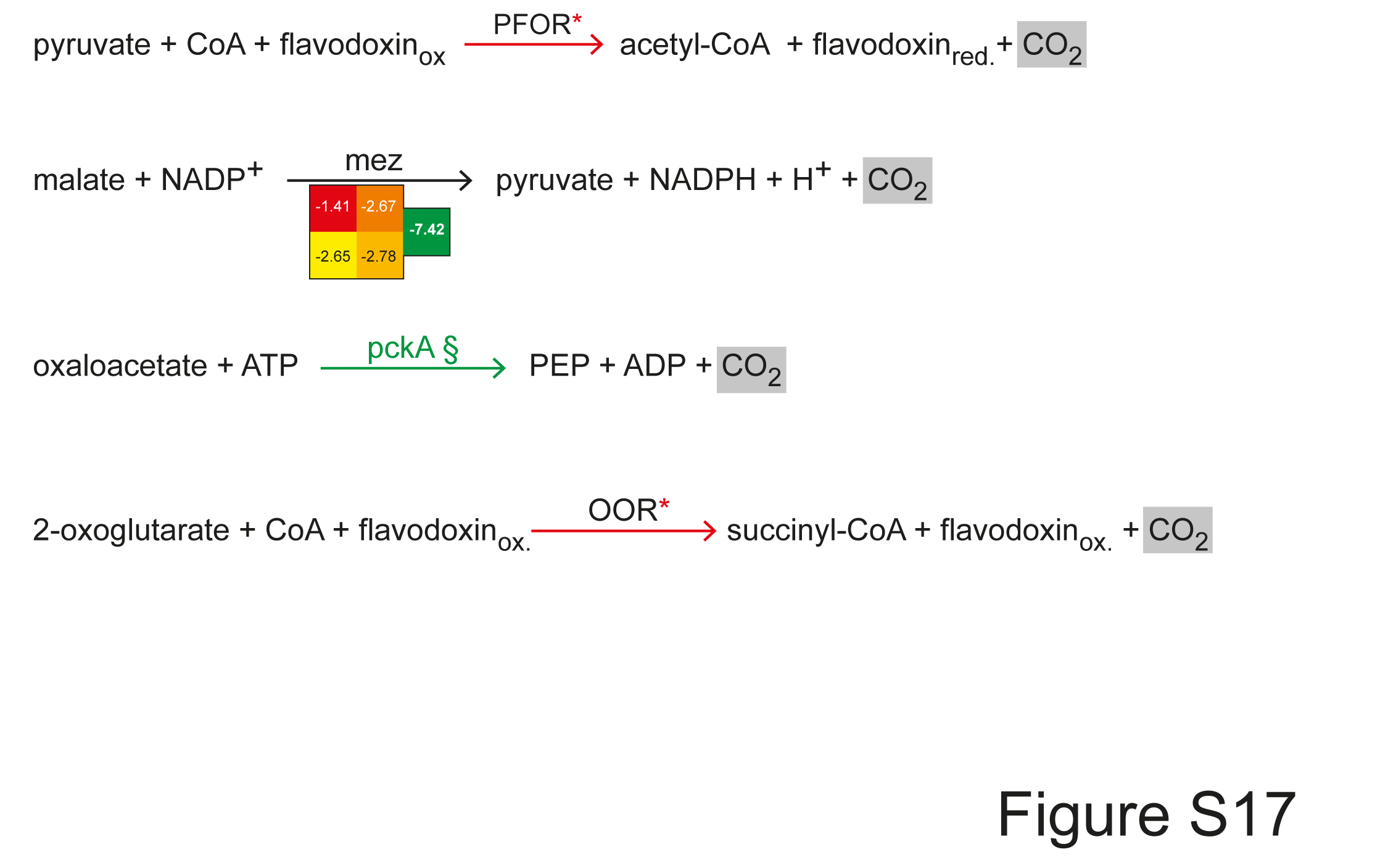

Supplement: S17 Fig — Selected metabolic reactions in C. jejuni that release carbon dioxide (CO2). Numbers indicate the log2 value of fold change (intestine/inoculum) in the number of insertions in the indicated genes and are derived from the raw data in S3 Table. Values below -6.2 indicate mutations led to a statistically significant colonization defect. Red arrows denote that the number of insertions within the gene that catalyze the indicated reaction was significantly reduced within the pooled of mutants recovered from the mouse intestine relative to the inoculum. In green arrows indicate that the genes encoding the enzyme that catalyzes the corresponding reaction are noted genes with no insertions in our mutant library. *: denotes genes showing a limited number of insertions within the library and no insertions within the pooled of mutants recovered from the intestine. (TIF) [file pbio.2001390.s017.tif]
