# Supplementary material for: Metabolic and fitness determinants for in vitro growth and intestinal colonization of the bacterial pathogen Campylobacter jejuni
Source: PLoS Biol. 2017 May 19;15(5):e2001390. doi: 10.1371/journal.pbio.2001390 (PMC5438104; doi:10.1371/journal.pbio.2001390)
Supplement: S6 Table — (DOCX) [file pbio.2001390.s023.docx]

**Table S6. Growth-promoting substrates utilized by *Campylobacter jejuni* during colonization of gastrointestinal tract**

| **Substrate** | | **Catabolic** | **Colonization of intestine** | | | |
| --- | --- | --- | --- | --- | --- | --- |
|  | | **genes** | **mice** | | **chicken** | |
|  | | promoting  colonization | persistence  of infection | persistence  of infection | persistence  of infection | persistence of infection |
|  | |  | < 1 week p.i. | ≥ 1 week p.i. | < 1 week p.i. | ≥ 1 week p.i. |
| **amino acids** | |  |  |  |  |  |
| aspartate | | *peb1A, aspA* | this study | [[1](#_ENREF_1)], [[2](#_ENREF_2)], [[3](#_ENREF_3)] |  | [[4](#_ENREF_4)] |
| asparagine | | *ansB*^a^ | this study | [[5](#_ENREF_5)] |  |  |
| glutamate | | *peb1A* | this study | [[1](#_ENREF_1)], [[3](#_ENREF_3)] |  | [[6](#_ENREF_6)] |
| glutamine | | *ggt*^a^ | this study | [[7](#_ENREF_7)] | [[8](#_ENREF_8)] | [[8](#_ENREF_8)] |
| proline | | *putP* | this study | [[3](#_ENREF_3)] |  |  |
| serine | | *sdaCA* | this study | [[3](#_ENREF_3)] |  | [[9](#_ENREF_9)] |
| arginine | | *argBDF* | this study |  |  | [[10](#_ENREF_10)] |
| isoleucine | | *livJKM* | this study |  |  | [[11](#_ENREF_11)] |
| leucine | | *livJKM* | this study |  |  | [[11](#_ENREF_11)] |
| methionine | | *metN* | this study |  |  |  |
| threonine | | *sstT* | this study |  |  |  |
| valine | | *livJKM* | this study |  |  | [[11](#_ENREF_11)] |
|  | |  |  |  |  |  |
| **carbohydrates** | |  |  |  |  |  |
| fucose | | *fucP*^b^ |  |  | [[12](#_ENREF_12)] | [[12](#_ENREF_12)] |
|  |  |  |  |  | [[13](#_ENREF_13)] | [[13](#_ENREF_13)] |
| gluconate (respiration) | | Cjj81176_0439 | this study | [[14](#_ENREF_14)] |  | [[14](#_ENREF_14)] |
|  | |  |  |  |  |  |
| **organic acids** | |  |  |  |  |  |
| HCO_3_^-^ (CO_2_) | | *canB* | this study |  |  |  |
| formate (respiration) | | *fdh* | this study |  |  | [[15](#_ENREF_15)] |
| succinate | | *frdA* | this study |  |  | [[16](#_ENREF_16)] |
|  | study suggests substrate as being used by *C. jejuni* while colonizing the intestine; mutations in indicated genes lead to colonization defects | | | | | |
|  | study indicates substrate as not being used by *C. jejuni* while colonizing the intestine; mutations in indicated genes do not lead to colonization defects | | | | | |
|  | no information available | | | | | |

a) strain-specific property of *C. jejuni*; isolates like *C. jejuni* 81-176 harbor the genes for a secreted asparaginase (AnsB^SP^) and a γ−glutamyl-transpeptidase (GGT), which are missing in strains like *C. jejuni* NCTC11168

b) strain-specific property of *C. jejuni*; isolates like *C. jejuni* NCTC 11168 harbor a plasticity region with the fucose permease gene *fucP*, which is absent in strains like *C. jejuni* 81-176

**References**

1. Pei Z, Burucoa C, Grignon B, Baqar S, Huang XZ, et al. (1998) Mutation in the peb1A locus of Campylobacter jejuni reduces interactions with epithelial cells and intestinal colonization of mice. Infect Immun 66: 938-943.

2. Novik V, Hofreuter D, Galan JE (2010) Identification of Campylobacter jejuni genes involved in its interaction with epithelial cells. Infect Immun 78: 3540-3553.

3. Hofreuter D, Mohr J, Wensel O, Rademacher S, Schreiber K, et al. (2012) Contribution of amino acid catabolism to the tissue specific persistence of Campylobacter jejuni in a murine colonization model. PLoS One 7: e50699.

4. Guccione E, Leon-Kempis Mdel R, Pearson BM, Hitchin E, Mulholland F, et al. (2008) Amino acid-dependent growth of Campylobacter jejuni: key roles for aspartase (AspA) under microaerobic and oxygen-limited conditions and identification of AspB (Cj0762), essential for growth on glutamate. Mol Microbiol 69: 77-93.

5. Hofreuter D, Novik V, Galan JE (2008) Metabolic diversity in Campylobacter jejuni enhances specific tissue colonization. Cell Host Microbe 4: 425-433.

6. Flanagan RC, Neal-McKinney JM, Dhillon AS, Miller WG, Konkel ME (2009) Examination of Campylobacter jejuni putative adhesins leads to the identification of a new protein, designated FlpA, required for chicken colonization. Infect Immun 77: 2399-2407.

7. Hofreuter D, Tsai J, Watson RO, Novik V, Altman B, et al. (2006) Unique features of a highly pathogenic Campylobacter jejuni strain. Infect Immun 74: 4694-4707.

8. Barnes IH, Bagnall MC, Browning DD, Thompson SA, Manning G, et al. (2007) Gamma-glutamyl transpeptidase has a role in the persistent colonization of the avian gut by Campylobacter jejuni. Microb Pathog 43: 198-207.

9. Velayudhan J, Jones MA, Barrow PA, Kelly DJ (2004) L-serine catabolism via an oxygen-labile L-serine dehydratase is essential for colonization of the avian gut by Campylobacter jejuni. Infect Immun 72: 260-268.

10. Johnson JG, Livny J, Dirita VJ (2014) High-throughput sequencing of Campylobacter jejuni insertion mutant libraries reveals mapA as a fitness factor for chicken colonization. J Bacteriol 196: 1958-1967.

11. Ribardo DA, Hendrixson DR (2011) Analysis of the LIV system of Campylobacter jejuni reveals alternative roles for LivJ and LivK in commensalism beyond branched-chain amino acid transport. J Bacteriol 193: 6233-6243.

12. Stahl M, Friis LM, Nothaft H, Liu X, Li J, et al. (2011) L-fucose utilization provides Campylobacter jejuni with a competitive advantage. Proc Natl Acad Sci U S A 108: 7194-7199.

13. Muraoka WT, Zhang Q (2011) Phenotypic and genotypic evidence for L-fucose utilization by Campylobacter jejuni. J Bacteriol 193: 1065-1075.

14. Pajaniappan M, Hall JE, Cawthraw SA, Newell DG, Gaynor EC, et al. (2008) A temperature-regulated Campylobacter jejuni gluconate dehydrogenase is involved in respiration-dependent energy conservation and chicken colonization. Mol Microbiol 68: 474-491.

15. Weerakoon DR, Borden NJ, Goodson CM, Grimes J, Olson JW (2009) The role of respiratory donor enzymes in Campylobacter jejuni host colonization and physiology. Microb Pathog 47: 8-15.

16. Weingarten RA, Taveirne ME, Olson JW (2009) The dual-functioning fumarate reductase is the sole succinate:quinone reductase in Campylobacter jejuni and is required for full host colonization. J Bacteriol 191: 5293-5300.
