# Supplementary material for: Metabolic and fitness determinants for in vitro growth and intestinal colonization of the bacterial pathogen Campylobacter jejuni
Source: PLoS Biol. 2017 May 19;15(5):e2001390. doi: 10.1371/journal.pbio.2001390 (PMC5438104; doi:10.1371/journal.pbio.2001390)
Supplement: S1 Text — (DOCX) [file pbio.2001390.s032.docx]

**Supporting Methods**

**Isotopologue Profiling**

Growth of *C. jejuni* on ^13^C labeled substrates.

For the isotopologue experiments, *C. jejuni* 81-176 was incubated in DMEM without glucose supplemented with 10 µM ferrous ascorbate. For the experiments with [3-^13^C]serine, the growth medium was supplemented with 4 mM [3-^13^C]serine and 10 mM unlabeled glutamate as energy source. For the experiments with [5-^13^C]glutamate, the growth medium was supplemented with 10 mM [5-^13^C] glutamate and 10 mM unlabelled glutamate. Isotopologue experiments with [NaH^13^CO_3_] were performed in the growth medium with addition of 44mM [NaH^13^CO_3_] to the 44 mM NaHCO_3_, which are already present in DMEM. All experiments were performed in biological duplicates. Therefore 125 ml medium were inoculated with *C. jejuni* 81-176 with a starting OD_600_ of ~0.15, incubated for 48h in jars at 37°C on a rotary shaker (150 rpm) under an oxygen reduced atmosphere (Anaerocult C-Pack; Merck). After 48h of incubation, *C. jejuni* 81-176 cells were killed with 10mM sodium azide and harvested at 5000 x g at 4°C for 30 min. The bacteria were washed three times with cold ddH_2_O and the supernatant was discharged. Finally the bacteria were autoclaved at 120°C for 20 min.

Sample preparation for isotopologue analysis

Protein-derived amino acids

Protein hydrolyzation and amino acid derivatization were performed as described previously [[1](#_ENREF_1)]. Briefly, 5 mg of the bacterial cell pellets were suspended in 0.5 ml of 6 M HCl, and the mixture was incubated for 24 h at 105°C. HCl was removed under nitrogen supply, and 200 µl of glacial acetic acid was added to the dried sample and resolved in an ultrasonic water bath. The hydrolysate was transferred on a minicolumn of Dowex 50WX8 (H^+^ form; 200 to 400 mesh; 0.5 by 1 cm). The column was washed twice with ultrapure water and developed with 1 ml of 4M ammonium hydroxide. An aliquot of the eluate was dried under a stream of nitrogen, and the residue was dissolved in 50 µl of dry acetonitrile. A total of 50 µl of *N*-(*tert*-butyldimethylsilyl)-*N*-methyltrifluoracetamide containing 1% *N*-(*tert*-butyldimethylsilylchloride (TBDMS; Sigma-Aldrich) was added. The mixture was kept at 70°C for 30 min. The resulting mixture of TBDMS amino acids was used for gas chromatography-mass spectrometry (GC-MS) analysis. Asparagine and glutamine were converted into aspartate and glutamate during the acidic hydrolysis. Consequently, the values reported for aspartate and glutamate are mean values for aspartate/asparagine and glutamate/glutamine, respectively. The amino acids tryptophan and cysteine were destroyed during hydrolysis. Arginine does not form TMS or TBDMS derivatives [[2](#_ENREF_2)] and was, therefore, analyzed as arginine-TFA-methylester [[3](#_ENREF_3)]. An aliquot of the cation exchange eluate described above was dried under a stream of nitrogen and dissolved in 200 µl of methanolic HCl (3N). The mixture was heated to 70°C for 30 min and then dried under a stream of nitrogen. The residue was dissolved in 50 µl of trifluoroacetic acid (TFA) and heated to 140°C for 10 min. The mixture was dried again, dissolved in 100 µl of anhydrous ethylacetate and subjected to GC/MS analysis.

Polar, methanol soluble metabolites including fatty acids.

30 mg of *C. jejuni* 81-176 cell pellet (dry weight) were mixed with 0.8 g of glass beeds (0.25-0.5 mm) and 1 ml MeOH and then mechanically disrupted 3x20sec at 6.5 m/sec using a ribolyser (Hybaid, Germany). The suspension was centrifuged at 5,000 rpm for 10 min. The supernatant was brought to dryness under a stream of nitrogen. The residue was dissolved in 100 µl of pyridine containing methoxyamine hydrochloride (20 mg/ml) and reacted for 90 min at 35°C. The reaction mixture was dried under a stream of nitrogen. The residue was dissolved in 100 µl of N-methyl-N-trimethylsilyltrifluoracetamide containing 1% trimethylchlorsilane and heated for 30 min at 50°C. The resulting TMS derivatives were subjected to GC/MS analysis

Bound glucose and galactose derived from cell surface carbohydrates.

The analysis of ^13^C-incorporation into glucose and galactose of cell carbohydrate structures was performed as described previously by Hachey *et al*. [[4](#_ENREF_4)]. Briefly, for methanolysis of cell surface bound sugars, 10 mg of *C. jejuni* cell pellet (dry weight) were dissolved in 0.5 ml of methanolic HCl (3N). The mixture was heated to 80°C for 17 h. After cooling down to room temperature the supernatant was taken and dried under a stream of nitrogen. For derivatisation 1 ml of acetone containing 20 µl of H_2_SO_4_ conc. was added and the solution is kept at room temperature for 1 h. After adding 2 ml saturated NaCl and 2 ml saturated Na_2_CO_3_ solution the mixture was extracted 2 times with 3 ml ethylacetate. The organic phases were combined and dried under a stream of nitrogen. The residue was dissolved in 200 µl ethylacetate:acetic acid anhydride and heated to 60°C for 17h. Afterwards the solution was dried under a stream of nitrogen and the diisopropylidene/acetate derivatives were solved in 100 µl anhydrous ethylacetate for GC/MS analysis. For free glucose 10 mg of *C. jejuni* cell pellet was disrupted in a ribolyser as described above for polar metabolites and analyzed as diisopropylidene/acetate derivative.

**Mass spectrometry and isotopologue analysis**

Gas chromatography mass spectrometry was performed on a GC-QP 2010 plus (Shimadzu, Duisburg, Germany) equipped with a fused silica capillary column (equity TM-5; 30m by 0.25mm,0.25-µmfilm thickness; Supelco, Bellafonte, PA) as described previously [[1](#_ENREF_1)]. The mass detector worked in electron ionization (EI) mode at 70 eV. An aliquot of the amino acid TBDMS derivatives solution was injected in split mode (1:5) at an injector and interface temperature of 260°C. The column was held at 150°C for 3 min and then developed with a temperature gradient of 7°C/min to a final temperature of 280°C. Samples were analyzed in SIM mode at least three times. Data were collected with LabSolution software (Shimadzu, Duisburg, Germany). The overall ^13^C excess values and the isotopologue compositions were calculated by an Excel-based in-house software package according to Lee *et al.* [[5](#_ENREF_5)].

For arginine general GC/MS conditions were the same as described for amino acid TBDMS derivatives. For TFA-methylester derivatives the column was kept at 70°C for 3 min and then developed with a temperature gradient of 10°C/min to a final temperature of 200°C that was kept for 3 min. The retention time for the arginine-TFA-methylester was 17.2 min. The molecular mass of the arginine derivative was 476. ^13^C-excess calculations were performed with m/z 407 [M-CF_3_]^+.^, a fragment still containing all C atoms of arginine. For TMS derivatives of polar intermediates and fatty acids the column was held at 70°C for 3 min and then developed with a temperature gradient of 5°C/min to a final temperature of 310°C. The following metabolites lactate (10.3 min), glycolate (11.1 min), alanine (12.3 min), valine (17.6 min), leucine (20.2 min), isoleucine (21.3 min), glycine (21.7 min), succinate (22.0 min), uracil 23.2 min), serine (24.4 min), methionine (30.3), pyro glutamate (30.8 min), aspartate (31.2 min), threonine (33.1 min), laurate (35.7 min), palmitate (49.0 min) could be detected and isotopologue distribution was calculated with the fragment [M-15 (methyl group from TMS)]^+^**^.^**, which was measured for all metabolites with single ion monitoring

For diisopropylidene/acetate derivatives of sugars the column was held at 150 for 3 min and then developed with a temperature gradient of 10° C/min to a final temperature of 260°C. ^13^C-excess calculations were performed with m/z 287 [M-15]^+.^, a fragment still containing all C atoms of glucose and galactose.

**References**

1. Eylert E, Schar J, Mertins S, Stoll R, Bacher A, et al. (2008) Carbon metabolism of Listeria monocytogenes growing inside macrophages. Mol Microbiol 69: 1008-1017.

2. Halket JM, Waterman D, Przyborowska AM, Patel RK, Fraser PD, et al. (2005) Chemical derivatization and mass spectral libraries in metabolic profiling by GC/MS and LC/MS/MS. J Exp Bot 56: 219-243.

3. Darbre A, Islam A (1968) Gas-liquid chromatography of trifluoroacetylated amino acid methyl esters. Biochem J 106: 923-925.

4. Hachey DL, Parsons WR, McKay S, Haymond MW (1999) Quantitation of monosaccharide isotopic enrichment in physiologic fluids by electron ionization or negative chemical ionization GC/MS using di-O-isopropylidene derivatives. Anal Chem 71: 4734-4739.

5. Lee WN, Byerley LO, Bergner EA, Edmond J (1991) Mass isotopomer analysis: theoretical and practical considerations. Biol Mass Spectrom 20: 451-458.
